# Random walks in handaxe knapping before 1.2 million years ago in East Africa at Melka Kunture (Upper Awash, Ethiopia)

**DOI:** 10.1371/journal.pone.0357880

**Published:** 2026-09-24

**Authors:** Eduardo Méndez-Quintas, Rita T. Melis, Flavio Altamura, Patricia Bello-Alonso, Andrea Serodio Domínguez, Adugna Debalike Belayneh, Riccardo Salvini, Giuliano Ambrosetti, Margherita Mussi

**Affiliations:** 1 Grupo de Estudos de Arqueoloxía, Antigüidade e Territorio (GEAAT), University of Vigo, Facultade de Historia, Ourense, Spain; 2 UNIARQ – Centre for Archaeology, School of Arts and Humanities, University of Lisbon, Lisbon, Portugal; 3 IDEA, Instituto de Evolución en África, University of Alcalá de Henares, Madrid, Spain; 4 Italo-Spanish Archaeological Mission at Melka Kunture and Balchit, Rome, Italy; 5 Dipartimento di Scienze Chimiche e Geologiche, Università di Cagliari, Monserrato, Italy; 6 CNR-IGAG, Institute of Environmental Geology and Geoengineering, Rome, Italy; 7 Forschungsstation Schöningen, Eberhard Karls Universität Tübingen, Schöningen, Germany; 8 Inter-University Graduate School of Human Evolution, University of Burgos, Burgos, Spain; 9 Ethiopian Heritage Authority, Addis Ababa, Ethiopia; 10 Department of Physical Sciences, Earth and Environment, Centre of Geotechnologies CGT, University of Siena, San Giovanni Valdarno, Italy; 11 ISMEO - The International Association for Mediterranean and Oriental Studies. Corso Vittorio Emanuele II, Rome, Italy; Tel Aviv university, ISRAEL

## Abstract

A long-standing objective in Acheulean research is defining evolutionary trends in handaxe production over time. This study investigates technological and morphological variability in the Early Acheulean by analysing three geographically and chronologically proximate assemblages (>1.2 million years ago) from Melka Kunture (Upper Awash, Ethiopia). Combined techno-typological and geometric morphometric analyses reveal significant variability in handaxe morphology, shaping strategies and raw material selection. The observed dissimilarity suggests that Acheulean handaxe manufacture did not follow a linear path of standardisation. Rather, it demonstrates considerable flexibility, reflecting hominins’ responses to varying constraints and immediate needs within short timeframes.

## Introduction

The extensive geographical and temporal development of the Acheulean technocomplex, spanning over 1.5 million years (Ma), has led researchers to suggest the existence of evolutionary traits developing over time. The identification of temporal patterns in knapping skills throughout the Acheulean technocomplex is a recurring topic in the literature [e.g., 1–17].

Among the specific topics that have been thoroughly analysed are handaxes, which are commonly considered the major technological outcome of the Acheulean technocomplex. Efforts have been made to quantify symmetry increase, form standardisation, or tool refinement over time, using different methodological approaches [3,7,9,11,12,14,17–27]. However, fully conclusive results have not been obtained. Instead, other factors are frequently highlighted as offering better explanations for the observed changes, such as the intensity of artefact reduction, variations in raw materials, or the intended functionality of the tools [4,7,10,12,14,28,29]. Most approaches noted in the literature focus on patterns of change over long timescales, while, few studies have examined variability over shorter timeframes [29–31]. The primary aim of our paper is to explore the degree of variability in key handaxe features across geographically and chronologically close sites.

Melka Kunture (Upper Awash, Ethiopia) is a site-complex preserving an extensive sequence of human occupations with Acheulean technology, spanning from its earliest appearance in the global archaeological record, around 1.95 Ma, to the second half of the Middle Pleistocene [32–37]. Around 20 sites and approximately 60 different archaeological layers have been so far identified and explored. In this study, we focus on two sites, one unpublished, Atebella II, and another one only partially published, Simbiro III-Monumental Section [36,38]. Both sites contain a large sample of handaxes (the only currently known assemblages, within the Melka Kunture sequence, from the age range analysed), are geographically and ecologically very close, and share a comparable age, pre-dating 1.2 Ma.

## The sites: Atebella II and Simbiro III-Monumental Section

**Fig 1:**
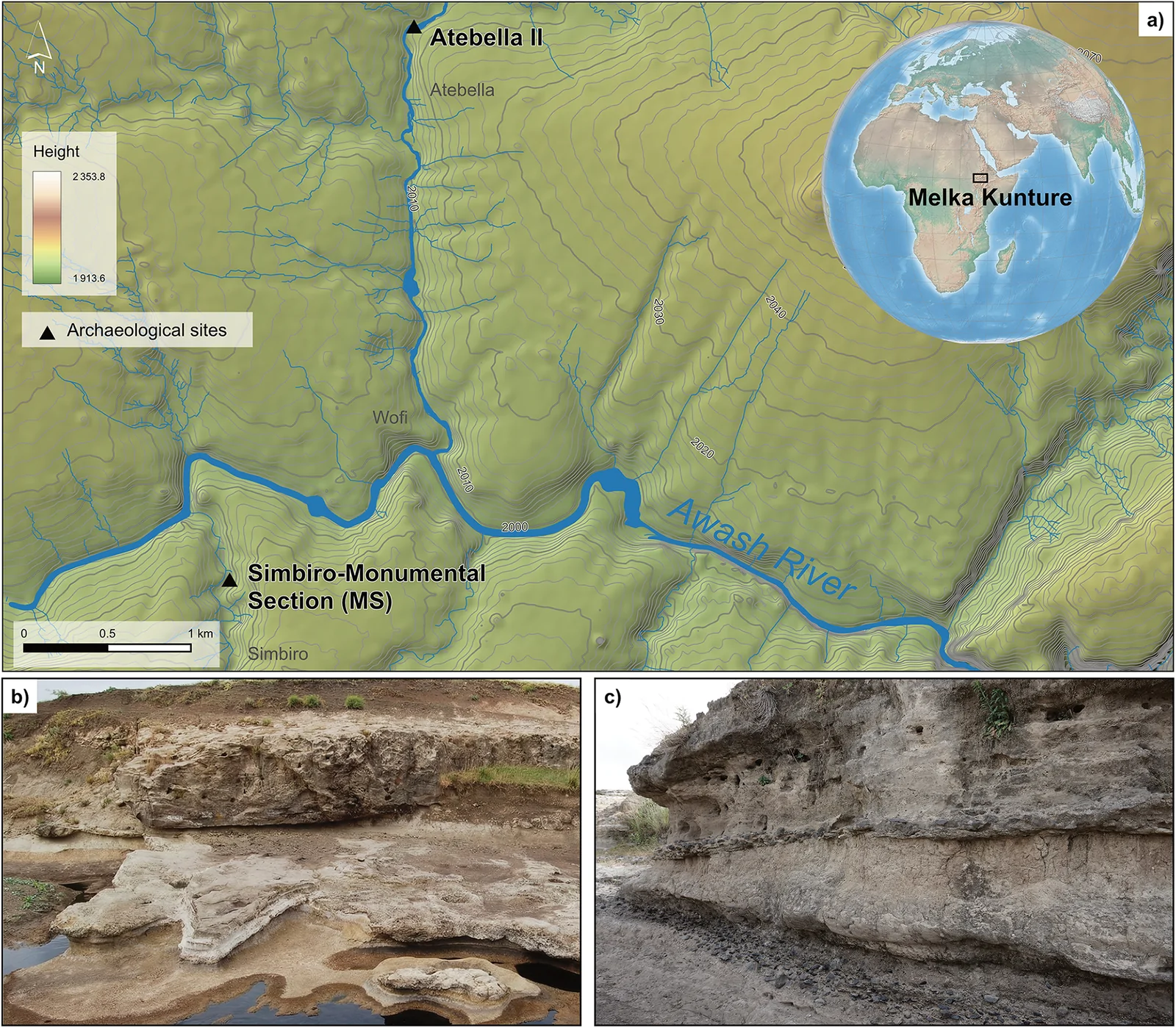
Location map and general view of the sites. Location map of the analysed sites (produced with QGIS software using open-access elevation data provided by JAXA's ALOS World 3D and NASA's NASADEM, via https://opentopography.org, alongside the authors’ own data) (a). General views of the Atebella II (b) and Simbiro-Monumental Section (c) sites. Photographs copyright archive of Italo-Spanish Archaeological Mission at Melka Kunture and Balchit.

The sites of Atebella II (ATE-II) (8.73N, 38.57E, 2013 m.a.s.l.) and Simbiro-Monumental Section (MS) (8.70N, 38.56E, 2013 m.a.s.l.) are located in the Melka Kunture (MK) region, at a distance of 3.5 km of each other as the crow flies, but outside the most intensively researched areas (Fig 1a). Both sites lie on the banks of two eponymous tributaries of the Awash River. Atebella is a left tributary and Simbiro a right one, and both have exposed the sites eroding through Pleistocene deposits (Fig 1a). Both sites are included in the nomination of MK into the World Heritage List.

### Atebella II

**Fig 2:**
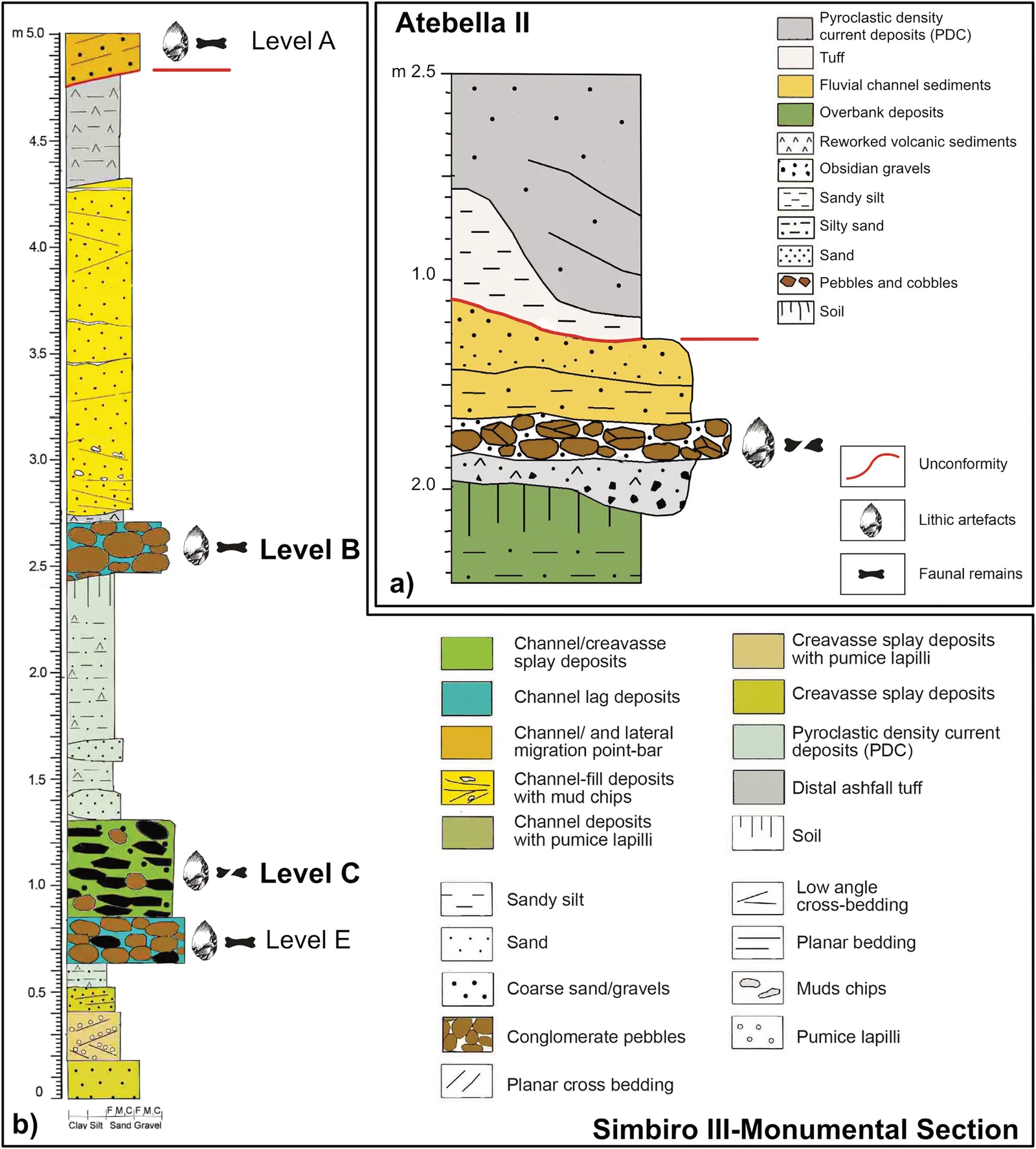
Geological features and chronology of the analysed levels. Stratigraphic logs of the Atebella II (a) and Simbiro-Monumental Section (b) (modified after 36) sites, showing the position of the archaeological levels (created in Affinity Studio software, using own data).

**Fig 3:**
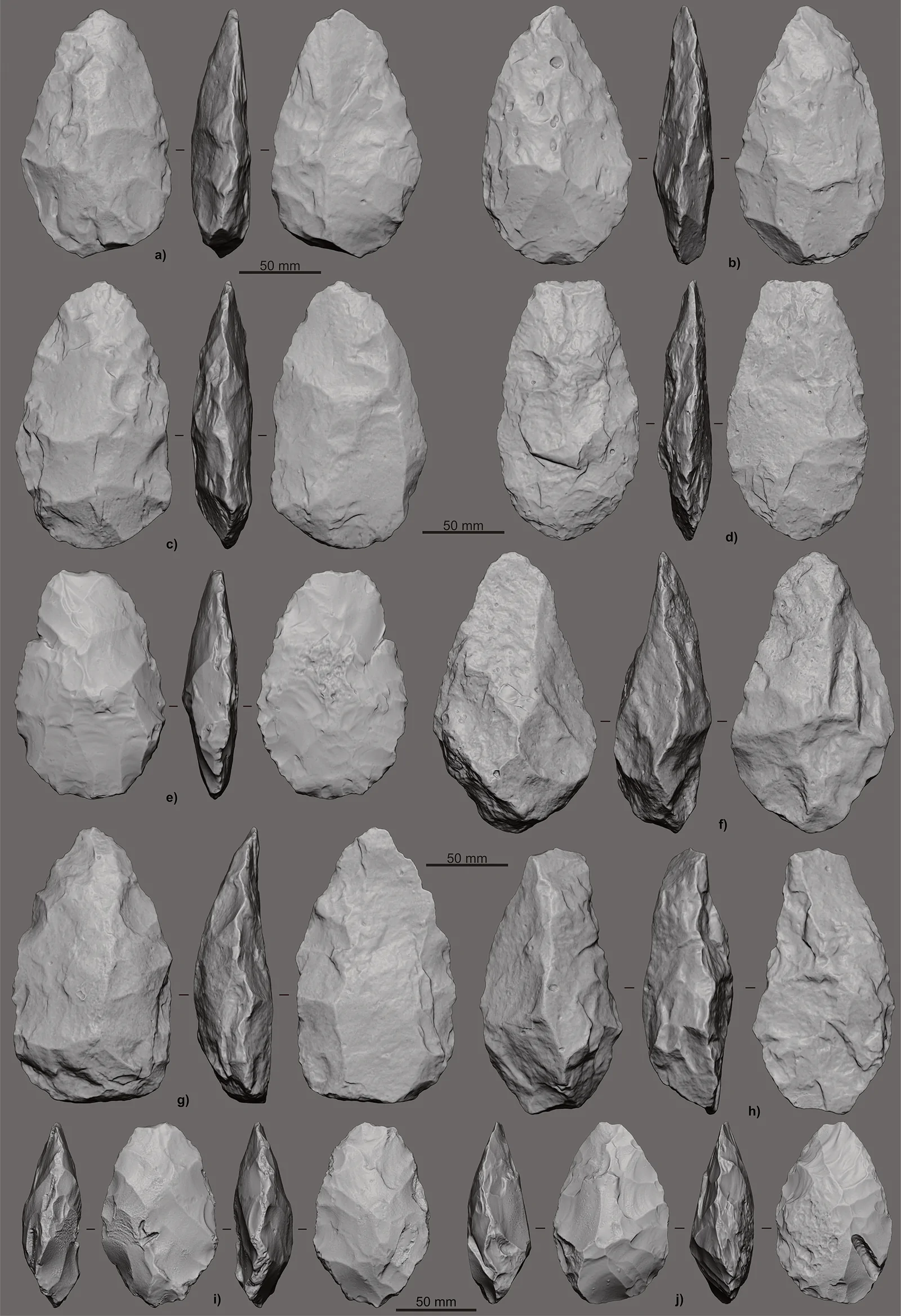
Representative examples of analysed handaxes. a-e: ATE II, f-h: MS Level B and i-j: MS Level C. All pieces are knapped on CVR, with the exception of e, i-j, which are on obsidian (created in Affinity Studio software, using own data).

The Atebella valley has attracted much less research than other areas of MK, like the Garba and Gombore gullies. The area was overlooked during the first explorations of the MK complex by Gerard Bailloud [39] and later on during the surveys of Jean Chavaillon (S1 Appendix). In 2009, during a new survey at the site, a handaxe accumulation was found in full view over several square meters, next to the flowing river (Fig 1b and Fig 1 in S1 Appendix). The archaeological remains were not any more covered by sediments and had been partially eroded by the fluvial action. (Fig 2 and 3 in S1 Appendix). The archaeological layer ATE-II was a dense accumulation of natural clasts and some lithic tools, included in a concretionated matrix with a thickness of c. 0.1–0.2 m (Fig 1b and Fig 2 and 3 in S1 Appendix). An area of 6 m^2^ called ATE-II1 was excavated, yielding 32 lithic pieces, while the complete excavations of the nearby deposit ATE-II2 produced 15 pieces (S2 and S3 Appendix). In our analysis they are lumped together as sample ATE-II. Faunal remains were extremely rare, consisting of only two bone fragments.

### Simbiro-Monumental Section

Like Atebella valley, the Simbiro valley attracted limited interest during the early stages of research at MK [40]. However, in the last few years it became a key-site due to its abundant and exceptional archaeological record [36,38]. The Simbiro stream, has carved a 1,400-metre-long gully, which cuts through a Lower Pleistocene sedimentary sequence (Fig 1a). MS is the most prominent site within the gully (Fig 1c). It was first excavated by J. Chavaillon and O. Oussedik between 1973 and 1976, with further work carried out by M. Piperno in 2004 [40,41] (S5 Appendix). In total, around 18.7 m^2^ of the MS have been excavated revealing five principal archaeological levels, from top to bottom: layers A, B, C, D, and E (Fig 2 in S5 Appendix). A recent archaeological survey furthermore uncovered hundreds of Acheulean tools and faunal remains along over 800 metres of the gully floor and its eroded slopes [38]. The lithic assemblage recovered from the MS comprises 1,556 stone tools and 287 faunal remains, primarily recovered by Chavaillon and Oussedik [36,40,41] (S6 Appendix).

### Geological settings and age

The ATE-II and MS sites are part of the Middle Succession (Early-Middle Pleistocene) as recently defined by Pioli et al. [42], or Melka Kunture and Kella Formations according to Raynal et al. [43] or Salvini et al. [44].

The ATE-II stratigraphy is a c.2.5 m thick fluvio-volcanic sedimentary sequence exposed on the right bank of the Atebella River, 3 km north of the Awash River. The sequence consists of fluvial silty to coarse sandy sediments and pyroclastic density current (PDC) deposits. They overly the lavas and ignimbrites of the late Miocene to Early Pliocene Lower Succession [42] which outcrop along the Atebella river. The stratigraphic section (Fig 2a) starts with floodplain sandy silty sediments pedologically altered at the top. An erosional unconformity separates this deposit from an overlying fluvial flow with debris of obsidian and reworked tuff. The sequence follows upwards with a fluvial channel deposit starting with a lag deposit containing ignimbrite pebbles and archaeological remains embedded in a sandy matrix, overlain in turn by c. 0.4 m thick sandy and silty sediments. The PDC deposit, characterized by an ash bed at the bottom and massive to poorly stratified ignimbrite at the top, covers the sequence. It is well recorded over stretches of the Upper Awash basin [42] and, with the name of Kella Formation, is ^40^Ar/^39^Ar dated to c.1.2 Ma by Morgan et al. [45]. Accordingly, the archaeological level, which deposited before the PDC, predates 1.2 Ma.

The MS stratigraphic section, which exceeds ~5 m in height, comprise a succession of epiclastic and volcaniclastic layers deposited in a fluvial environment, alternating cross-stratified sand beds, massive silt beds and conglomerate lenses [36]. The overall sequence suggests a floodplain with lateral migration of a meandering river and seasonal flooding. The stratigraphic sequence comprises five laterally discontinuous levels, which were labelled from top to bottom A, B, C, D, and E (Fig 2b). The sequence is buried by a massive to roughly stratified tuff, up to 40 cm-thick, cut by a major unconformity visible across the entire outcrop. This unconformity marks the top of the Middle Succession and directly over the unconformity lies the Upper Succession. Accordingly, the archaeological sequence from B to E predates 1.2 Ma, while level A, lying almost directly on the unconformity, postdates it likely after a relatively short interval. The chronology of the whole sedimentary sequence is constrained at the top by a volcanic ash dated to 0.878 ± 0.014 Ma by ^40^Ar/^39^Ar method [45].

## Materials and methods

**Fig 4:**
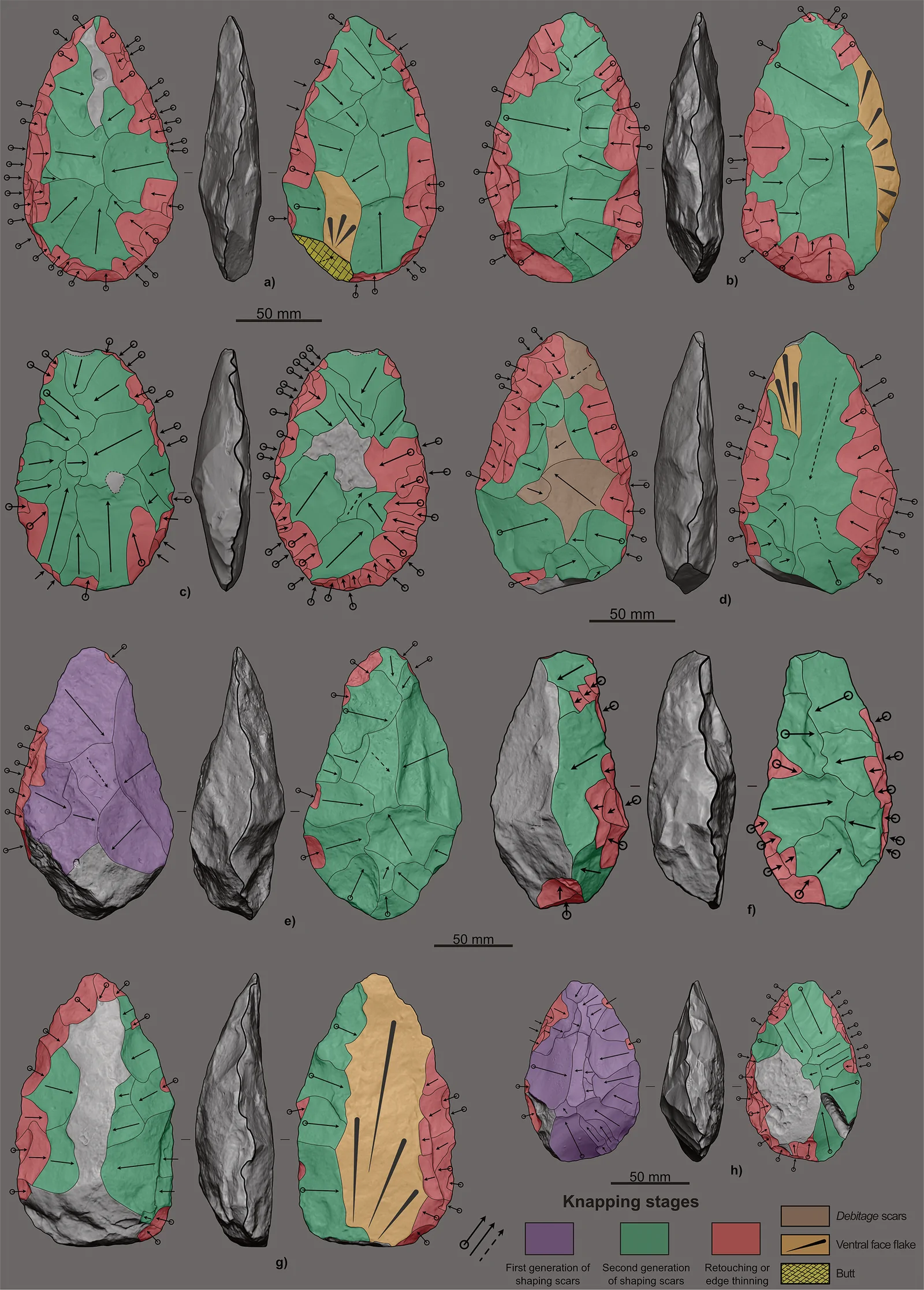
Diacritical diagram of some handaxes. ATE-II (a-d), MS Level B (e-g) and MS Level C (h). All handaxes are knapped on CVR, except c and h which are on obsidian (created in Affinity Studio software, using own data).

The overall handaxe assemblage analysed here consists of 64 pieces (ATE-II = 18, MS Level B = 33, and MS Level C = 13), a sample that appears large enough to examine feature variations across levels (Figs 3 and 4, S7 Appendix). The analysis follows standard methodological approaches (e.g., diacritical diagram) and nomenclatures used in the study of these tools in East Africa and other regions of the world [2,8,46–48]. We opted to identify the support, measure the degree of support transformation, identify the form according to Bordes nomenclature [48], and obtain several size measurements (S8 Appendix). We also analyse the parts of the handaxes were extensive shaping occurred. We characterize this by describing the intensity of shaping quantifying the number of scars and the Scar Density Index (SDI) [49]. We describe the degree of modification of the original contour of the support using a shaping (SI) and retouching index (RI). These values are obtained by the ratio between the total contour length of the artefact and the length of the shaped/thinned edge (S8 Appendix).

We conducted statistical tests to describe and compare the resulting data. Continuous data (e.g., measurements, weights) were analysed using common descriptive statistics (mean, median, standard deviation and range) and were compared across groups using non-parametric tests such as the Kruskal-Wallis and Dwass-Steel-Critchlow-Fligner. For categorical variables, differences between groups were primarily tested using the Chi-Square (χ²) test. Furthermore, a quantitative approach based on bivariate correlation analysis was implemented to evaluate the relationship between size variables and shaping index. Given that the data did not strictly conform to a normal distribution, parametric statistics were precluded. Instead, Spearman’s rank correlation coefficient (*ρ*) was employed to determine the strength, direction, and significance of the associations between the variables under study. The analyses were performed using the statistical software Jamovi [50].

In addition to the technological, shaping-intensity, and size analyses, we assessed the degree of symmetry and shape variation within the handaxe assemblage. For the symmetry degree we used the methodology of Hardaker and Dunn [51], while 2D Geometric Morphometric analysis (GMA) was employed to evaluate morphological variation. The 2D GMA was conducted on all whole pieces and for each handaxe, 28 landmarks were measured and distributed proportionally. The 2D landmark measurement was performed using tpsUtil and tpsDig opensource software [52,53].

The landmark dataset was processed following the Generalised Procrustes Analysis (GPA) in MorphoJ [54] which minimises differences between landmark positions. To start describing shape diversity among datasets we then performed ordination tests: Principal Components Analysis (PCA) and Canonical Variates Analysis (CVA). These procedures explore shape variability, but do not test hypotheses about differences between groups. PCA are complemented by CVA, which produces variables (i.e., canonical variates) to describe and maximise the relative position (i.e., difference) of groups [55]. To validate the results, we used additional robust statistical tests, the pairwise comparisons through Discriminant Function Analysis (DFA) [56]. These results are based on the discriminant function, on a cross-validation test based on jackknife procedures and display the degree of statistical “robustness” of the shape difference between the subsets. In addition, the patterns of change between form groups are noted graphically by the “lollipop” graphs.

## Results

### Raw material

**Fig 5:**
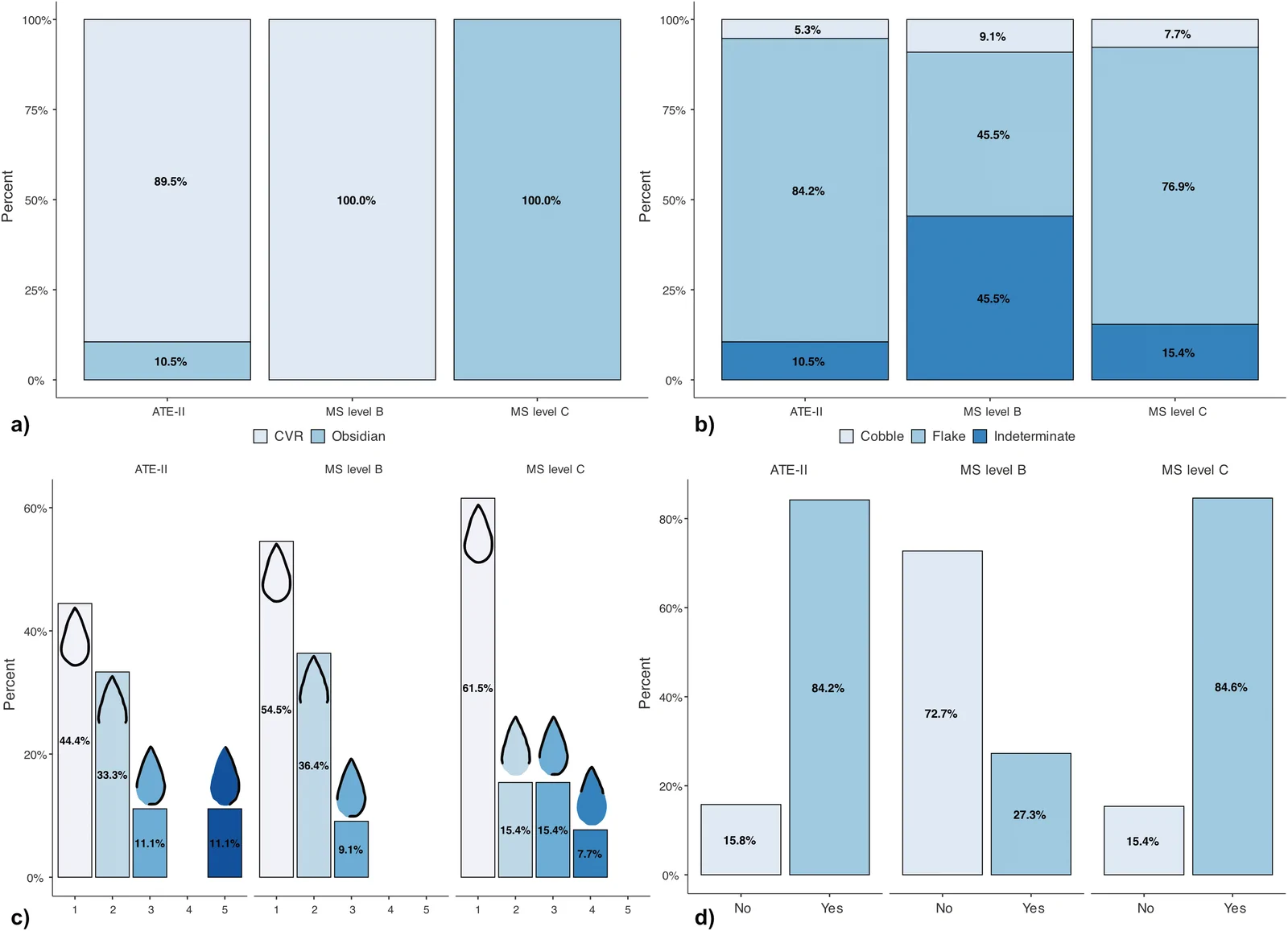
Bar plots of raw material, types of blanks and shaping features. Raw material (a), types of blanks (2), extent of the shaped area (c), and percentage of handaxes with marginal trimming areas (d) within each of the studied assemblages.

The MK region offers a wide range of coarse volcanic rocks (CVR), such as basalts or lavas, suitable for artifact knapping, including a high-quality material such as obsidian [57]. When considering the whole assemblage, the handaxe sample shows a clear preference for CVR, with 76.4% of tools made on those materials and 23.4% on obsidian. However, when we analyse the pattern by levels, we observe clear differences. In ATE-II and MS level B, the predominant raw materials indeed are CVR (89.5% and 100%, respectively), but in MS level C 100% of the handaxes is knapped on obsidian (Fig 5a, Table 1 in S9 Appendix).

### Type of blanks

Variations in the selected blanks is another parameter for assessing changes in handaxe knapping skills. In the overall sample, we observe a clear preference for flakes, which are 62.5% with 29.7% of indeterminate blanks (Table 2 in S9 Appendix). In contrast, cobbles are only occasionally selected (7.8%) (Table 2 in S9 Appendix). The preferential selection of flakes is consistent in the ATE-II and MS level C samples (84.2% and 76.9%, respectively), as is the proportions of other blank types (*X*^*2*^ = 0.269, *p =* 0.87) (Fig 5b, Table 3 in S9 Appendix). However, in MS level B there is an increase of indeterminate blanks (45.5%), and consequently a decrease in the proportion of flakes (45.5%) (Fig 5b, Table 3 in S9 Appendix).

The degree of shaping of handaxes on flakes does not allow determining the features of the dorsal surfaces in 68.3% of cases. When this information can be inferred (n = 13; 31.7%), the blanks do not retain extensive portions of cortex (non-cortical flakes = 46.2%; flakes with <50% cortex = 46.2%). The flaking orientation is mostly indiscernible (n = 21; 52.5%) (Table 4 in S9 Appendix). When it can be identified, most are corner-struck flakes (37.5%), 5.0% are end-struck and side-struck flakes, respectively (Table 4 in S9 Appendix). The same happens with the butts, which are partially or completely removed in 67.5% of cases (Table 5 in S9 Appendix).

### Shaping features

The degree of shaping further allows evaluation of variations in handaxe knapping skills. Overall, the handaxes were extensively shaped, with flaking affecting the entire periphery of the artefact (53.1%) or sparing only a small portion of the base or a lateral edge (31.3% and 10.9%, respectively). Specimens with minimal shaping degree are notably rare (4.7%). This feature remains consistent across the analysed levels (*X*^*2*^ = 11.2, *p =* 0.19) and the main raw material types (*X*^*2*^ = 6.67, *p =* 0.15) (Fig 5c, Fig 1 in S10 Appendix).

In addition to volumetric shaping, the pieces show varying degrees of marginal trimming affecting 56.9% of specimens, though not homogeneously across levels (*X*^*2*^ = 43.8, *p <* 0.05) and raw material types (*X*^*2*^ = 10.8, *p <* 0.05). At ATE-II (n = 16, 84.2%) and MS Level C (n = 11, 84.6%), marginal trimming is usual, whereas at MS Level B, it is reduced (n = 9, 27.3%) (Fig 5d, Table 6 in S9 Appendix). Differences by raw material are also notable, with CVR handaxes showing less marginal trimming (49.0%) than those knapped on obsidian (80.0%) (Table 7 in S9 Appendix).

In artefact shaping, we identified the primary use of hard-hammer percussion (n = 49, 75.4%) and a lower incidence of soft-hammer percussion (n = 15, 24.8%) (Table 8 in S9 Appendix). This trend is maintained in the thinning process, where hard-hammer percussion clearly prevails (n = 50, 76.9%) (Table 9 in S9 Appendix). We observe a clear difference, however, when analysing the sample by sub-sites. While in the MS sites the use of hard-hammer is prevalent, at ATE-II the opposite trend is true, with soft-hammer percussion predominant in both shaping (n = 15, 84.2%) and thinning (n = 15, 78.9%) (Table 8 and 9 in S9 Appendix).

**Fig 6:**
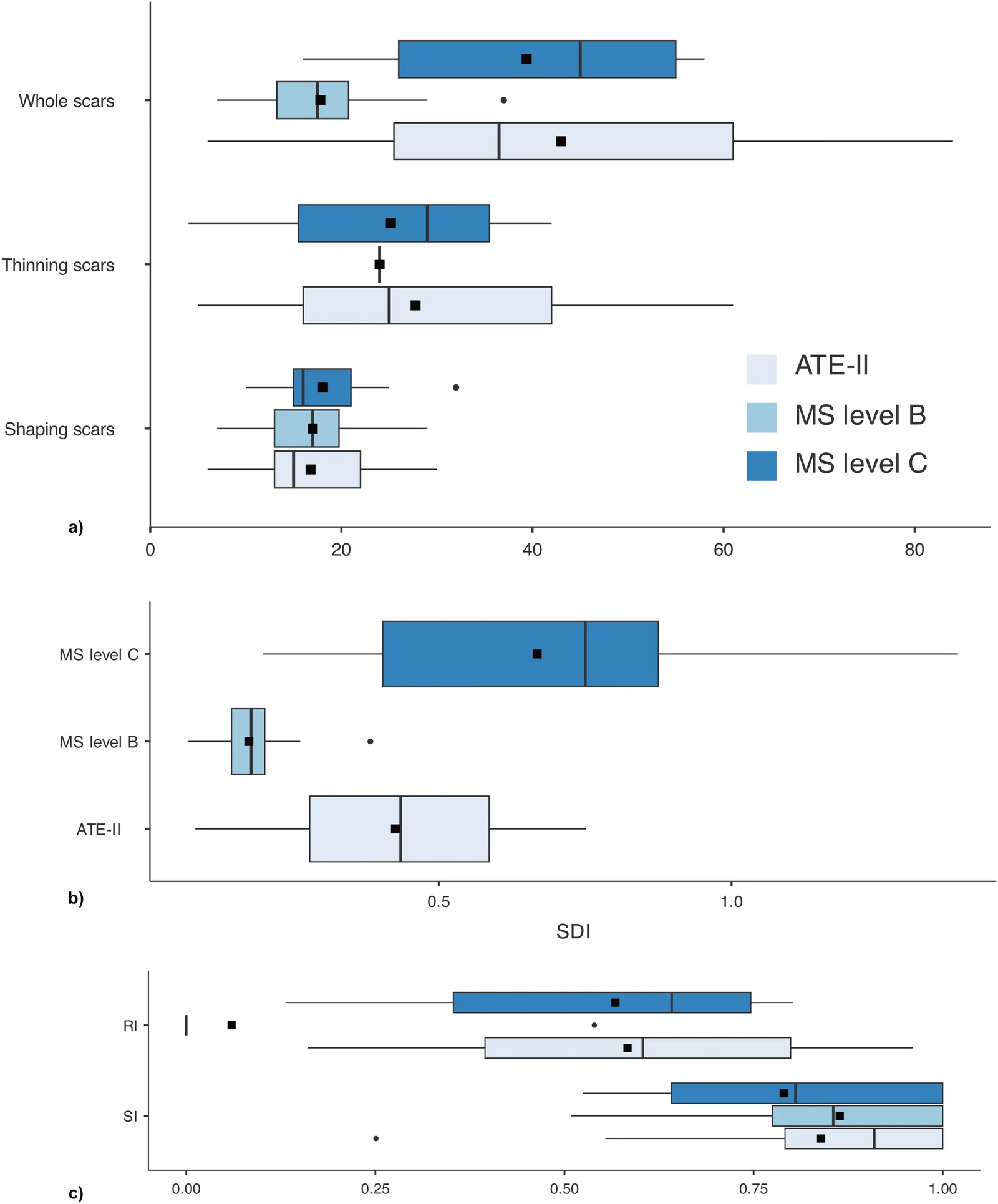
Box plots of the scar number and edges indices. Scar number (a), SDI (b), and edge indices (c) of the analysed handaxes.

The minimum average of whole shaping is 29.8 scars per artefact. Again, some differences are observed among levels. ATE-II and MS Level C both exhibit similar minimum numbers of removals (n = 43.0 and n = 39.4, *p =* 0.80), while MS Level B presents a considerably lower count (n = 16.1, *p <* 0.05) (Fig 6a, Table 10 in S9 Appendix). Substantial differences also emerge by raw material types. Once again obsidian handaxes present more removals than those made from CVR (n = 42.9 and n = 24.0, respectively, *p <* 0.05) (Table 11 in S9 Appendix and Fig 2 in S10 Appendix). When analysing separately the scars associated with shaping and those from edge thinning, differences also emerge (Fig 6a, Table 10 and 11 in S9 Appendix, and Fig 2 in S10 Appendix).

These last features are also discernible using other measurement methods, such as SDI. The SDI calculated for the entire sample is 0.36, with statistically variations (*p <* 0.05) between MS Level B (SDI = 0.15) and ATE-II/MS Level C (SDI = 0.42 and SDI = 0.66, respectively) (Fig 6b, Table 12 in S9 Appendix). The SDI by raw material type displays statistically changes (*p <* 0.05) between rock types (SDI = 0.25 and SDI = 0.77 for CVR and obsidian, respectively) (Table 13 in S9 Appendix and Fig 3 in S10 Appendix).

The average perimeter length is related to different stages of the functional edge knapping. It is 389 mm, with an average of 330 mm shaped and 127 mm marginal trimmed. The values vary significantly across levels (Table 14 in S9 Appendix). Overall, to better assess variations in shaping and retouch impact on handaxe edges, we prefer using the SI and RI index, which eliminate variations imposed by tool size. The SI yields a ratio of 0.84 for the entire sample, with no statistical differences among levels and raw materials (*p =* 0.58, respectively) (Fig 6c, Fig 4 in S10 Appendix and Table 14 and 15 in S9 Appendix). The RI of the entire sample is 0.51 and, bur some major differences by level or raw materials (Fig 6c, Fig 4 in S10 Appendix and Table 14 and 15 in S9 Appendix).

### Symmetry and shape change

The shape of the handaxes is predominantly amygdaloid (n = 37, 57.8%), less frequently oval (n = 11, 17.2%), chisel-edged (n = 7, 10.9%), or of other types (Table 16 in S9 Appendix). There is no substantial difference in type distribution across levels (*X*^2^ = 13.1, *p* < 0.21) or the main raw material type (*X*^2^ = 6.12, p < 0.29) (Table 17 in S9 Appendix).

**Fig 7:**
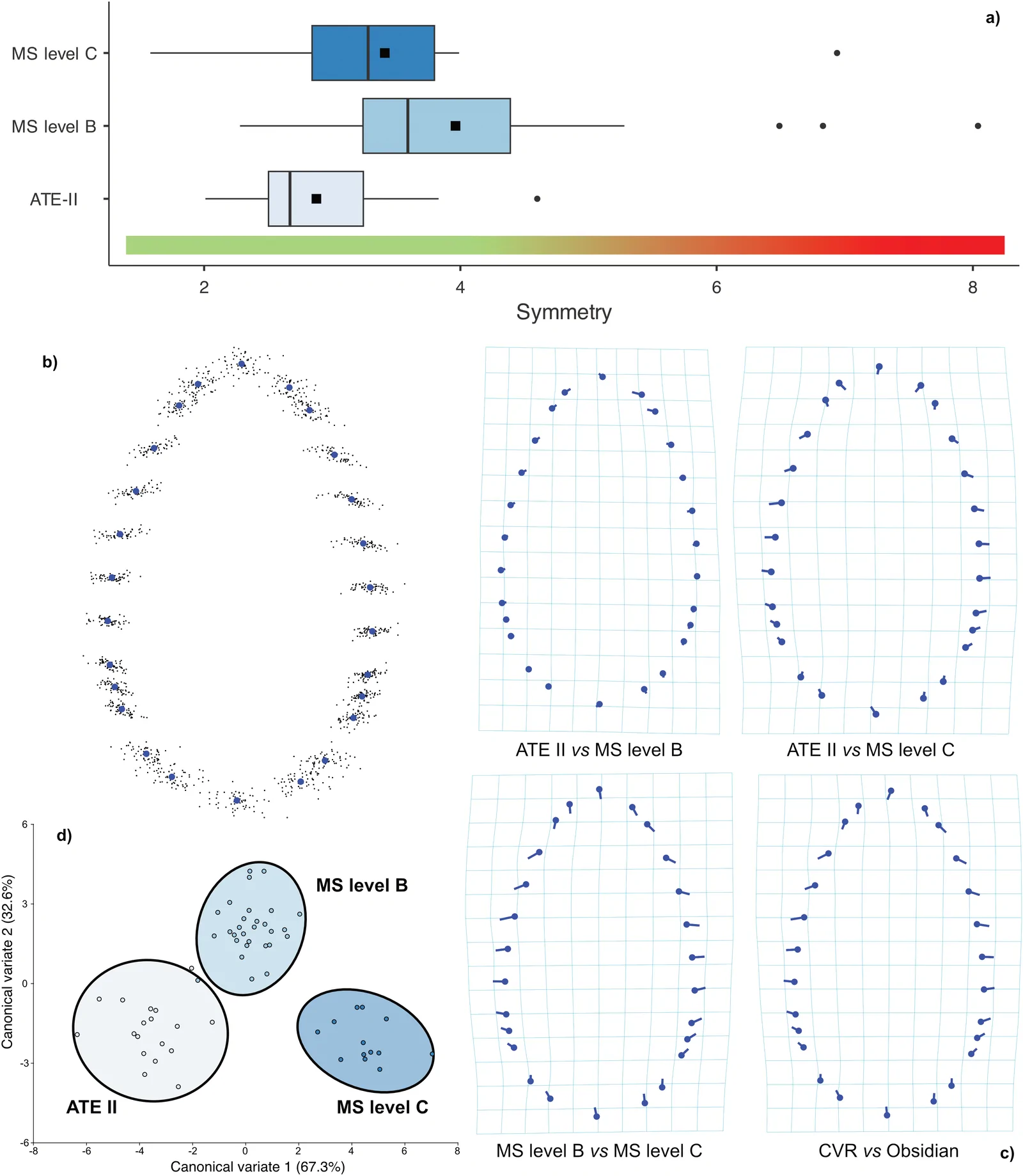
Symmetry and shape variation plots. Box plots with the degree of symmetry -values between 2-3 indicate a high degree of symmetry, whereas values >4 denote moderate to low symmetry- (a), landmark configurations following Procrustes alignment (b); lollipop plots illustrating shape differences between group means (c), and scatter plots of the CVA results (d) of the handaxe analysed assemblages. The 95% confidence ellipses are plotted.

The measurement of handaxe symmetry returns a globally high value (3.5). The highest symmetry values are those of the ATE-II handaxes and, to a lesser extent, of the MS Level C and B handaxes, fluctuating with statistical significance (*p* < 0.05) (Fig 7a, Table 18 in S9 Appendix). ATE-II symmetry values are significantly higher than those of MS Level B (*p* < 0.05), but not markedly different from those of MS Level C (*p* = 0.28) (Fig 7a, Table 18 in S9 Appendix). No statistical differences appear among the MS levels (*p* = 0.42) (Fig 7a, Table 18 in S9 Appendix). Similarly, symmetry values calculated according to the main raw material types do not show statistical differences (p > 0.05) (Table 19 in S9 Appendix, Fig 5 in S10 Appendix).

The PCA results follow GPA (Fig 7b), based on sub-site datasets or raw materials, displaying highly overlapping plots among all data points (Fig 10–11 in S10 Appendix), where the first 10 Principal Components explain 94.7% of the total variance (Table 10 in S9 Appendix). In contrast, the CVA reveals statistical differences in shape among assemblages (Goodall’s *F* = 5.35, *p* < 0.05) and raw material groups (Goodall’s F = 10.69, *p* < 0.05) (Fig 7c). Additionally, both Mahalanobis and Procrustes distances calculated between subsets and raw material groups are statistically different, indicating clear patterns of shape variability (Table 21–24 in S9 Appendix).

The DFA results conclude that none of the comparisons are statistically substantial with respect to differences in shape means (*p* > 0.05), suggesting that the differences among groups are not large enough to be considered systematic (Fig 7d, Table 25 in S9 Appendix). However, the ATE-II assemblage differs more significantly from MS datasets (specifically MS Level C), which retain a higher degree of formal similarity among them (Fig 7d, Table 25 in S9 Appendix). The DFA results based on the main raw material types also show that the differences in means are not statistically clear (*p* > 0.05). However, they allow identifying of some shape differences (Fig 12 in S10 Appendix, Table 25 in S9 Appendix).

### Size values changes

**Fig 8:**
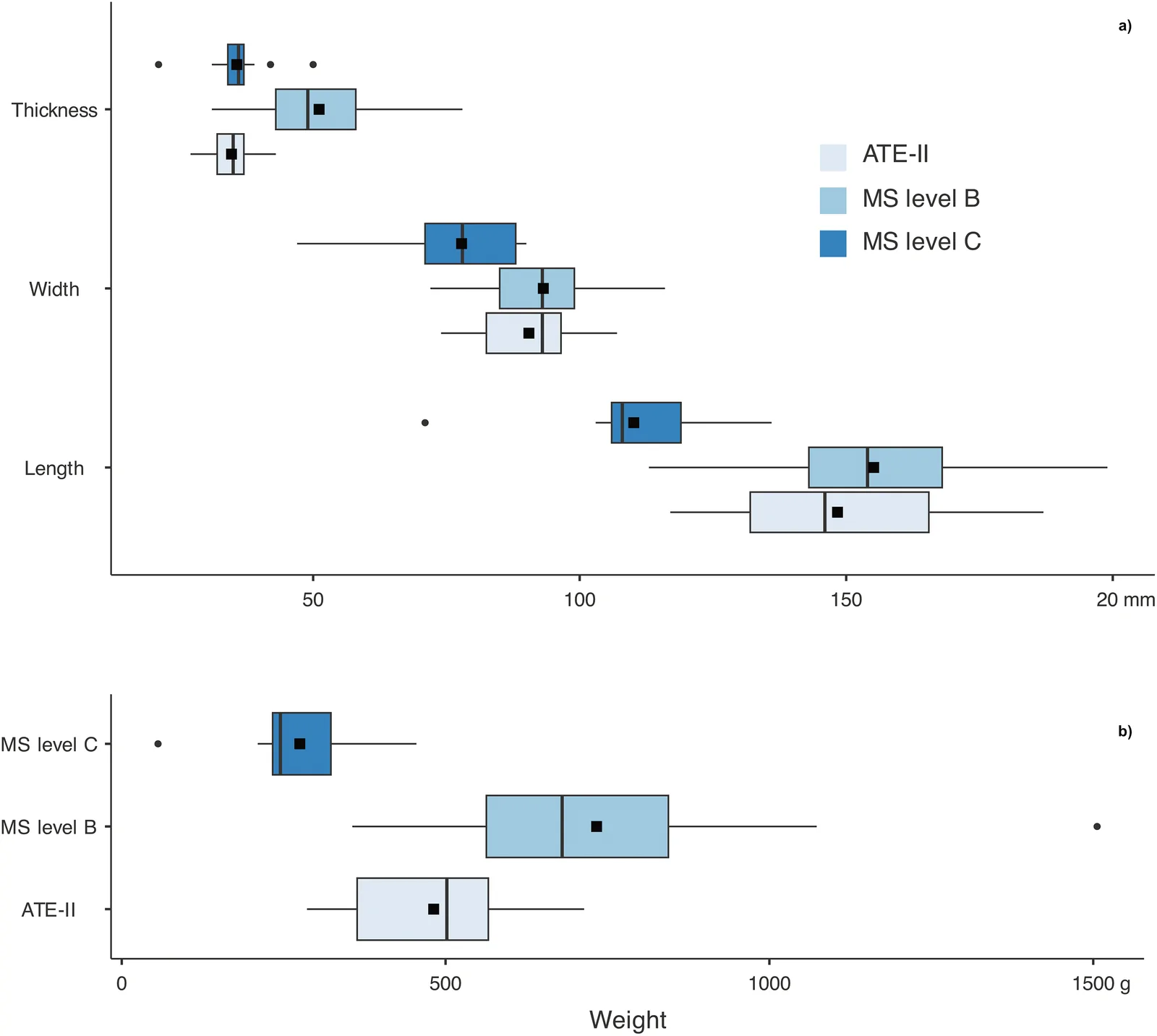
Box plots showing size and weight features. Size (a) and weight (b) values for the handaxes across the studied levels.

The whole handaxe assemblage has average values of 144.0 x 89.3 x 43.3 mm (size) and 568.6 g (weight) (Table 27 in S9 Appendix). The values from the three datasets statistically differ significantly (*p* < 0.05), although with specific characteristics in some pairwise comparisons (Fig 8, Table 28 in S9 Appendix). The most notable feature is the smaller size of the MS Level C dataset compared to the other two (*p* < 0.05) which, in contrast, exhibit similar values to each other (*p* > 0.05) (Fig 8, Table 28 in S9 Appendix). All three datasets are statistically different from each other in weight (*p* < 0.05) (Fig 8b, Table 28 in S9 Appendix). When considering the main raw material types, statistical differences in size and weight values are very clear (*p* < 0.05), with handaxes made from CVR considerably larger than those made from obsidian (Table S34 Table 29 in S9 Appendix, Fig 6 and 7 in S10 Appendix). Finally, according to the type of blank, no major differences appear in size or weight (*p* > 0.05), showing that the measured values are homogeneous across the different blank types (Table 30 in S9 Appendix, Fig 7 and 9 in S9 Appendix).

A Spearman's rank-order correlation was conducted to evaluate size change across symmetry, refinement and reduction intensity (Fig 1–4 in S11 Appendix). The analysis reveals some clear patterns regarding allometry, volumetry, and shaping intensity. The allometric relationship between length and weight remains constant across all subsets (Fig 2–4 in S11 Appendix). However, high symmetry values were achieved without any correlations in changing size, refinement, or the SDI in any subset (Fig 2–4 in S11 Appendix). However, regarding the SDI in MS Level C, the value increase is closely linked to size reduction, as evidenced by negative correlations between the SDI and both length (*ρ* = −0.700, *p* = 0.008) and weight (*ρ* = −0.670, p = 0.015) (Fig 4 in S11 Appendix). In contrast, no such statistically correlation was observed within the ATE II and MS Level B assemblages (Fig 2–3 in S11 Appendix).

Regarding the main raw material types, clear differences were observed in the relationship between size variables and shaping intensity according to Spearman's rank-order correlation. Within the CVR series, higher SDI values are effectively translated into the thinning of the piece. The SDI shows negative correlations with thickness (*ρ* = −0.416, *p* = 0.006) and weight (*ρ* = −0.324, *p* = 0.037), while it does not statistically affect tool length (ρ = −0.101, *p* = 0.525) (Fig 5 in S11 Appendix). Consequently, a clear correlation exists between higher SDI values and increased refinement (*ρ* = −0.378, *p* = 0.014). In contrast, obsidian handaxes exhibit a different pattern. The SDI shows no correlation with thickness (*ρ* = −0.194, *p* = 0.488), indicating that greater shaping intensity does not result in thinner tools (Fig 6 in S11 Appendix). Instead, an increase in SDI impacts overall size, displaying a strong negative correlation with length (*ρ* = −0.640, *p* = 0.010) and weight (*ρ* = −0.586, *p* = 0.024) (Fig 5 in S11 Appendix). These data suggest that the higher degree of shaping in obsidian artefacts leads to a reduction in size, as thinning is hindered without reducing the total length of the handaxe.

In terms of symmetry degree, asymmetry in CVR handaxes (values >4) co-varies with volume retention, specifically, thicker and heavier pieces tend to be significantly more asymmetrical (*ρ* = 0.323, *p* = 0.031) (Fig S45). Conversely, in obsidian handaxes, symmetry is entirely independent of other size and shaping intensity variables (all p-values > 0.804) (Fig 6 in S11 Appendix). This indicates that the mechanical features of obsidian facilitate knapping of more symmetrical artefacts, regardless of the initial size or thickness of the blanks, whereas CVR do not allow it as easily.

## Discussion

The analysis of handaxe samples from sites geographically and chronologically close to each other reveals strong variations. Shaping strategies, tools features, and raw material selection patterns change among them, despite the lack of any large diachrony or different access to raw materials. The artefacts exhibit a substantial degree of modification of the original blank, although the intensity of shaping (e.g., number of scars or SDI), knapping techniques (presence/absence of marginal trimming or soft percussion), or overall size vary substantially across the datasets. ATE-II and MS Level C show a similar degree of thinning and shaping intensity, giving them a “refined” appearance despite markedly different raw material selection patterns. In contrast, MS Level B exhibits a low degree of shaping and, consequently, a more “crude” appearance.

The handaxe shape is primarily pointed, conforming to an amygdaloid design with predominantly sharp cutting bases. However, across subsets symmetry values are not homogeneous. In the case of ATE-II the degree of symmetry is significantly higher than in the other two MS subsets, which retain lower, but mutually comparable values. Furthermore, 2D GMA tests also demonstrate strong and statistically conclusive differences. The MS datasets maintain a lower degree of inter-variability (i.e., a higher degree of inter-assemblage homogeneity) compared to ATE-II, which is morphologically distinct from the MS assemblages.

Overall, the handaxes were shaped on large flakes produced without any predetermined reduction pattern, unlike some cleavers-on-flakes from the same sites. Although there is no direct technological relationship between handaxes and cleavers-on-flakes, it is noteworthy that in the large sample of cleaver-on-flake tools from MS Level B (n = 70), the flake-supports show a high percentage of centripetal/peripheral dorsal scar patterns or fully predetermined flaking schemes [38]. This further suggests a clever selection of flake-supports from standardised cores, facilitating a lower investment of time and technical gestures during last shaping stage. In any case, the preferential use of large flake as blanks for handaxe knapping, alongside cleavers-on-flakes in chronologies prior to 1.2 Ma, further support the understanding that the Large Flake Acheulean (LFA) concept is much older than first conceptualised [58], and is a hallmark of the Acheulean technocomplex ever since its emergence [37].

Hominin groups in the MK region had access to a wide range of volcanic rocks suitable for knapping handaxes and other tool types. Obsidian, of excellent knapping quality was widely available and extensively used, which is exceptional for the entire African Early Stone Age [36]. Despite outright differences in mechanical properties and knapping response among the main raw materials, we note a clear preference for coarse-grained types in handaxe manufacture, except in MS Level C, where the use of obsidian is exclusive. The sample also reveals key differences in the technological behaviour of handaxe knapping linked exclusively to raw material type. Put simply, handaxes on obsidian show more intensive shaping than those on CVR, as well as greater intensity in marginal trimming. However, this is not accompanied by any increase in symmetry, morphological standardisation, or functional edge length, which all show values similar to those of handaxes on CVR.

The preferential selection for handaxes of coarse-grained raw materials over fine-grained ones (accessibility being the same) is not exclusive to the MK region or to this specific Acheulean time range. This behaviour has been noted in other assemblages [46,59,60], pointing to an economic strategy of raw material selection planned according to the types of desired artefact [61]. This suggests that the purpose is not always the maximum cutting efficiency (i.e., edge sharpness), but rather the temporal durability of the edges (i.e., toughness/resistance).

Our analysis, furthermore, detects evidence of soft-hammer percussion in the shaping and marginal trimming of handaxes before 1.2 Ma, i.e., very early. The available information is not highly detailed, but the use of soft-hammers is usually reported from sites later than 1 Ma, as Isenya (Kenya) and Garba I within the MK site-complex [62,63]. The early appearance of this technological solution shows once again that the key features and technological solutions of the Acheulean technocomplex were developed by hominin groups since an early stage. Even if not easily and fully understood, we see behavioural changes and technical choices at any given time, in contrast with early interpretations of Acheulean evolution favouring the unilinear and progressive increase in knapping skills and artefact refinement.

Within the African record, in age ranges similar to those described here (pre-1.2 Ma), there are more cases of handaxe variability over short timeframes. Among the best-known examples are the TK site at Olduvai Gorge (Tanzania), dated 1.3 Ma. In the Lower Occupation Floor, an extensive number of massive, primarily pointed handaxes have been recovered, shaped with a low number of removals but featuring specific areas for prehension and use which suggest a well-defined function [64]. Conversely, in an immediately overlying level (*Sivatherium* floor) with abundant large mammal remains, the handaxes show very different features, mainly in size and shape, which appear directly related to performed activities [65]. Elsewhere at Olduvai Gorge, over similar or earlier chronologies [29,30,66], these differences in handaxe outputs are also common, defining a strong variability linked to changes in functionality between sites and/or levels.

The main conclusion to be drawn from the analysis of pre-1.2 Ma assemblages of the MK site-complex, as well as from the record of other East African sites, is that handaxes exhibit a high degree of variability over restricted temporal ranges. This is in sharp contrast with analyses based on trans-regional analyses and/or extensive time ranges and suggesting patterns of temporal evolution in knapping skills or handaxe “refinement” [9,11,12,14,17,20–23,26].

The technological solutions appear stable and temporally cohesive in the African Acheulean and beyond it, without any major leap or chronological gap, that would allow the identification of traits of progressive evolution. Instead, site functionality and accessible raw material types play a decisive role in tool output.

## Supporting information

S1 Appendix Atebella II: history of research. (PDF)

S2 Appendix The lithic tool assemblage from ATE-II. (PDF)

S3 Appendix Stone tool use-wear data. (PDF)

S4 Appendix Photogrammetric recording at ATE II. (PDF)

S5 Appendix History of research at Simbiro-Monumental Section. (PDF)

S6 Appendix The lithic tool assemblage from MS at Simbiro. (PDF)

S7 Appendix Edge length (a), size (b) measurements and indices and refinement index (c) [2] followed in this manuscript. (TIFF)

S8 Appendix Representative handaxes from the studied assemblages and supplementary diacritical schemes. (PDF)

S9 Appendix Additional tables. (PDF)

S10 Appendix Additional plots. (PDF)

S11 Appendix Correlation matrix results and plots. (PDF)

S12 Appendix Measurements and metric data of the analyzed handaxes. (OMV)

S13 Appendix File containing the 2D landmark coordinates of the analyzed handaxes. (DAT)
