# Supplementary material for: Random walks in handaxe knapping before 1.2 million years ago in East Africa at Melka Kunture (Upper Awash, Ethiopia)
Source: PLoS One. 2026 Sep 24;21(9):e0357880. doi: 10.1371/journal.pone.0357880 (PMC13606939; doi:10.1371/journal.pone.0357880)
Supplement: Supplementary file 1 [file pone.0357880.s001.pdf]

S1 Appendix. Atebella II: history of research

Jean Chavaillon, who directed the archaeological research at Melka Kunture in the second half of the 20th century, briefly surveyed the Wofi area, at the mouth of the Atebella tributary between 1966 and the beginning of the 1970s. At the Wofi I-IV sites, Late Stone Age industry was found in numbers, while Acheulean and Middle Stone Age implements were less well documented (1). Upstream, the proper Atebella area was likely noticed at the time but there is no related note or picture in the archives of the archaeological mission.

In 2006, a first systematic survey directed by Marcello Piperno, who had become in 1999 the director of research at MK, led to the discovery of several Pleistocene sites on the right bank of the river, including ATE-II (**Fig. 1a**). Excavations eventually started in 2011 when Margherita Mussi became in turn director of the research, with the aim of salvaging the archaeological remains which laid exposed and avoiding further damage by animal trampling and flood. A 3D documentation of the surface and sections was also prepared, using recording techniques which, at the time, were still of some complexity (**S4**, **S4 Fig 4-5**).

Carmine Collina was in charge of the excavations of ATE-II (**Fig. 1b**), together with Flavio Altamura. The archaeological level outcropped at the base of a 3 m-tall geological section, at the western bottom of the stream bed. The main deposit, called ATE-II1, was extended over a sub-planar surface of c. 6 m^2 (**Fig. 2-5**). The western portion of the level, and probably part of its surface, had been wiped away by water erosion. The deposit continued below the western section of the riverbed. About 1 m south of the outcrop, a test pit was excavated over 1 m^2 along the standing section reaching the archaeological level, which here was found undisturbed and named ATE-II2 (**Fig. 2-5**).

Fig. 1. General view of the ATE-II site (a) and ongoing excavations in 2011 (b). Photographs copyright archive of Italo-Spanish Archaeological Mission at Melka Kunture and Balchit.

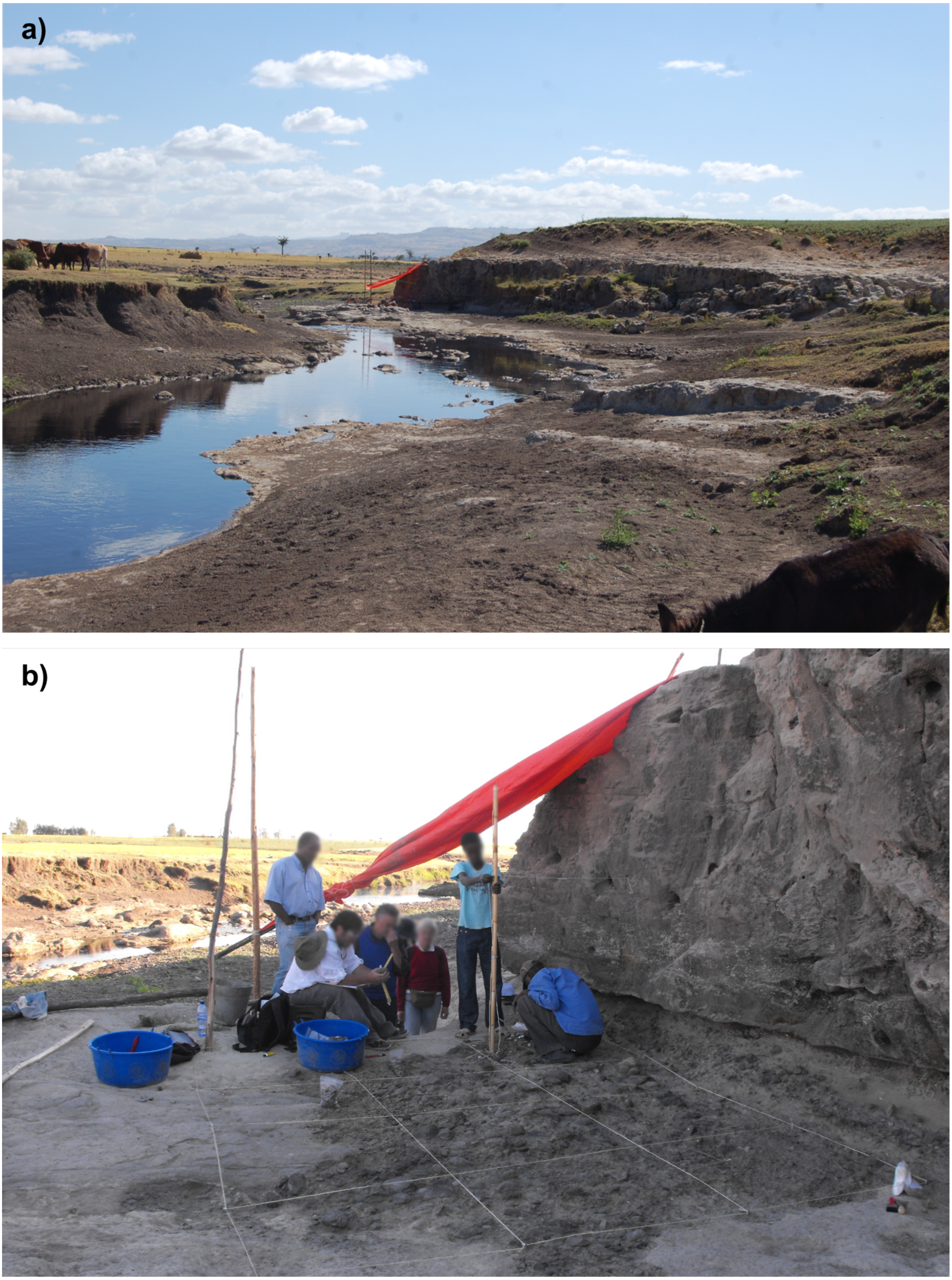

Fig. 2. Sectors ATE-II1 (a) and ATE-II2 (b), located below the PDC level dated to c. 1.2 Ma**.** Photographs copyright archive of Italo-Spanish Archaeological Mission at Melka Kunture and Balchit.

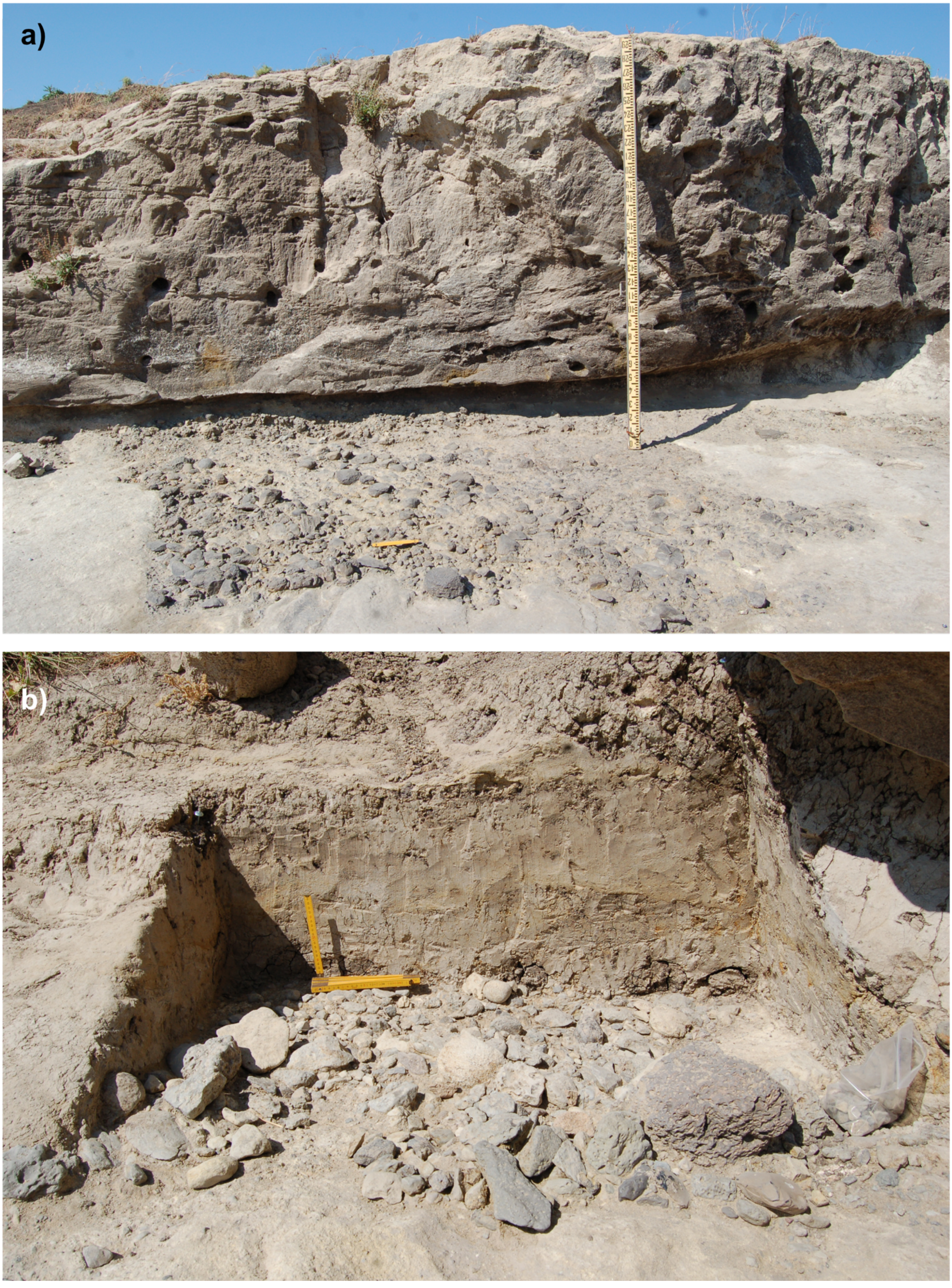

Fig. 3. General view (a) and detail (b) showing handaxe accumulations (white arrows) identified in sector ATE-II1. Photographs copyright archive of Italo-Spanish Archaeological Mission at Melka Kunture and Balchit.

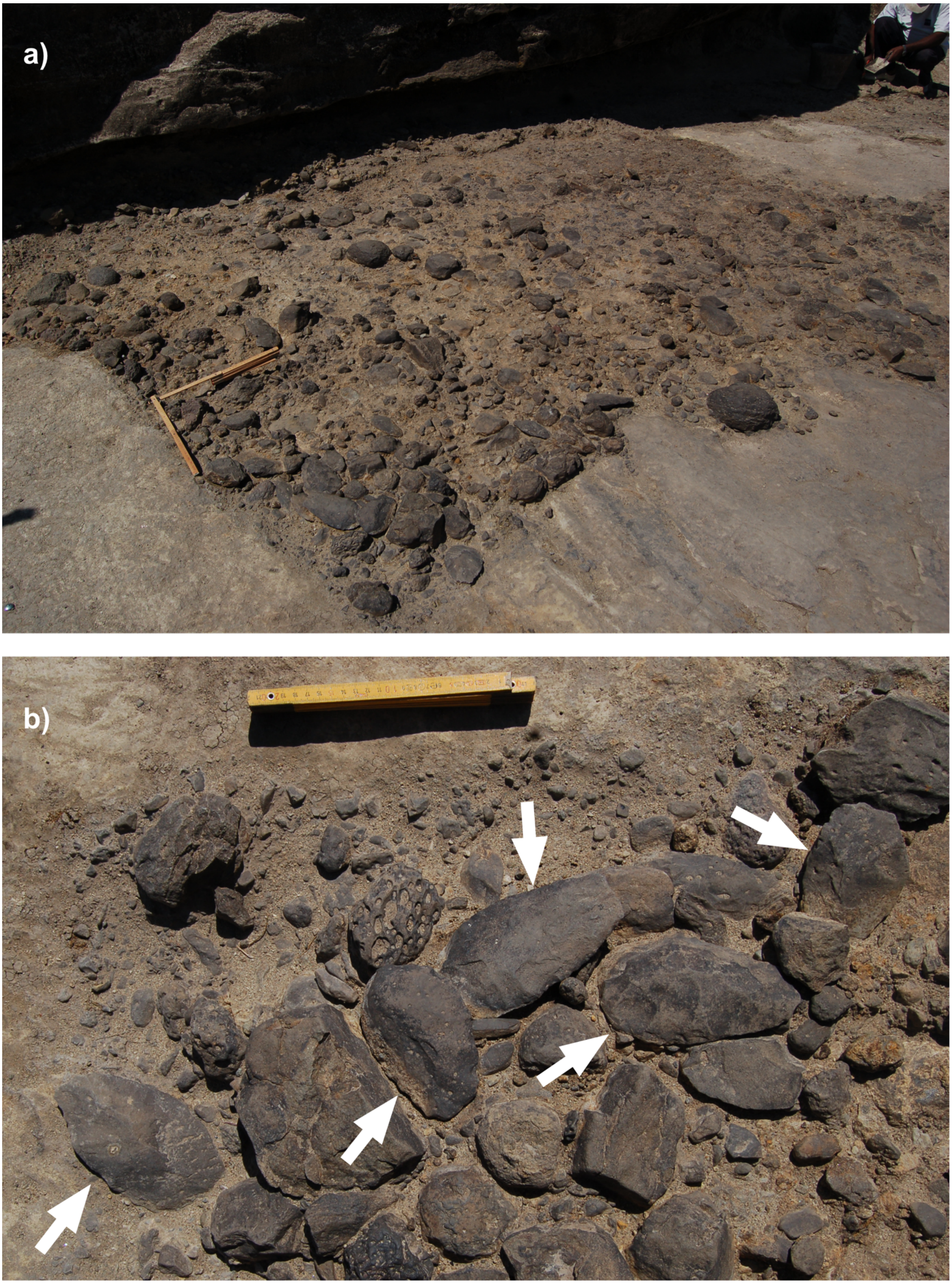

Fig. 4. Digital elevation model (a) and orthoimage (b) of the excavated surfaces (created in QGis software, using own data).

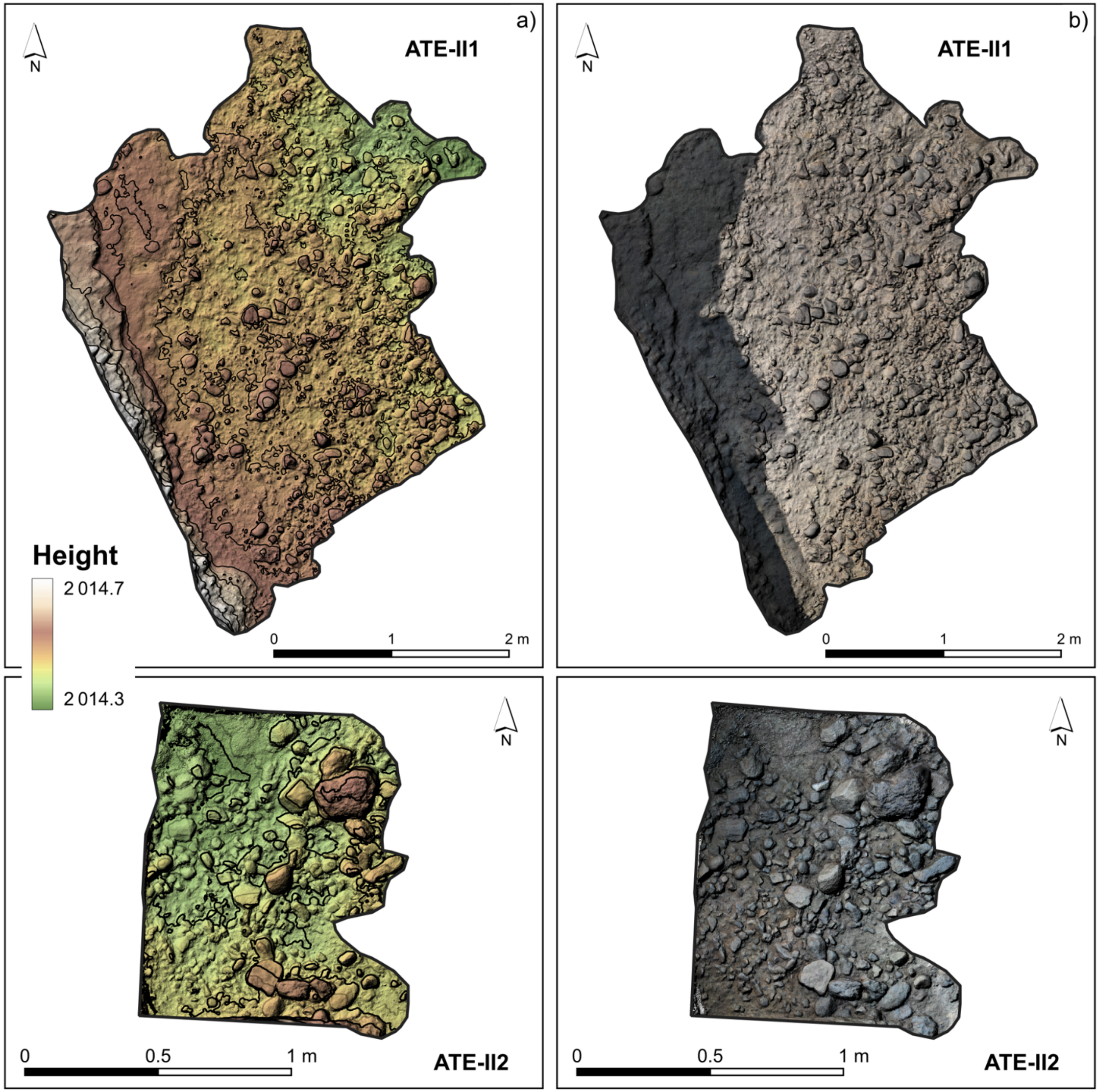

Fig. 5. 3D rendering of ATE-II, with detailed views of surfaces ATE-II1(a) and ATE-II2 (b) (created in Affinity Studio software, using own data).

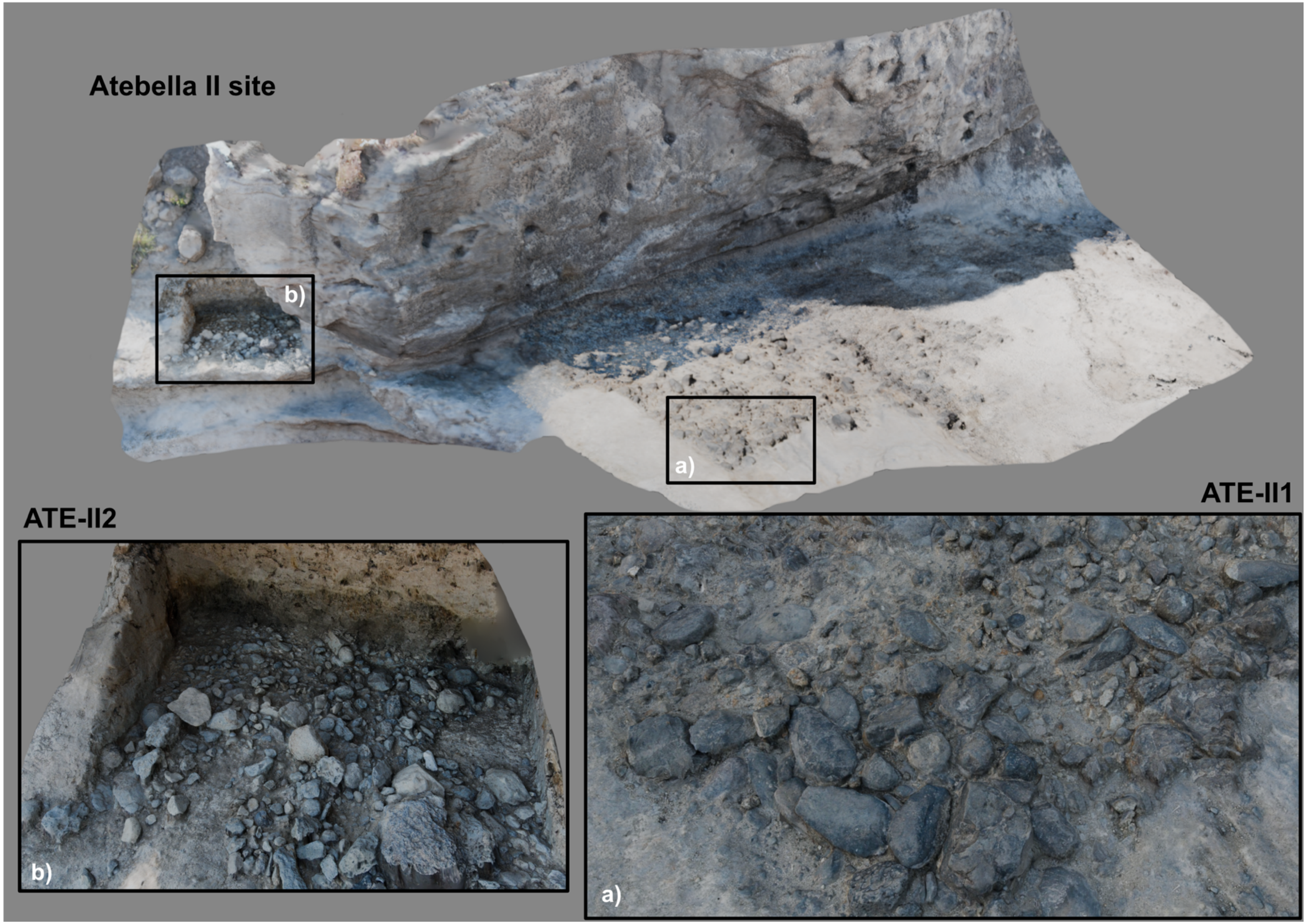

References

1. Chavaillon J, Berthelet A. The archaeological sites of Melka Kunture. In: Chavaillon J, Piperno M, editors. Studies on the Early Paleolithic site of Melka Kunture, Ethiopia. Roma: Istituto Italiano di Preistoria e Protostoria; 2004. p. 25–80.
