# Supplementary material for: Random walks in handaxe knapping before 1.2 million years ago in East Africa at Melka Kunture (Upper Awash, Ethiopia)
Source: PLoS One. 2026 Sep 24;21(9):e0357880. doi: 10.1371/journal.pone.0357880 (PMC13606939; doi:10.1371/journal.pone.0357880)
Supplement: Supplementary file 2 [file pone.0357880.s002.pdf]

S2 Appendix. The lithic tool assemblage from ATE-II

The stone tool assemblage identified from the two excavation sectors of the site comprises 47 pieces (**Table 1**). It is dominated by large cutting tools (LCTs) (n = 28, 59.6%), while the remaining technological categories are minimally represented. We retain here the 19 identified handaxes, i.e. 40.4% of the recovered artefacts.

Aside from the handaxes, which are the focus of this paper, we note a large number of cleavers-on-flake, including very distinctive specimens (**Fig. 1**), as well as volcanic spheres (1). Additionally, two hand-held cores were also identified. One exhibits a discoidal exploitation pattern and the other one, shows a Victoria-West reduction scheme. The assemblage furthermore includes a small cleaver-on-flake with the same characteristics. This predetermined flake removal method had previously been identified at the MS site, proving that the hominin groups of the region fully mastered the knapping procedure before 1.2 Ma (2).

The raw materials selected for toolmaking are exclusively of volcanic origin, in accordance with the regional geological features (3). The range of suitable rocks was wide, including coarser varieties like porphyritic and aphyric basalts and lavas, as well as obsidian. Accordingly, we distinguish between coarse volcanic rocks (CVR) and obsidian. In the ATE-II assemblage, CVR (n = 41, 87.2%) markedly predominate compared to obsidian (n = 6, 12.8%).

The density of artefacts is very low (6.7 pieces/m^2) when compared to other sites of the MK region in similar channel deposits (i.e., stone lines) (4–7). The low density of artefacts, as well as of faunal remains, along with the technological composition, rather than reflecting a definitively lower density relative to other sites, probably is the outcome of ongoing erosional processes. The higher density of piece at ATE-II2 (15 pieces/m^2), which is not an exposed surface, compared to just 5.3 pieces/m^2 at the exposed ATE-II1 deposit, further supports this hypothesis.

Table 1. Main technological categories identified within the ATE-II assemblage.

Technological category	*n*	%
Hammerstone/anvil	1	2.1
Tested pebble	1	2.1
Medium size flake (30–100 mm)	4	8.5
Large size flake (> 100 mm)	1	2.1
Flake fragment	1	2.1
Small-medium size core	2	4.3
Sub-spheroid	1	2.1
Spheres	1	2.1
Flake tool	3	6.4
Handaxe	19	40.4
Cleaver on flake	6	12.8
Large flake tool (> 100 mm)	3	6.4
Waste	4	8.5
**TOTAL**	**47**	

Fig. 1. Cleaver-on-flake on CVR from ATE-II.

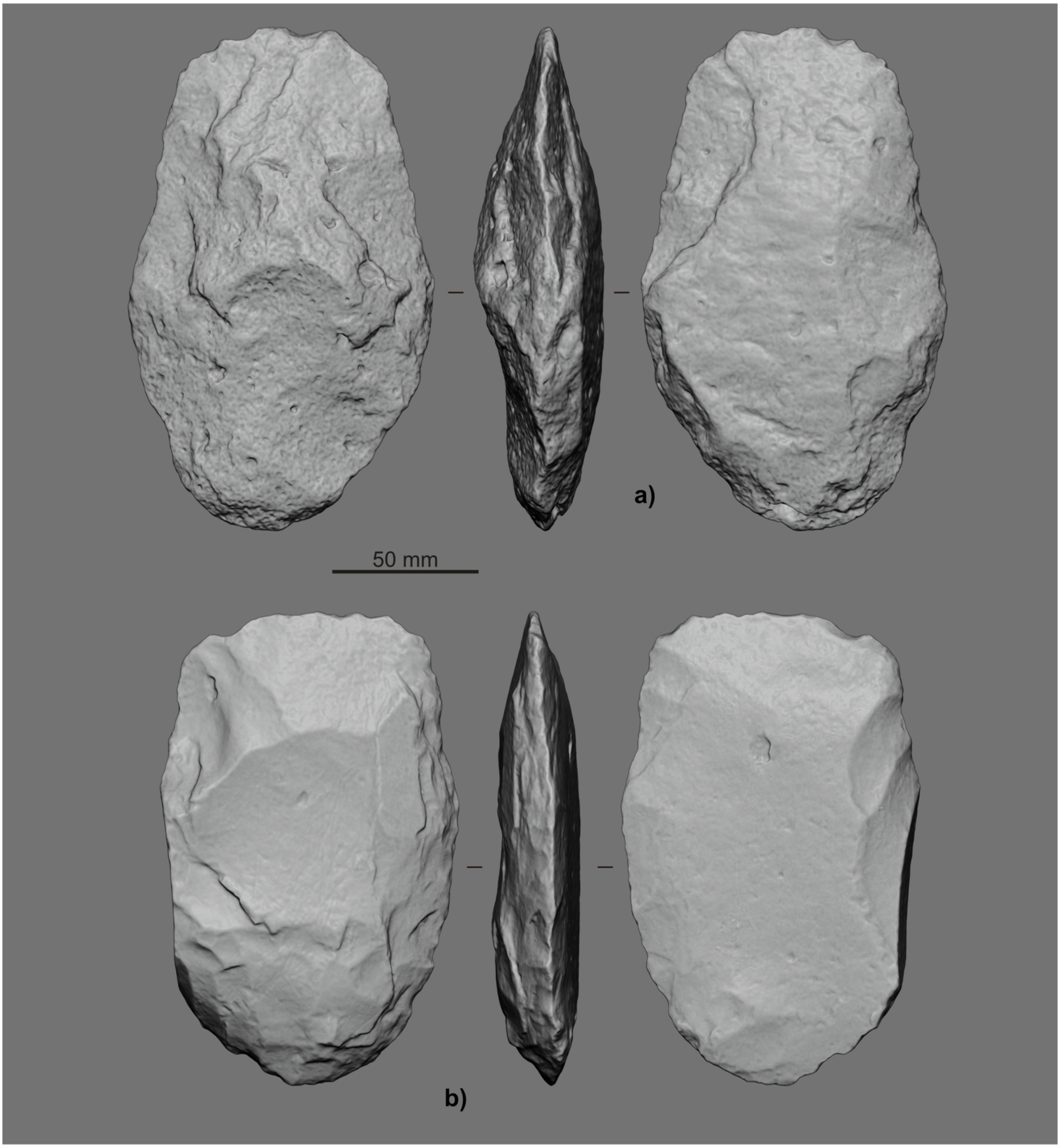

References

1. Mussi M. The volcanic rock spheres of Melka Kunture (Upper Awash, Ethiopia) at Gombore IB and later Acheulean sites. Quaternary International. 2025;721:109681. doi:10.1016/j.quaint.2025.109681

2. Méndez-Quintas E, Mussi M. Unravelling the development of large flake technology during the Early Acheulean: the evidence from Simbiro Gully at Melka Kunture (Upper Awash, Ethiopia). Journal of Paleolithic Archaeology. 2025;8(7). doi:10.1007/s41982-024-00207-w

3. Kieffer G, Raynal JP, Bardin G. Volcanic markers in coarse alluvium at Melka Kunture (Upper Awash, Ethiopia). In: Chavaillon J, Piperno M, editors. Studies on the Early Paleolithic site of Melka Kunture, Ethiopia. Roma: Istituto Italiano di Preistoria e Protostoria; 2004. p. 93–101.

4. Méndez-Quintas E, Melis RT, Altamura F, Di Bianco L, Panera J, Ruta G, et al. Archeology in "stone line" sedimentary environments. A methodological approach and results based on the Early and Middle Pleistocene sites at Melka Kunture (Upper Awash, Ethiopia). Journal of Mediterranean Earth Sciences. 2023;15. doi:10.13133/2280-6148/18153

5. Méndez-Quintas E, Melis RT, Bello-Alonso P. The early Acheulean site of Gombore I level B (Melka Kunture, Ethiopia): archaeological assemblage integrity and spatial distribution patterns. Quaternary International. 2025;723:109709. doi:10.1016/j.quaint.2025.109709

6. Méndez-Quintas E, Panera J, Altamura F, Di Bianco L, Melis RT, Piarulli F, et al. Gombore II (Melka Kunture, Ethiopia): A new approach to formation processes and spatial patterns of an Early Pleistocene Acheulean site. Journal of Archaeological Science. 2019;108:104975. doi:10.1016/j.jas.2019.104975

7. Melis RT, Mussi M. The paleolandscape of Melka Kunture (Ethiopia) at the time of the earliest human peopling. Palaeogeography, Palaeoclimatology, Palaeoecology. 2026;654:113520. doi:10.1016/j.palaeo.2025.113520
