# Supplementary material for: Random walks in handaxe knapping before 1.2 million years ago in East Africa at Melka Kunture (Upper Awash, Ethiopia)
Source: PLoS One. 2026 Sep 24;21(9):e0357880. doi: 10.1371/journal.pone.0357880 (PMC13606939; doi:10.1371/journal.pone.0357880)
Supplement: Supplementary file 3 [file pone.0357880.s003.pdf]

# S3 Appendix. Stone tool use-wear data

The lithic assemblage from ATE-II was sampled for a traceological analysis aimed at the study of use-wear traces at both macroscopic and microscopic levels. This work was carried out during the 2023 field season at the Ethiopian Heritage Authority (EHA) laboratory in Addis Ababa.

All available artefacts housed in the museum were examined, totalling 51 tools. They exhibit notable variability in raw materials, even if always within the category of volcanic rocks, including obsidian, undetermined volcanic rocks, ignimbrites, lavas, and porphyritic basalts. Volcanic spheres and hammerstones were excluded due to the lack of a reference collection regarding the formation and development of use-wear traces.

The macroscopic examination of the tools revealed significant preservation issues. None of the pieces displayed optimal preservation conditions for a traceological analysis. The alterations of each artefact were recorded together with a detailed description. Overall, the following alterations were identified: sediment/crust adhesion, rounding and abrasion. Sediment and crust adhesion was the least frequent, observed in 25.49% of the lithic material (**Table 1**). In contrast, both rounding and abrasion were documented in 98.03% of the tools (**Table 1**).

The poor state of preservation of the ATE-II lithic assemblage is not entirely unexpected, as it can be attributed to two key factors: the site environment (see the geo-stratigraphic section) and the volcanic nature of the raw materials. As noted in previous traceological studies (1,2), the conservation of lithic industries on volcanic supports is often poor despite rock hardness. Erosion (i.e. material loss from the surface) is the outcome of abrasion and is frequently noted. This issue appears to be related to knapping properties, as volcanic rocks, although durable, tend to suffer from significant conservation problems, mainly material loss (erosion) because of abrasion (**Fig. 1**).

Accordingly, almost all the ATE-II lithic collection shows surface preservation issues which prevent use-wear analysis at microscopic level and the study of polish, striations, linear components, etc. At macroscopic level, some artefacts preserve identifiable original features -scars- (**Fig. 1b**), which are possibly related to use; however, these are scarce and difficult to interpret due again to erosion. Moreover, the lack of a dedicated reference collection supporting this type of analysis, alongside the absence of microscopic traces allowing to establish correlation patterns, is currently a major limitation to a reliable macroscopic analysis.

**Table 1.** ATE-II lithic industry: alteration data.

| Session | Box | Level | Piece | Raw material | Alterations | | |
|---|---|---|---|---|---|---|---|
| | | | | | Abrasion | Adhesion | Rounding |
| MK 2011 | AA3 | ATE II-1 | A 172 | Obsidian | x | x | x |
| | | ATE II-2 | A 202 | Obsidian | x | x | x |
| | | ATE II-2 | A 140 | Volcanic rock | x | x | x |
| | | ATE II-2 | A 5 | Obsidian | x | x | x |
| | | ATE II-2 | A 162 | Obsidian | x | x | x |
| | | ¿? | A 113 | Obsidian | x | x | x |
| | | ATE II-2 | A 84 | Ignimbrite | x | x | x |
| | | ¿? | A74 | Ignimbrite | | x | |
| | | ATE II-2 | A 56 | Lava | x | x | x |
| | | ATE II-2 | A 10 | Porphiric basalt | x | x | x |
| | | ATE II-2 | A 131 | Volcanic rock | x | | x |
| | | ATE II-2 | A 252 | Porphiric basalt | x | | x |
| | | ATE II-2 | A 4 | Obsidian | x | x | x |
| | | ATE II-2 | A 76 | Volcanic rock | x | | x |
| | | ATE II-2 | A 251 | Volcanic rock | x | | x |
| | | ATE II-2 | A 129 | Porphiric basalt | x | | x |
| | | ATE II-2? | A 183 | Volcanic rock | x | | x |
| | | ATE II-1 | A 131 | Volcanic rock | x | | x |
| | | ATE II-2 | A 217 | Volcanic rock | x | | x |
| | | ATE II-2 | A 130 | Volcanic rock | x | | x |
| | | ATE II-1 | A74 | Volcanic rock | x | | x |
| | | ATE II-2 | A 70 | Volcanic rock | x | | x |
| | | ATE II-1 | A 135 | Volcanic rock | x | | x |
| | | ATE II-1 | A 227 | Volcanic rock | x | | x |
| | | ATE II-1 | A 27 | Volcanic rock | x | | x |
| | | ATE II-1 | A 249 | Volcanic rock | x | | x |
| | | ATE II-2 | A 121 | Volcanic rock | x | | x |
| | AA4 | ATE II-1 | A 82 | Volcanic rock | x | x | x |
| | | ATE II-1 | A 80 | Volcanic rock | x | | x |
| | | ATE II-1 | A 5 | Volcanic rock | x | | x |
| | | ATE II-1 | A 78 | Obsidian | x | x | x |

| | | | | | | | |
|---|---|---|---|---|---|---|---|
| | | ATE II-2 | A 2 | Volcanic rock | x | | x |
| | | ATE II-1 | A 4 | Volcanic rock | x | | x |
| | | ATE II-1 | A 46 | Volcanic rock | x | | x |
| | | ATE II-1 | A 8 | Volcanic rock | x | | x |
| | | ATE II-1 | A 25 | Volcanic rock | x | | x |
| | | ATE II-1 | A 79 | Volcanic rock | x | | x |
| | | ATE II-1 | A 10 | Volcanic rock | x | | x |
| | | ATE II-1 | A 36 | Volcanic rock | x | | x |
| | | ATE II-1 | A 19 | Volcanic rock | x | | x |
| | | ATE II-1 | A 6 | Volcanic rock | x | | x |
| | | ATE II-1 | A 95 | Volcanic rock | x | | x |
| | | ATE II-1 | A 1 | Volcanic rock | x | | x |
| | | ATE II-1 | A 44 | Volcanic rock | x | | x |
| | AC5 | ATE II-1 | A141 | Lava | x | | x |
| | | ATE II-1 | A 187 | Volcanic rock | x | | x |
| | | ATE II-1 | A 118 | Volcanic rock | x | | x |
| | | ATE II-1 | A 45 | Volcanic rock | x | | x |
| | | ATE II-1 | A 20 | Volcanic rock | x | | x |
| | | ATE II-1 | A 24 | Volcanic rock | x | | x |
| | | ATE II-1 | A 248 | Volcanic rock | x | | x |

**Fig. 1.** Handaxe ATE II-1. A95. Volcanic rock exhibiting conservation issues related to abrasion, rounding, and material loss: 1a) surface erosion and rounded edges, and 1b) surface abrasion and rounding. Photographs copyright archive of Italo-Spanish Archaeological Mission at Melka Kunture and Balchit.

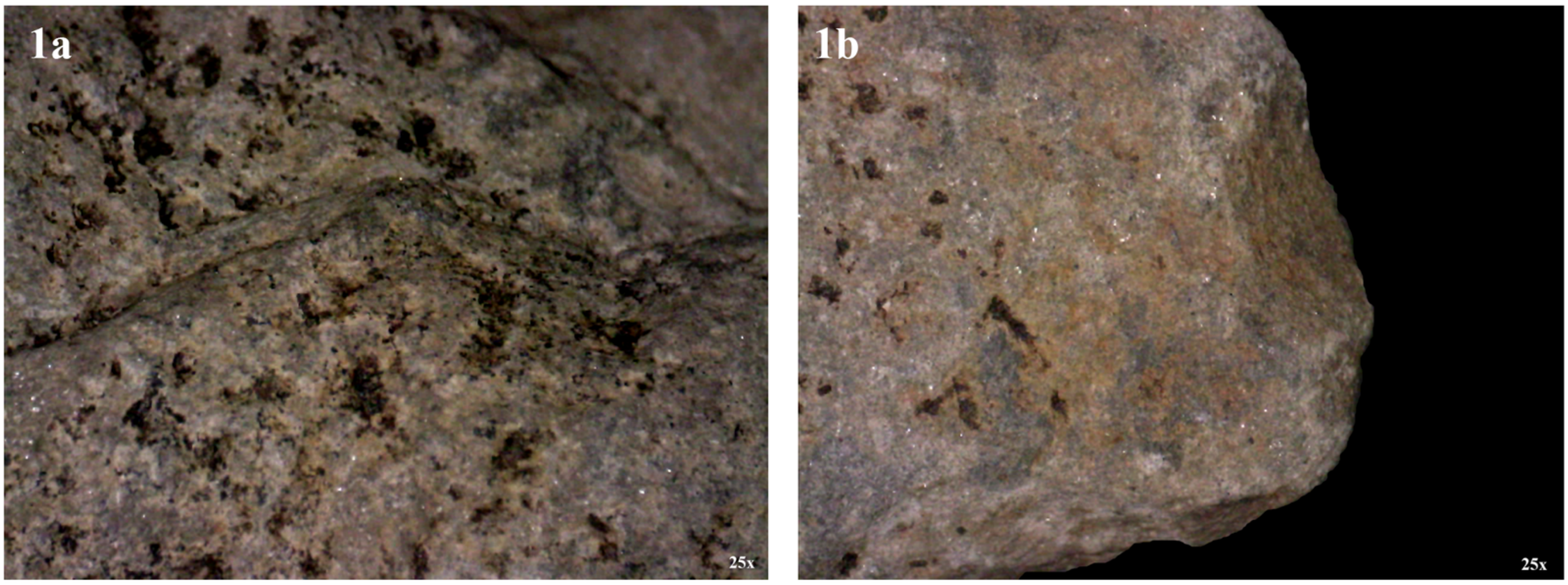

**Supplementary references**


1. Bello-Alonso P, Rios-Garaizar J, Panera J, Rubio-Jara S, Pérez-González A, Rojas R, et al. The first comprehensive micro use-wear analysis of an early Acheulean assemblage (Thiongo Korongo, Olduvai Gorge, Tanzania). Quaternary Science Reviews. 2021 Jul 1;263:106980. doi:https://doi.org/10.1016/j.quascirev.2021.106980

2. Bello-Alonso P. Estudio traceológico de la industria lítica achelense de Thiongo Korongo (Garganta de Olduvai, Tanzania): aproximación experimental y arqueológica a la gestión económica a partir del análisis de filos. Oxford: BAR Publishing; 2022. 180 p. (BAR International Series; no. 3073).
