# Supplementary material for: Random walks in handaxe knapping before 1.2 million years ago in East Africa at Melka Kunture (Upper Awash, Ethiopia)
Source: PLoS One. 2026 Sep 24;21(9):e0357880. doi: 10.1371/journal.pone.0357880 (PMC13606939; doi:10.1371/journal.pone.0357880)
Supplement: Supplementary file 4 [file pone.0357880.s004.pdf]

S4 Appendix. Photogrammetric recording at ATE II

The objective of the work is the creation of a georeferenced 3D model of a dry portion of the Atebella riverbed close to the archaeological site of Melka Kunture. The area of interest is approximately 40 m² at the foot of a rock front, made-up of ignimbrite material, more than 3 m high and facing E-NE.

Survey

The photogrammetric survey was carried out in November 2011 using a Nikon D80 digital camera with a 18 mm focal length and a speed of ISO 640. The shutter speeds and apertures varied during image acquisition due to different lighting conditions of the vertical walls compared to the horizontal surfaces. While for the former it was possible to take frontal photos from the ground, for the latter it was necessary to prepare a wooden scaffold to support a pulley for taking nadiral photos (**Fig 1**). A total of 556 photographs were acquired. A second survey was carried out with the same method on a small excavation site adjacent to the study area. In this case, 27 photos were acquired.

Fig 1. Wooden scaffold built *on site* for nadiral photos acquisition. Photograph copyright archive of Italo-Spanish Archaeological Mission at Melka Kunture and Balchit.

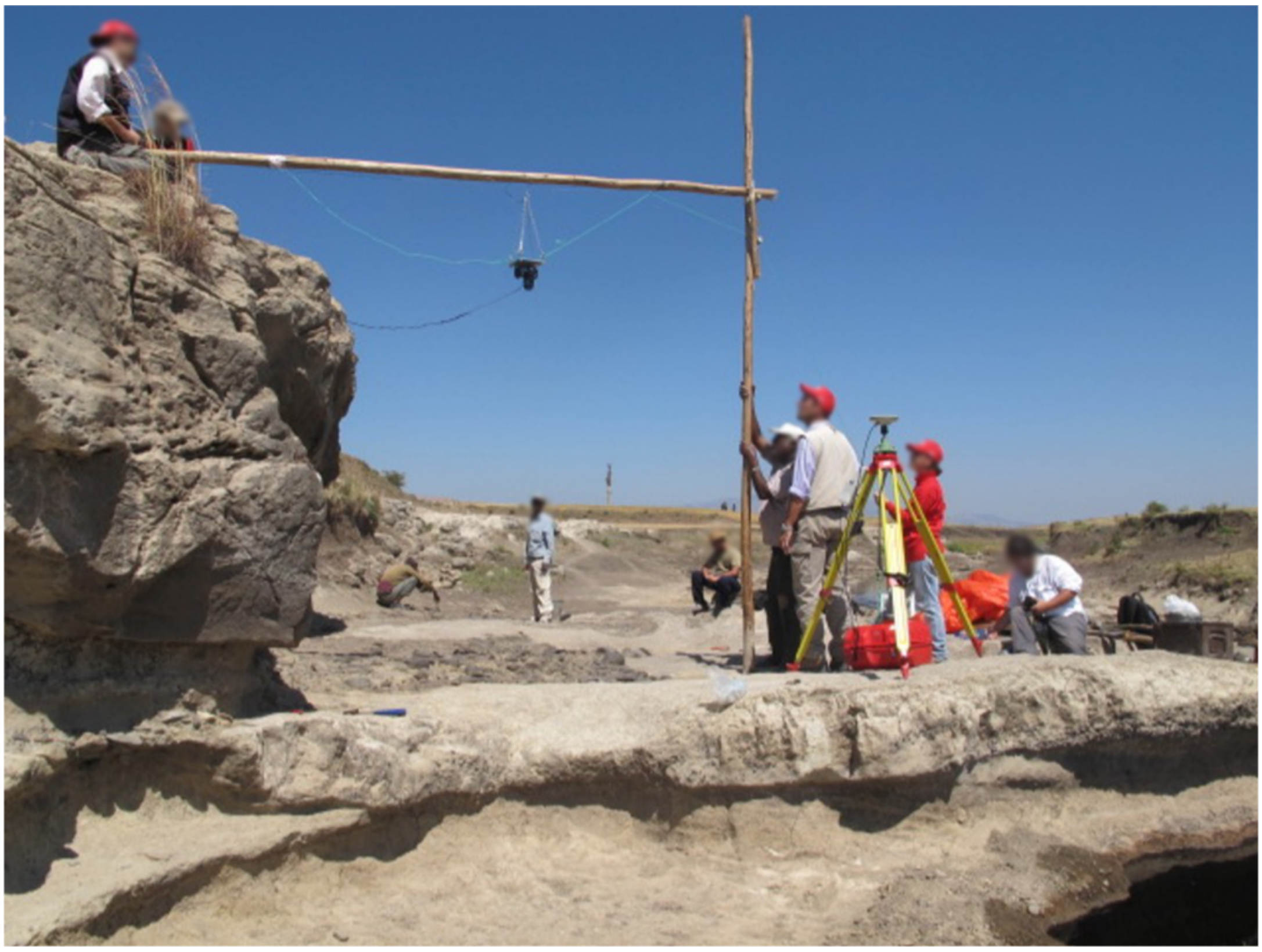

To frame the model in the UTM-WGS84 geodetic reference system (Spheroid Clarke 1880, Datum Adindan, Zone 37 N), a geodetic-type Leica™ 1200 GNSS (Global Navigation Satellite System) instrumentation, operating in static mode with acquisition times exceeding 3 hours, was used. Starting from a point of known coordinates located at Melka Kunture archaeological open-air museum, which was measured in previous survey campaigns with respect to trigonometric points made available by the Mapping Agency of Addis Ababa, the absolute coordinates of 4 measurement GNSS points were acquired. The latter were used as reference point for a

topographic survey executed by means of a Total Station (TS – Leica™ TCRP 1203+R1000 model) which was used to measure the Ground Control Points (GCPs) necessary for the absolute orientation of all the images, both nadiral and frontal with respect to the rock face. A total of 114 GCPs was measured with the aid of the TS.

Data processing

The processing of the images acquired by the photogrammetric survey was performed through Agisoft™ Metashape Professional software. Inside the software workspace, the photos were inserted into 4 different chunks, distributed according to the direction of acquisition and the TS bases. Fig 9 shows a schematic representation of the different chunks.

Fig 2. Schematic representation of the 4 chunks positioning (created in Agisoft™ Metashape Professional software, using own data). Black and blue boxes indicate the extent of nadiral acquisition photos while red and yellow boxes indicate the extent of images taken frontally with respect to the rock walls.

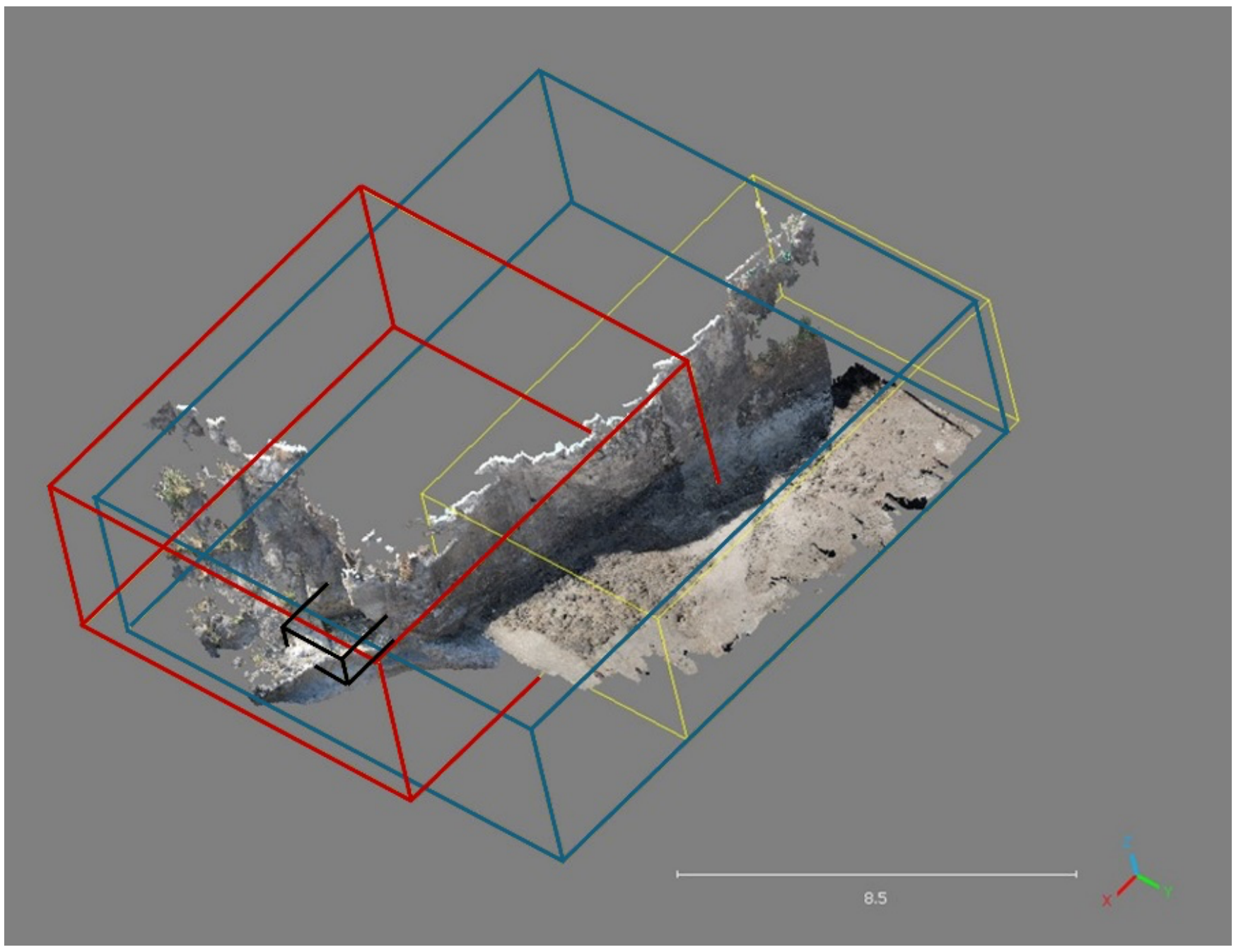

The 556 photographs of ATE-II1 underwent software quality control. Among them, 159 returned a rating lower than 0.6 which, on a scale from 0 to 1, was considered as the lower quality limit to consider an image acceptable: sometimes photos were found too bright, too dark or not focused. The remaining 397 photos were aligned in their respective chunks, according to the parameters shown in Fig 10, and the related sparse point clouds produced.

Fig 3. Alignment parameters used within Agisoft™ Metashape software.

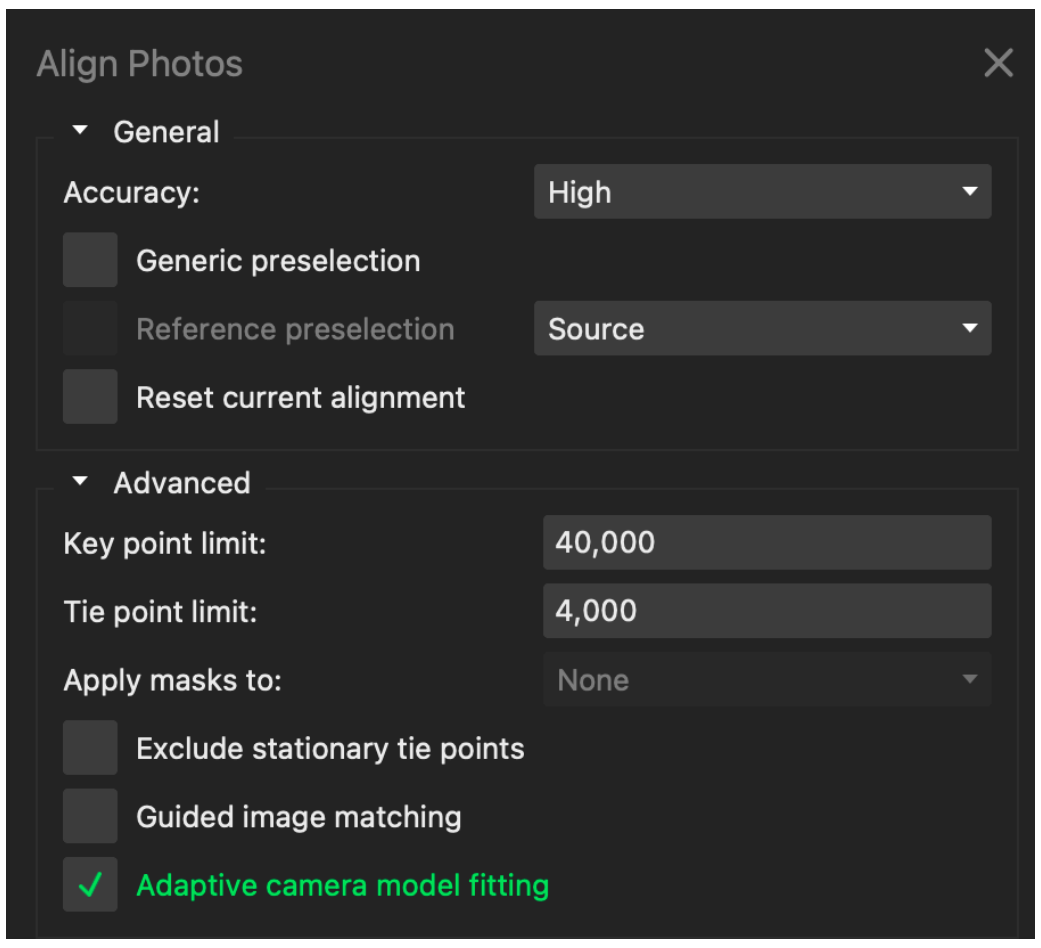

Subsequently, the collimation of the GCPs was carried out on the images and the respective coordinates were imported. Fig 11 shows, as an example, the positioning and relative errors (magnified by 20 in size) of 45 GCPs and 6 check points (CPs) used to georeference the blue chunk of Fig 11. Following this operation, the process of optimization and roto-translation of the blocks into the chosen reference system started.

Fig 4. Placement of GCPs and CPs (created in Agisoft™ Metashape Professional software, using own data). The point positioning errors are represented by an error ellipse with a scaling factor of x20.

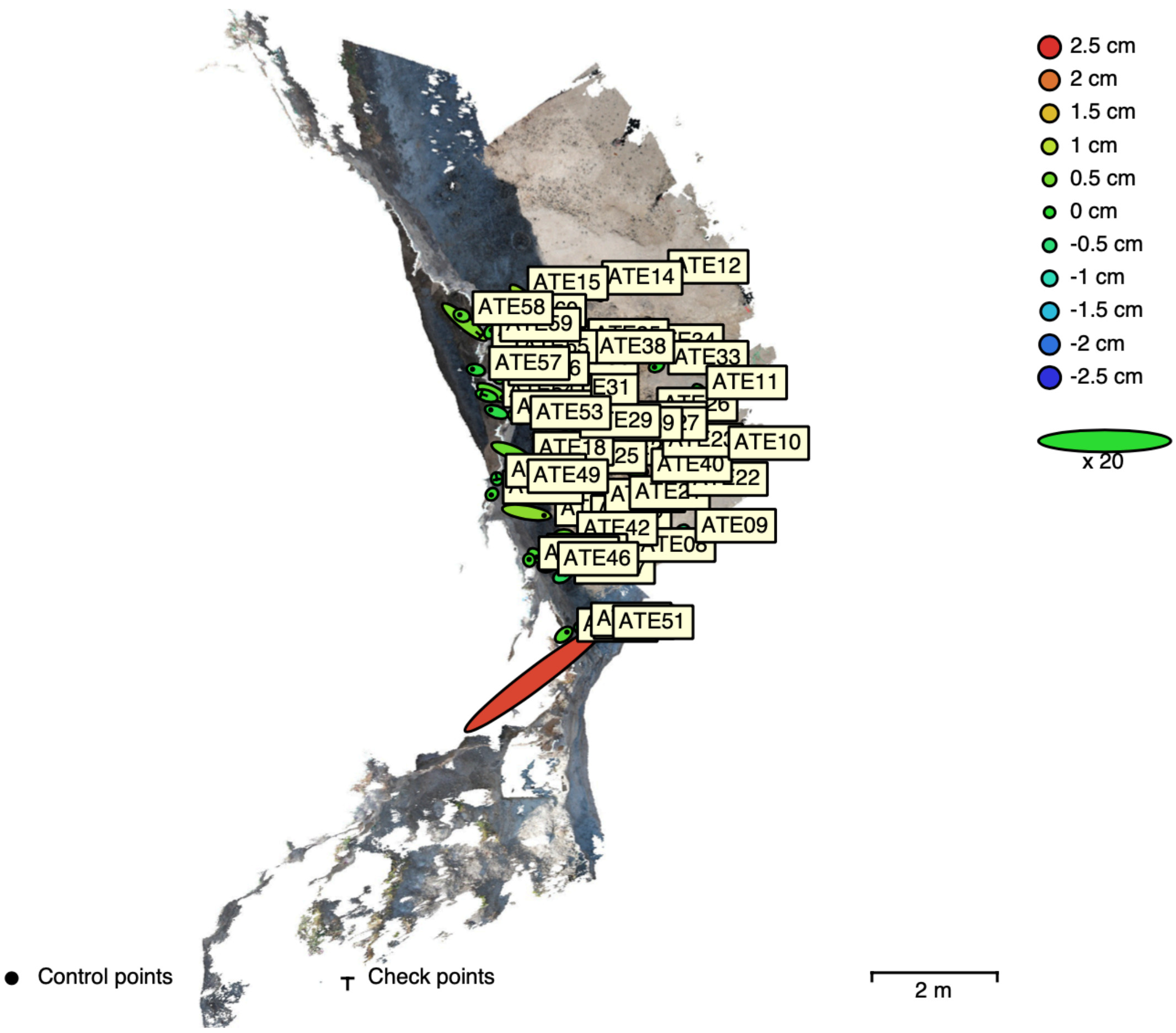

Table 1. RMSEs of GCPs for the 4 chunks.

Count	X error (cm)	Y error (cm)	Z error (cm)	XY error (cm)	Total (cm)
76	0.699383	0.607642	0.40122	0.92648	1.00963

Table 2. RMSEs of CPs for the 4 chunks.

Count	X error (cm)	Y error (cm)	Z error (cm)	XY error (cm)	Total (cm)
16	2.8605	2.01425	0.683204	3.49852	3.56461

After the georeferencing of the sparse point clouds, dense clouds of three dimensional points were processed for the four chunks by using the parameters shown in Fig 5.

Fig 5. Parameters used in Agisoft™ Metashape software for dense point clouds building.

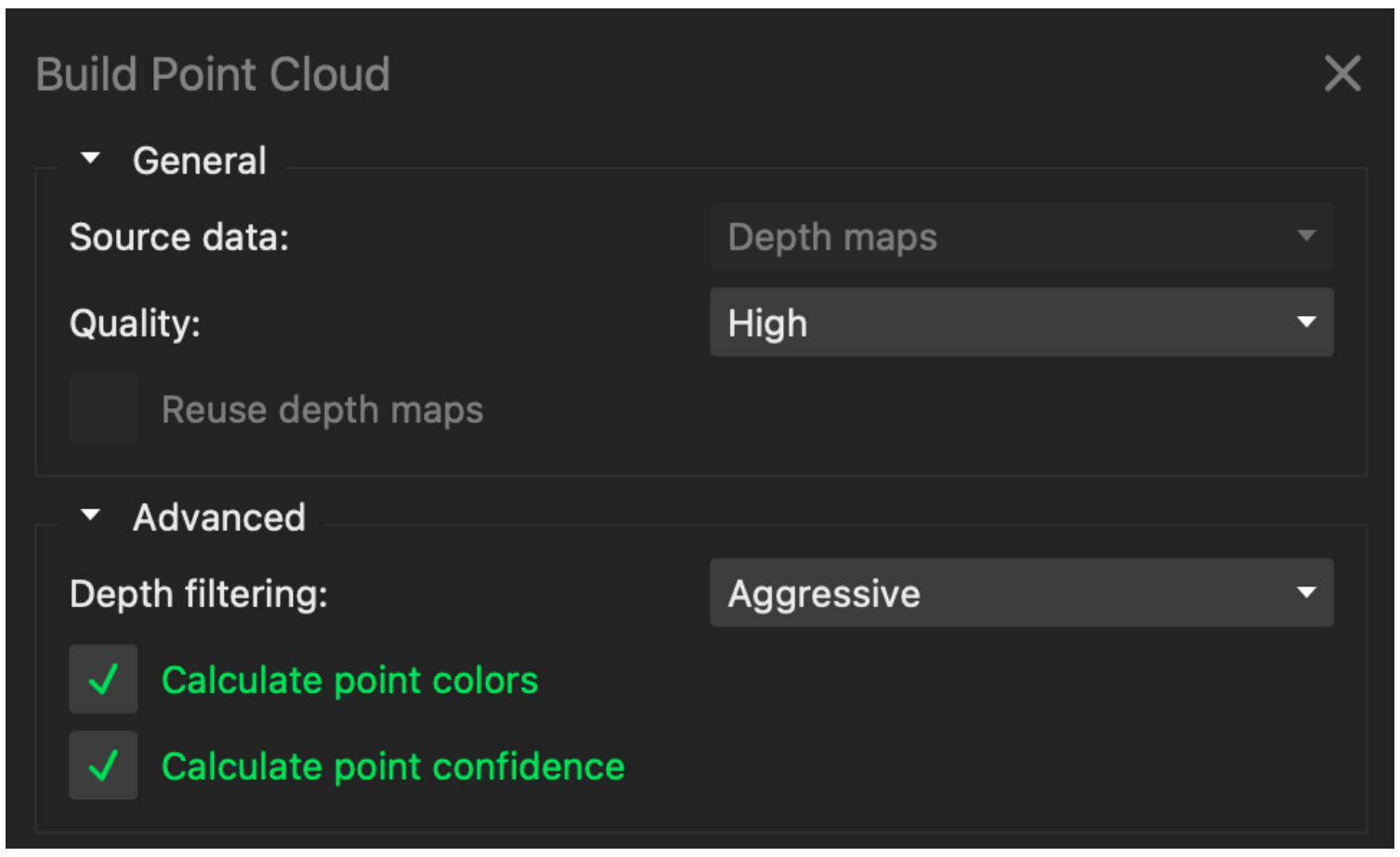

The four georeferenced point clouds were finally merged, and a final dense point cloud made of approximately 51 million points was generated. This cloud was subsequently decimated by setting a minimum distance between the points equal to 0.6 cm, bringing the count of 3D points to approximately 15 million. From both clouds, the photos of the survey were used to produce two textured meshes consisting of 30 million and 4 million faces, respectively. Finally, from the nadiral photos, the orthomosaic of the site was created for the ground level and the excavation trial at a spatial resolution of 0.9 and 0.3 mm, respectively.
