# Supplementary material for: Random walks in handaxe knapping before 1.2 million years ago in East Africa at Melka Kunture (Upper Awash, Ethiopia)
Source: PLoS One. 2026 Sep 24;21(9):e0357880. doi: 10.1371/journal.pone.0357880 (PMC13606939; doi:10.1371/journal.pone.0357880)
Supplement: Supplementary file 5 [file pone.0357880.s005.pdf]

S5 Appendix. History of research at Simbiro-Monumental Section

The Monumental Section of the Simbiro gully (MS) was discovered in the 1960s but was only investigated in the final days of 1973, when a large *Pelorovis oldowayensis* skull, still partially embedded, came to light due to heavy rains and erosion. Proper excavations were conducted during three brief field seasons in 1974, 1975, and 1976 (1–3). From 1974 to 1976, Ouardya Oussedik directed the field research (2) (**Fig. 1**). Then, in 2004, when Marcello Piperno was the director of the Italian Archaeological Mission, another small test was dug in level C, 8 m south of Oussedik's trench in that same level. Overall, approximately 18.7 m^2 of the MS were excavated in the last 50 years, with 4 main archaeological levels: from top to bottom, levels A, B, C, E (**Fig. 2**).

Fig. 1. Surveys of MS level B (a), and detail of the exposed surface (b) during the 1974 field season. Photographs copyright archive of Italo-Spanish Archaeological Mission at Melka Kunture and Balchit.

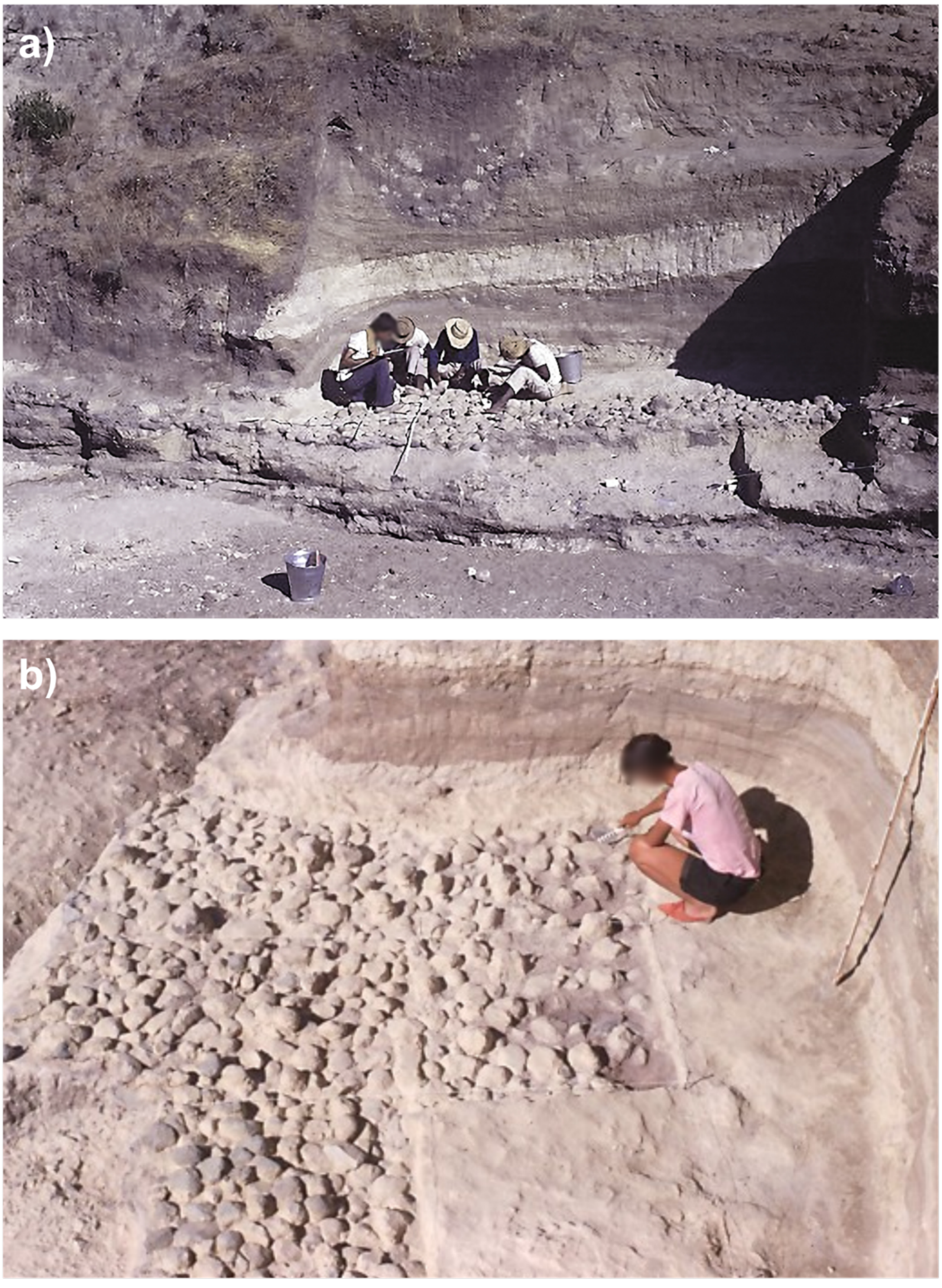

Fig. 2. Map of the Simbiro gully (MS area) showing the excavations, and planimetries of excavated Levels B and C (b-c) (created in QGis software, using own data).

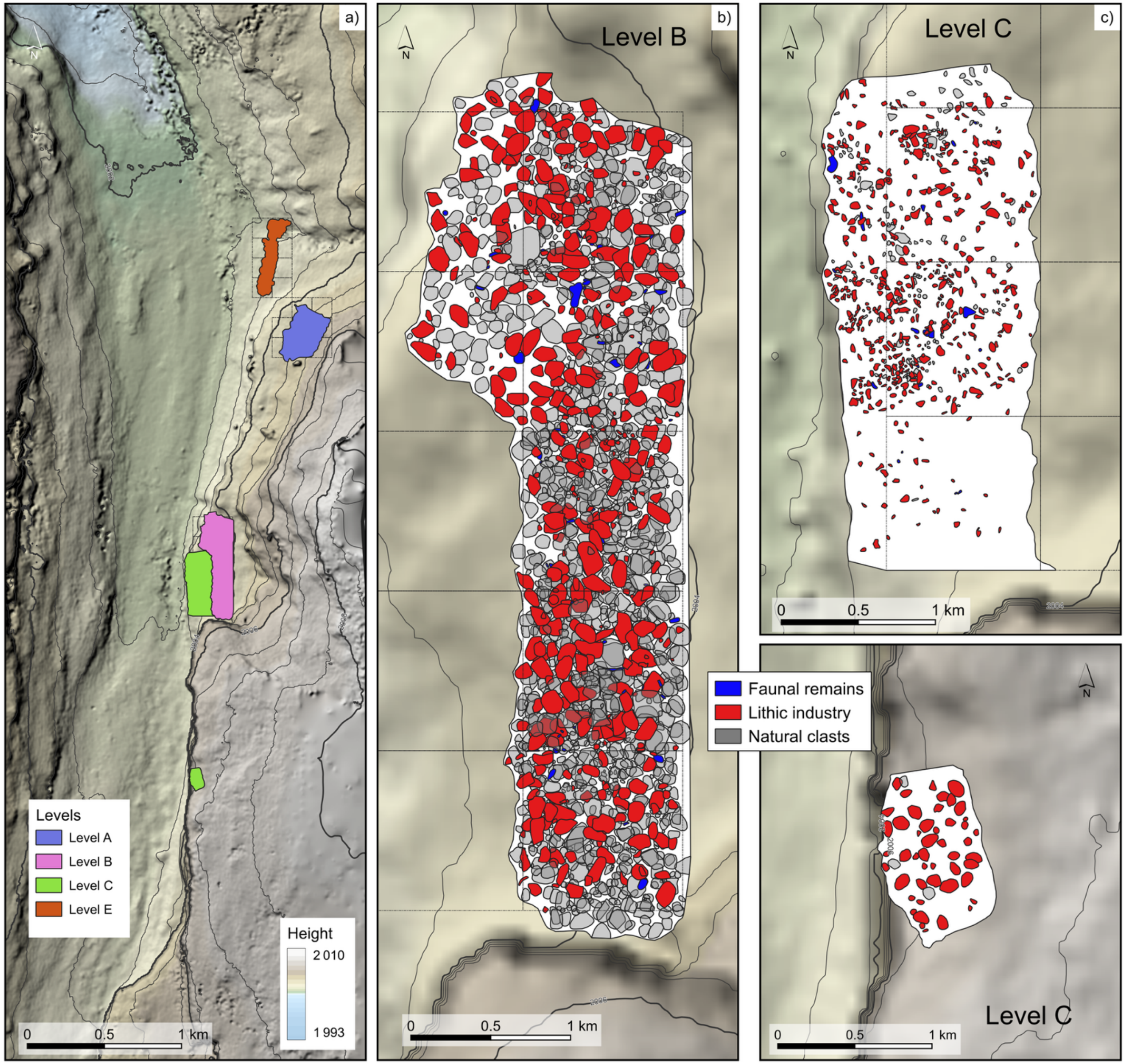

References

1. Chavaillon J, Berthelet A. The archaeological sites of Melka Kunture. In: Chavaillon J, Piperno M, editors. Studies on the Early Paleolithic site of Melka Kunture, Ethiopia. Roma: Istituto Italiano di Preistoria e Protostoria; 2004. p. 25–80.

2. Oussedik O. Site de Simburro III à Melka-Kunturé. L'Ethiopie avant l'Histoire. 1976;1:27–33.

3. Mussi M, Méndez-Quintas E, Barboni D, Bonnefille R, Briatico G, Geraads D, et al. A surge in obsidian exploitation and handaxe manufacture more than 1.2 million years ago at Simbiro III (Melka Kunture, Upper Awash, Ethiopia). Nature Ecology & Evolution. 2023 Mar;7(3):337–46. doi:10.1038/s41559-022-01970-1
