# Supplementary material for: Random walks in handaxe knapping before 1.2 million years ago in East Africa at Melka Kunture (Upper Awash, Ethiopia)
Source: PLoS One. 2026 Sep 24;21(9):e0357880. doi: 10.1371/journal.pone.0357880 (PMC13606939; doi:10.1371/journal.pone.0357880)
Supplement: Supplementary file 6 [file pone.0357880.s006.pdf]

S6 Appendix. The lithic tool assemblage from MS at Simbiro

A total of 18.7 m² was excavated across the site, with unequal distribution among levels (1). The assemblages from the various excavations total 1,556 artefacts, with uneven distribution across each level (1). Overall, the densities are high, far exceeding those observed at ATE-II: level B = 96.4 pieces/m^2 and level C = 120.4 pieces/m^2.

The artefacts are knapped mainly on CVR (mainly aphyric and porphyric lavas or basalts) and obsidian, with a large variability in raw material management over time. The proportion of CVR to obsidian is balanced in level A (52.1% and 47.9%) and level E (61.4% and 38.6%). In level C, obsidian use is nearly exclusive (99.5%), while in level B, CVR dominate (81.7%). **Table 1** summarize the data on the different technological categories. The most numerous assemblages, those of levels B and C, are dominated by flake products, but all levels show a large proportion of LCTs, i.e. handaxes, cleavers-on-flake (**Fig. 1**), and large flakes.

Table 1. Main technological categories identified within the MS assemblages

**Technological category**	**Level B**		**Level C**	
	n	*%*	*n*	*%*
Tested pebble	5	0.8	0	0.0
Hammerstone	2	0.3	2	0.3
Spheroid	1	0.2	0	0.0
Small size (<30 mm) flake	20	3.1	112	19.4
Medium size (30-100 mm) flake	141	22.2	307	53.1
Large size (>100 mm) flake	60	9.4	2	0.3
Flake fragment	0	0.0	20	3.5
Small-medium size core	43	6.8	6	1.0
Flake tool	80	12.6	33	5.7
Flake from tool resharpening	7	1.1	30	5.2
Handaxe	71	11.2	32	5.5
Cleaver-on-flake	93	14.6	0	0.0
Trihedral pick	0	0.0	0	0.0
Large flake tool (>100 mm)	74	11.6	4	0.7
Flakes from LCT reduction	7	1.1	5	0.9
LCT fragment	4	0.6	4	0.7
Pebble tool	6	0.9	0	0.0
Waste	22	3.5	21	3.6
**TOTAL**	**636**		**578**	

Fig. 1. Cleavers-on-flake from MS. (a) Cleaver on non-cortical flake, on Kombewa flake (b), and on predetermined flake (c). a-b are on CVR, and c on obsidian.

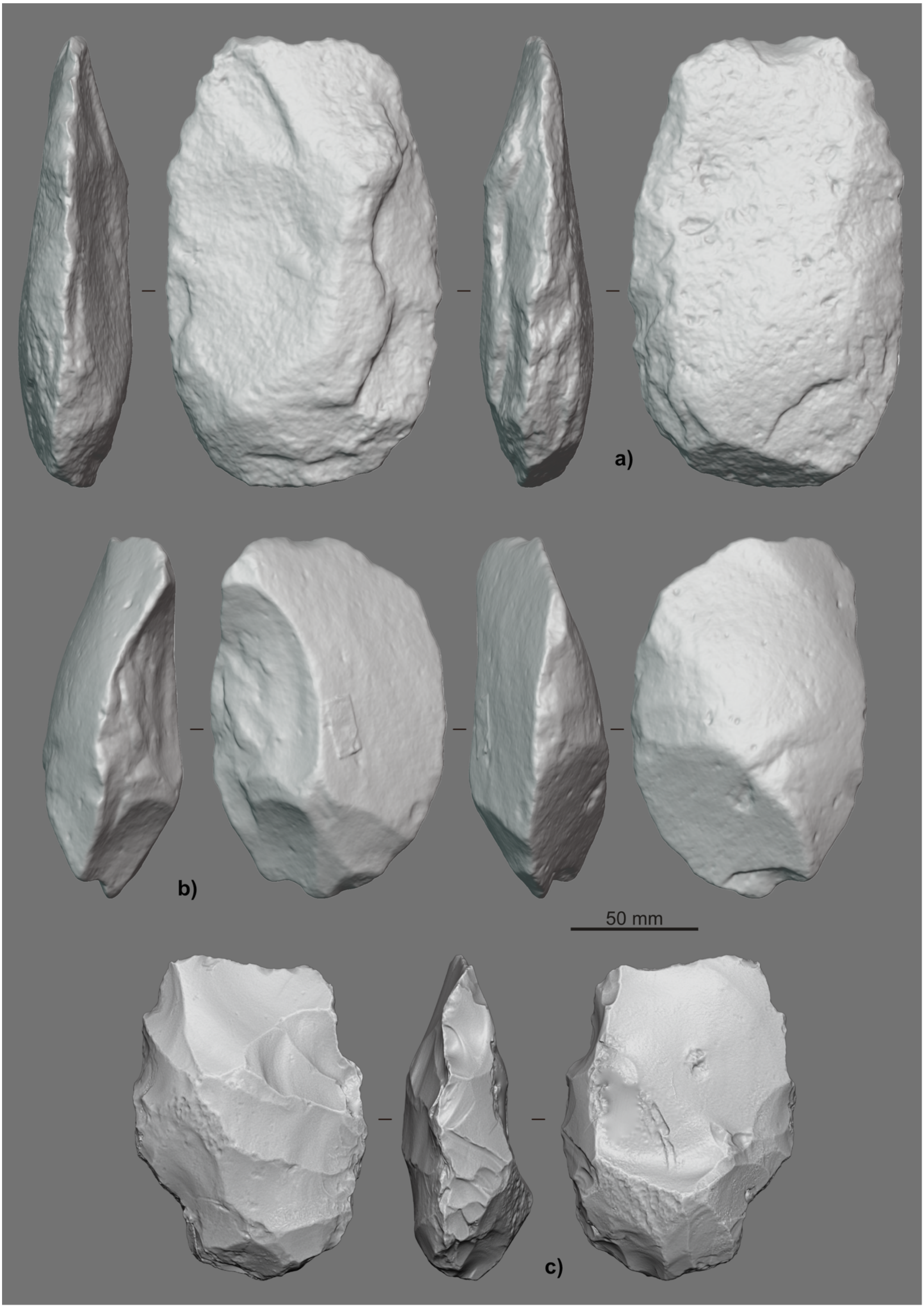

References

1. Mussi M, Méndez-Quintas E, Barboni D, Bonnefille R, Briatico G, Geraads D, et al. A surge in obsidian exploitation and handaxe manufacture more than 1.2 million years ago at Simbiro III (Melka Kunture, Upper Awash, Ethiopia). Nature Ecology & Evolution. 2023 Mar;7(3):337–46. doi:10.1038/s41559-022-01970-1
