# Supplementary material for: Random walks in handaxe knapping before 1.2 million years ago in East Africa at Melka Kunture (Upper Awash, Ethiopia)
Source: PLoS One. 2026 Sep 24;21(9):e0357880. doi: 10.1371/journal.pone.0357880 (PMC13606939; doi:10.1371/journal.pone.0357880)

Fig. 1. Handaxes from ATE-II, all of them on CVR (created in Affinity Studio software, using own data).

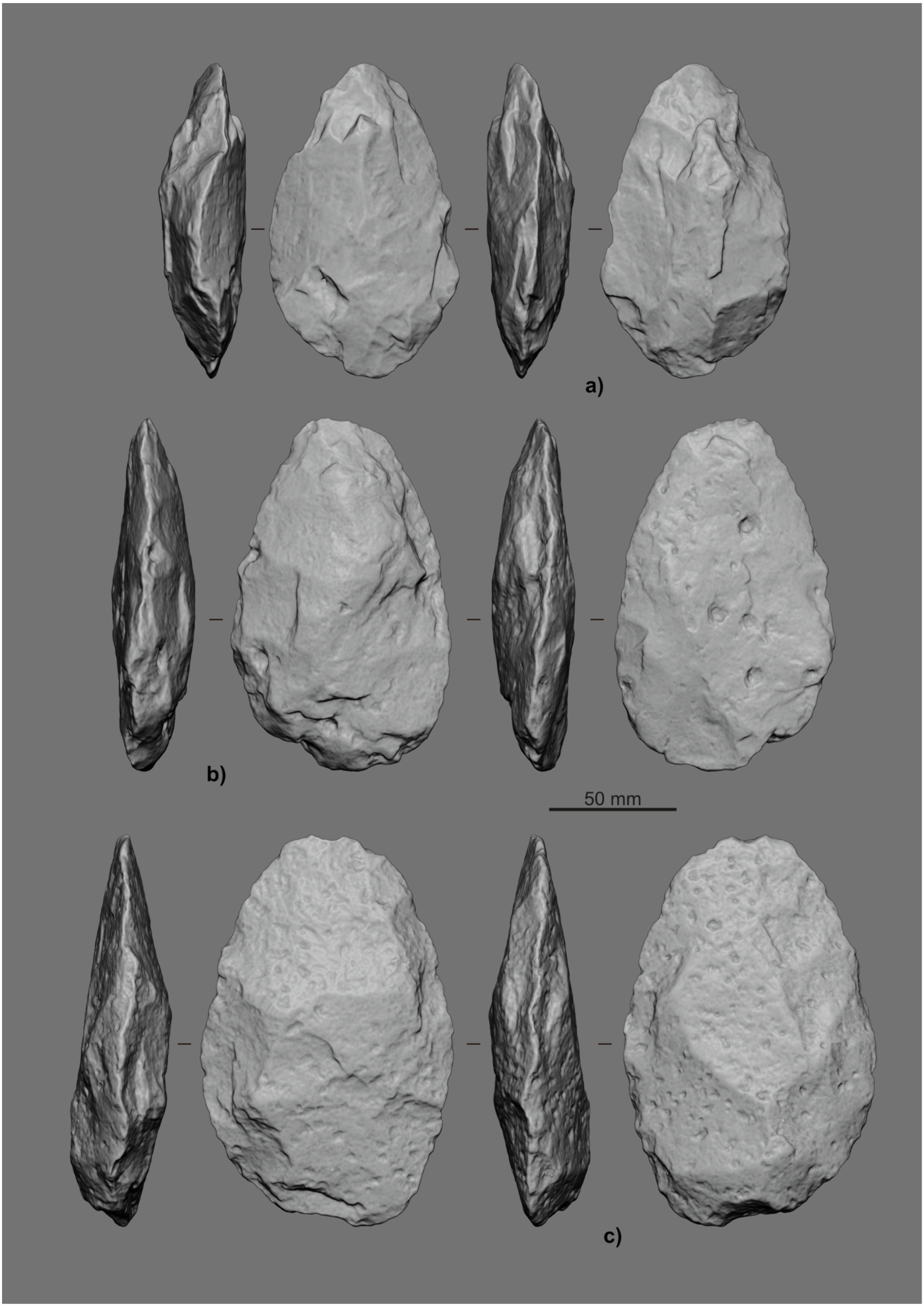

Fig. 2. Handaxes from ATE-II. a-b on CVR and c on obsidian (created in Affinity Studio software, using own data).

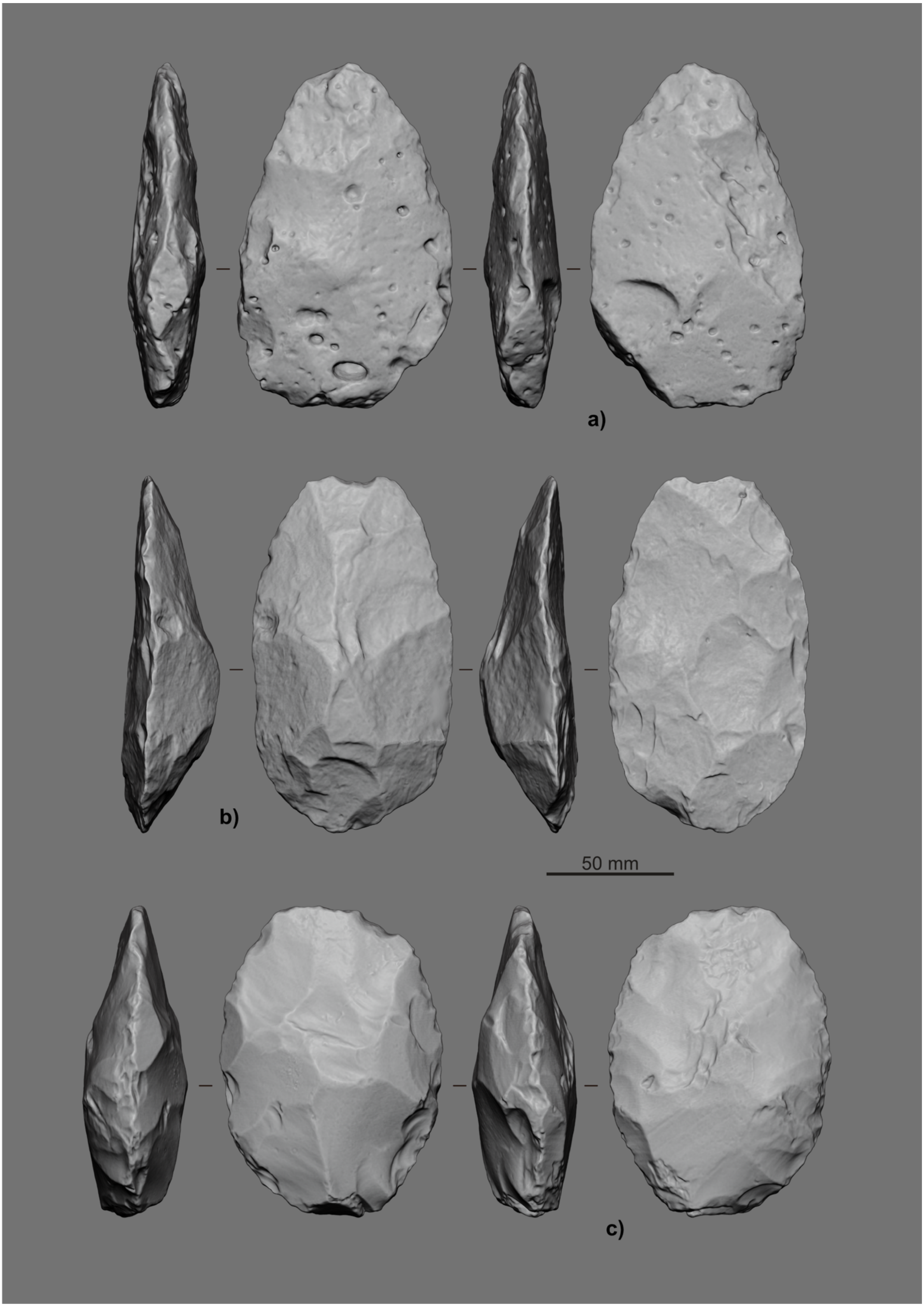

Fig. 3. Handaxes from ATE-II, all of them on CVR (created in Affinity Studio software, using own data).

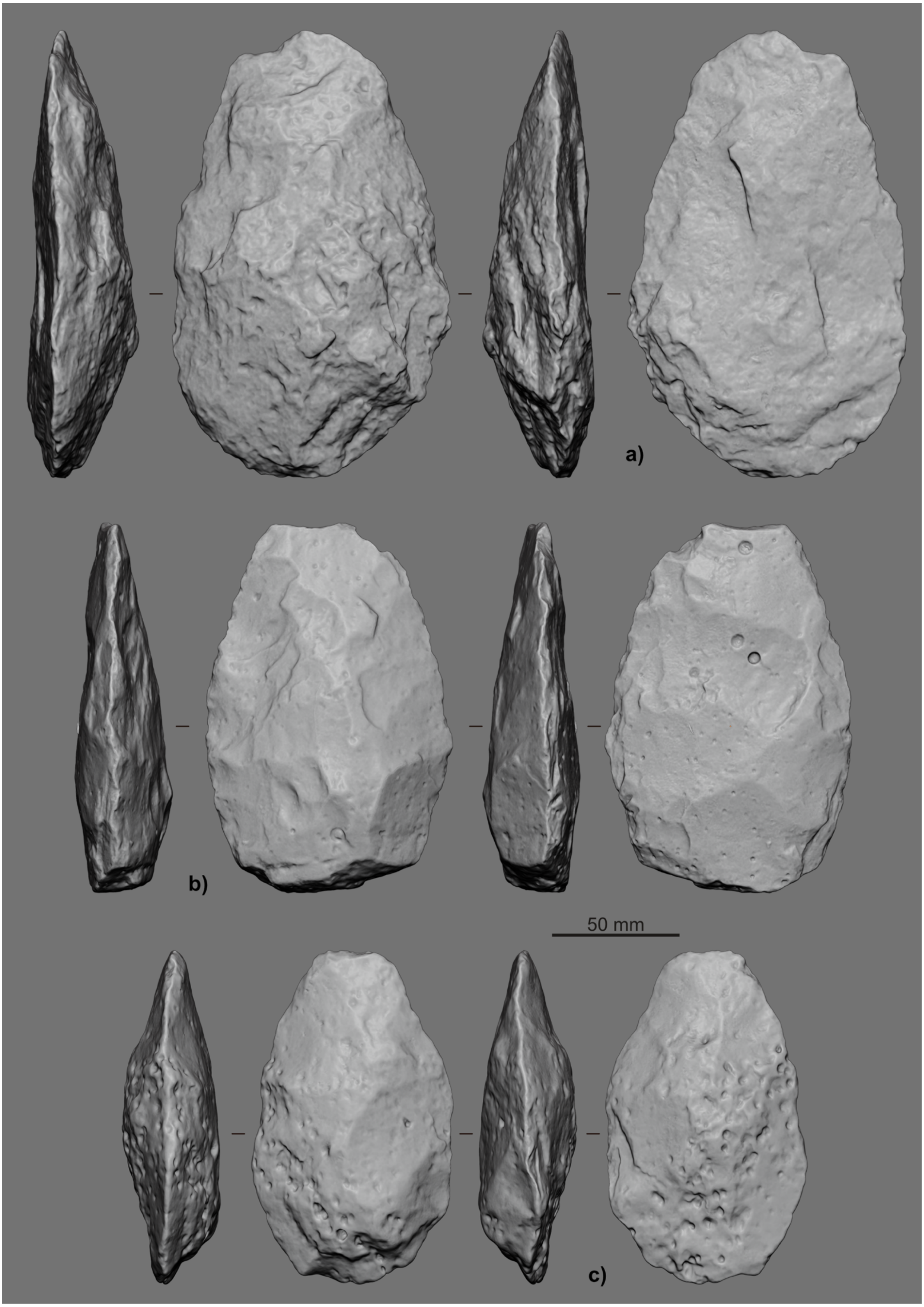

Fig. 4. Handaxes from ATE-II, all of them on CVR (created in Affinity Studio software, using own data).

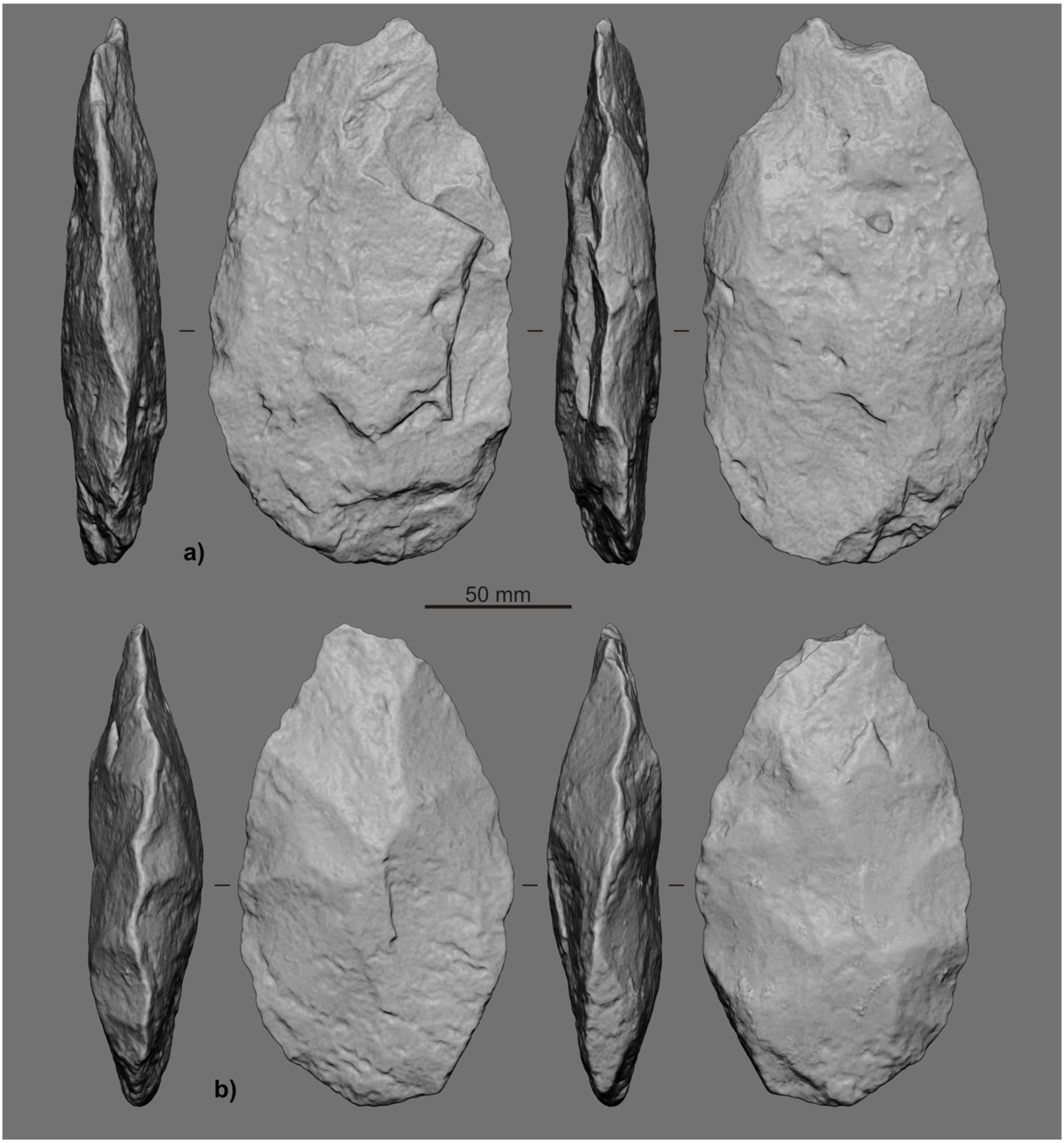

Fig. 5. Handaxes from MS Level B, all of them on CVR (created in Affinity Studio software, using own data).

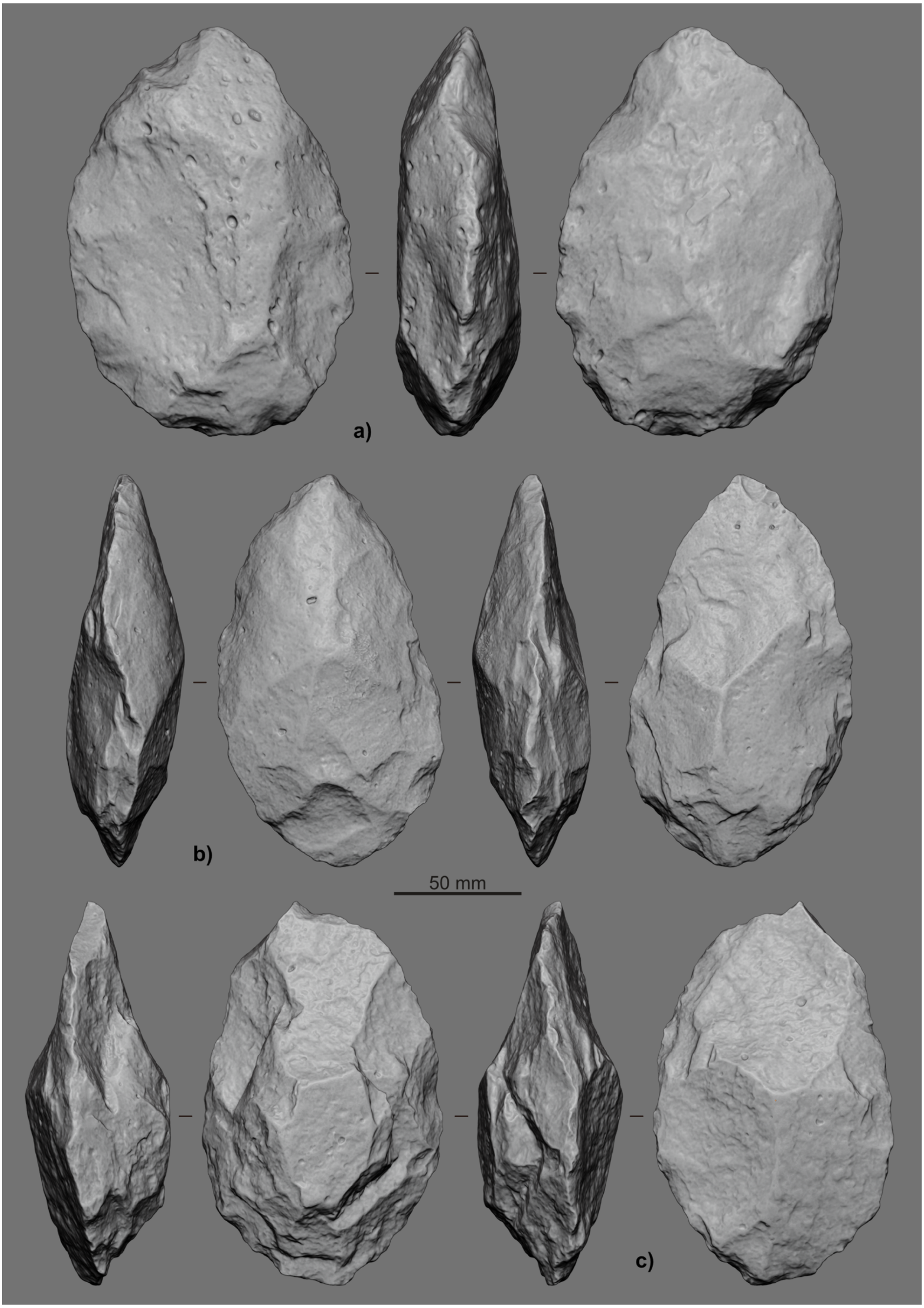

Fig. 6. Handaxes from MS Level B, all of them on CVR (created in Affinity Studio software, using own data).

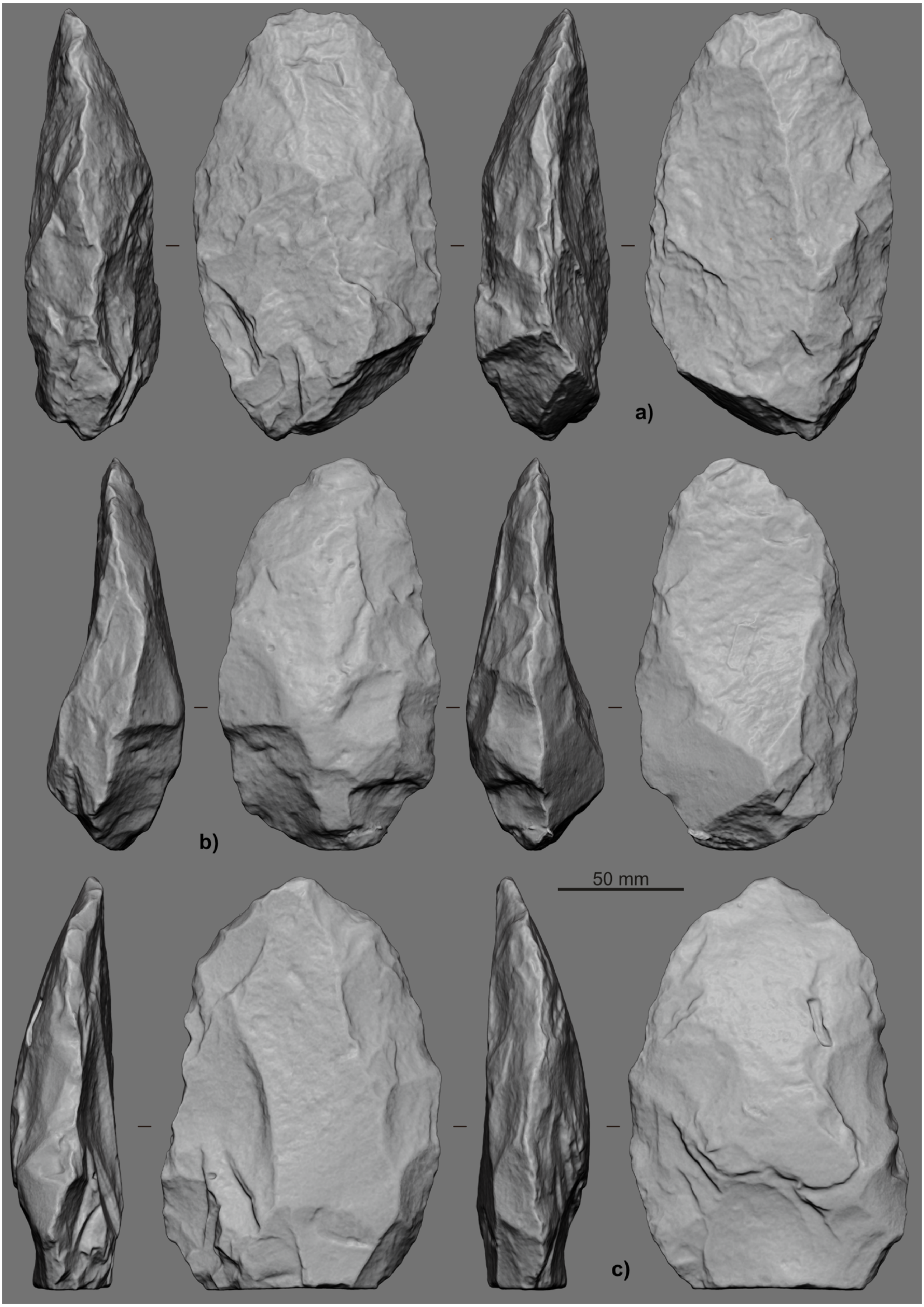

Fig. 7. Diacritical diagrams of handaxes on CVR from ATE-II (a) and MS Level B (b) (created in Affinity Studio software, using own data).

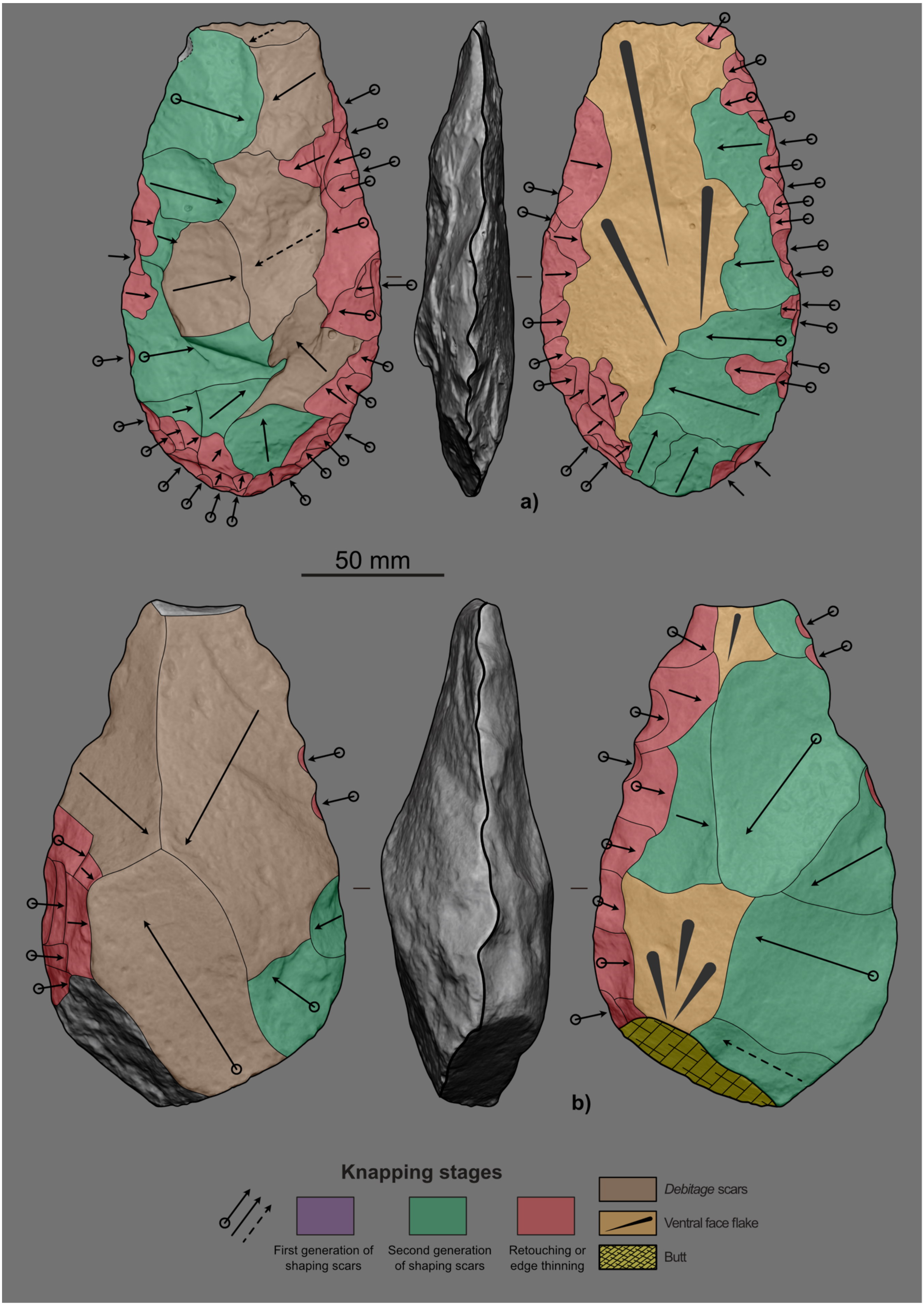

Fig. 8. Drawings and diacritical diagrams of an obsidian handaxe on an indeterminate blank from MS Level C (created in Affinity Studio software, using own data).

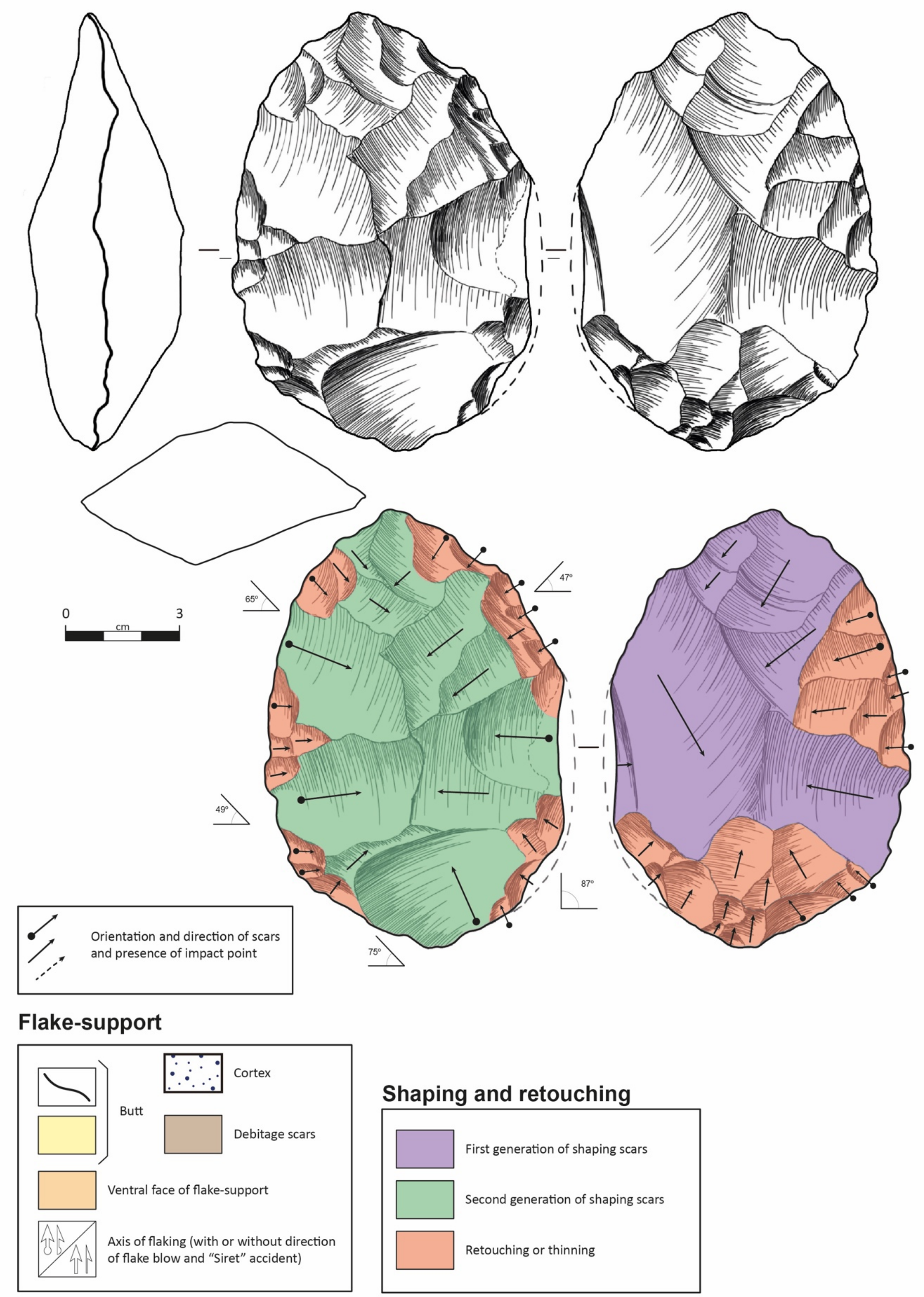

Fig. 9. Pictures, drawings and diacritical diagrams of an obsidian handaxe on an indeterminate blank from MS Level C (created in Affinity Studio software, using own data).

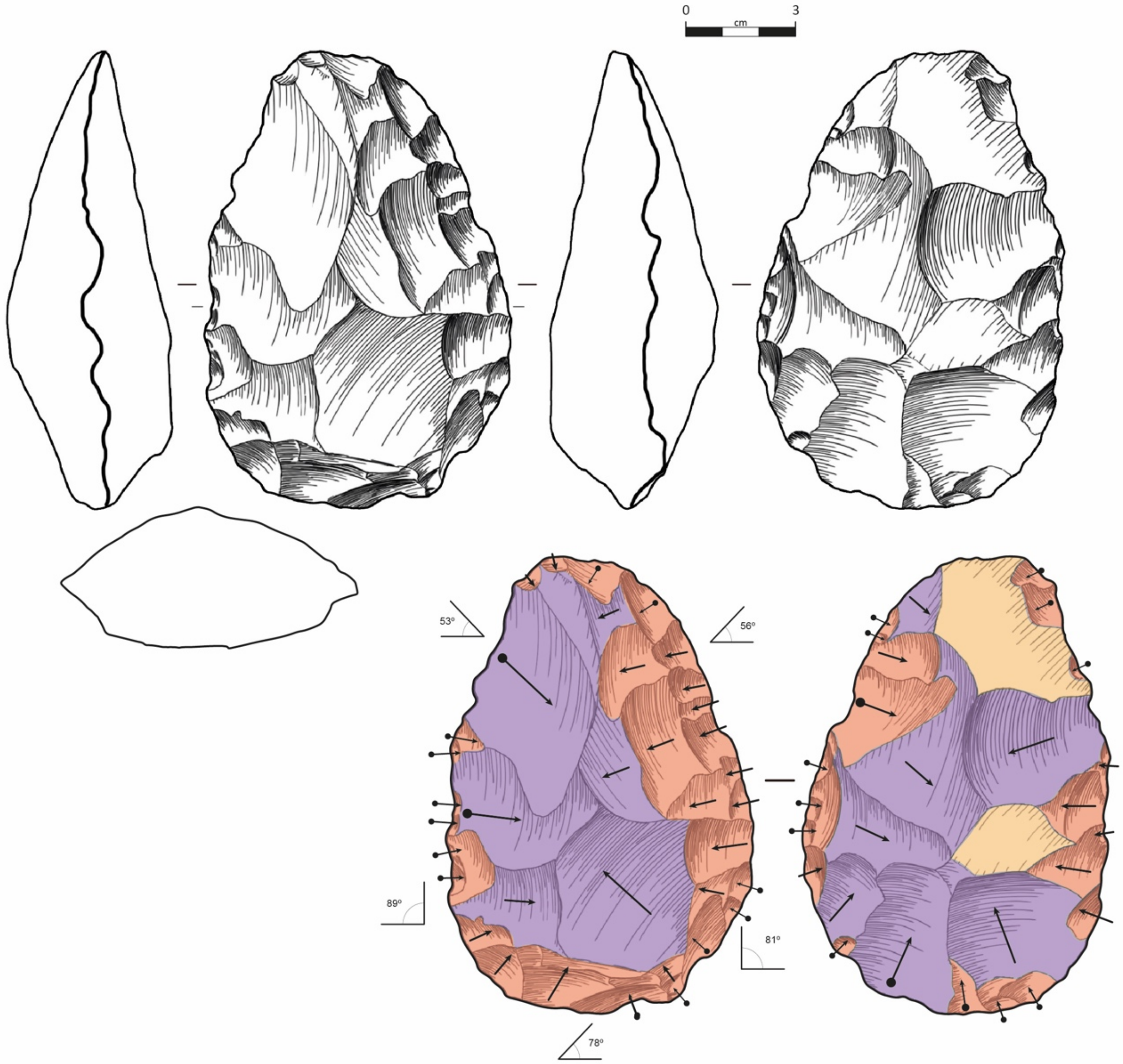

Fig. 10. Pictures, drawings and diacritical diagrams of an obsidian handaxe from MS Level C (created in Affinity Studio software, using own data).

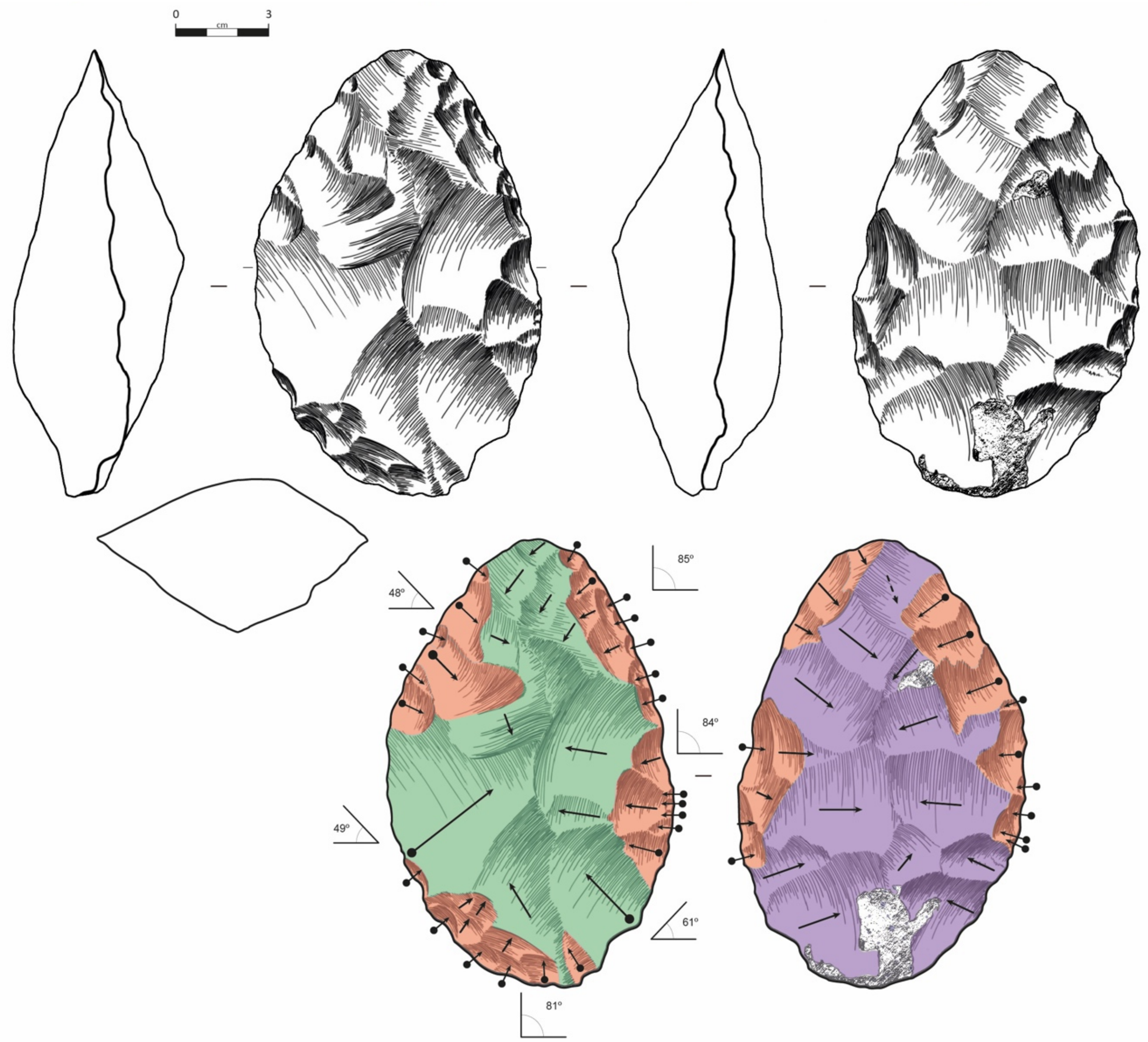

Fig. 11. Pictures, drawings and diacritical diagrams of an obsidian handaxe on flake from MS Level C (created in Affinity Studio software, using own data).

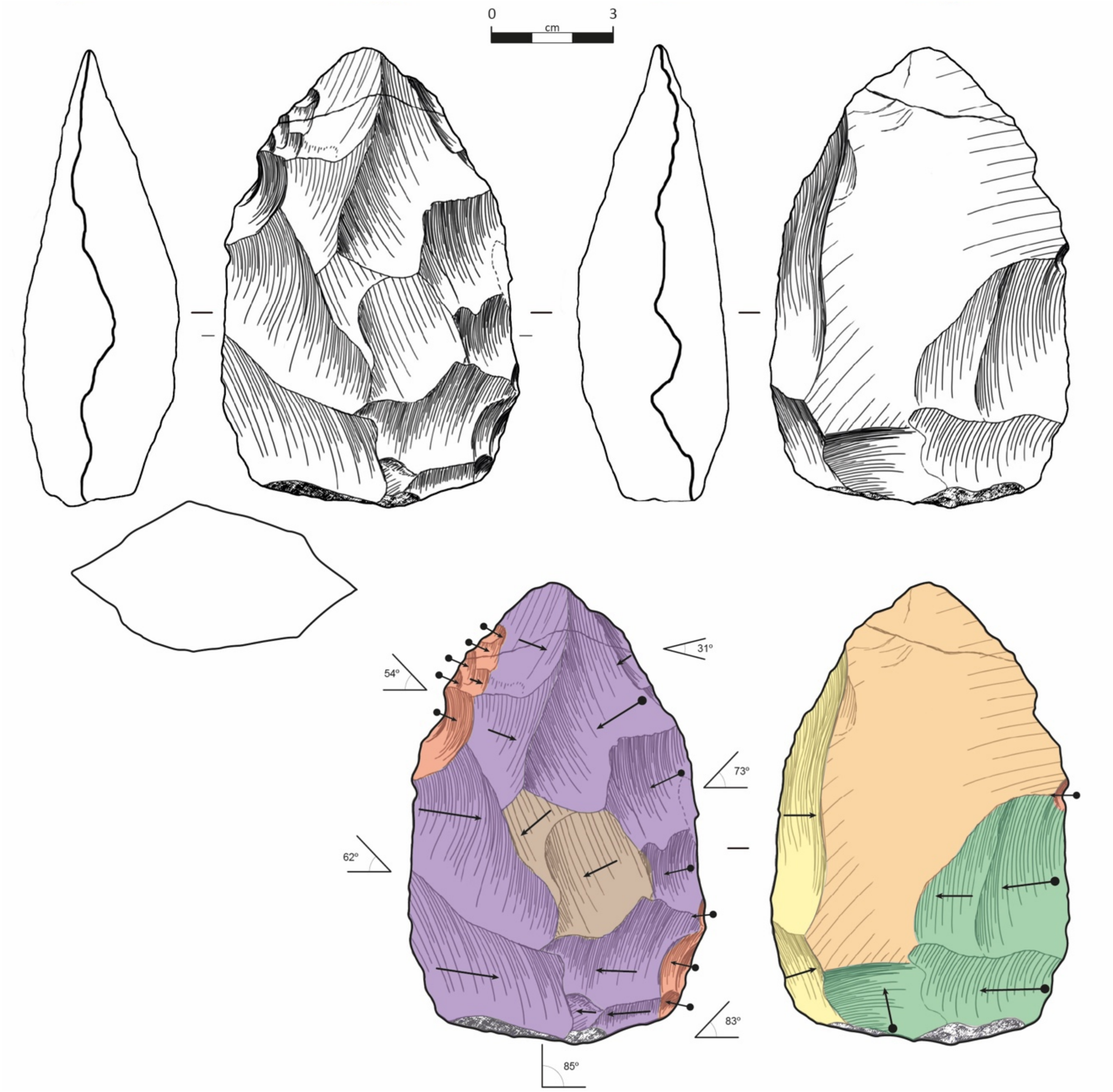

Fig. 12. Pictures, drawings and diacritical diagrams of an obsidian handaxe on flake from MS Level C (created in Affinity Studio software, using own data).

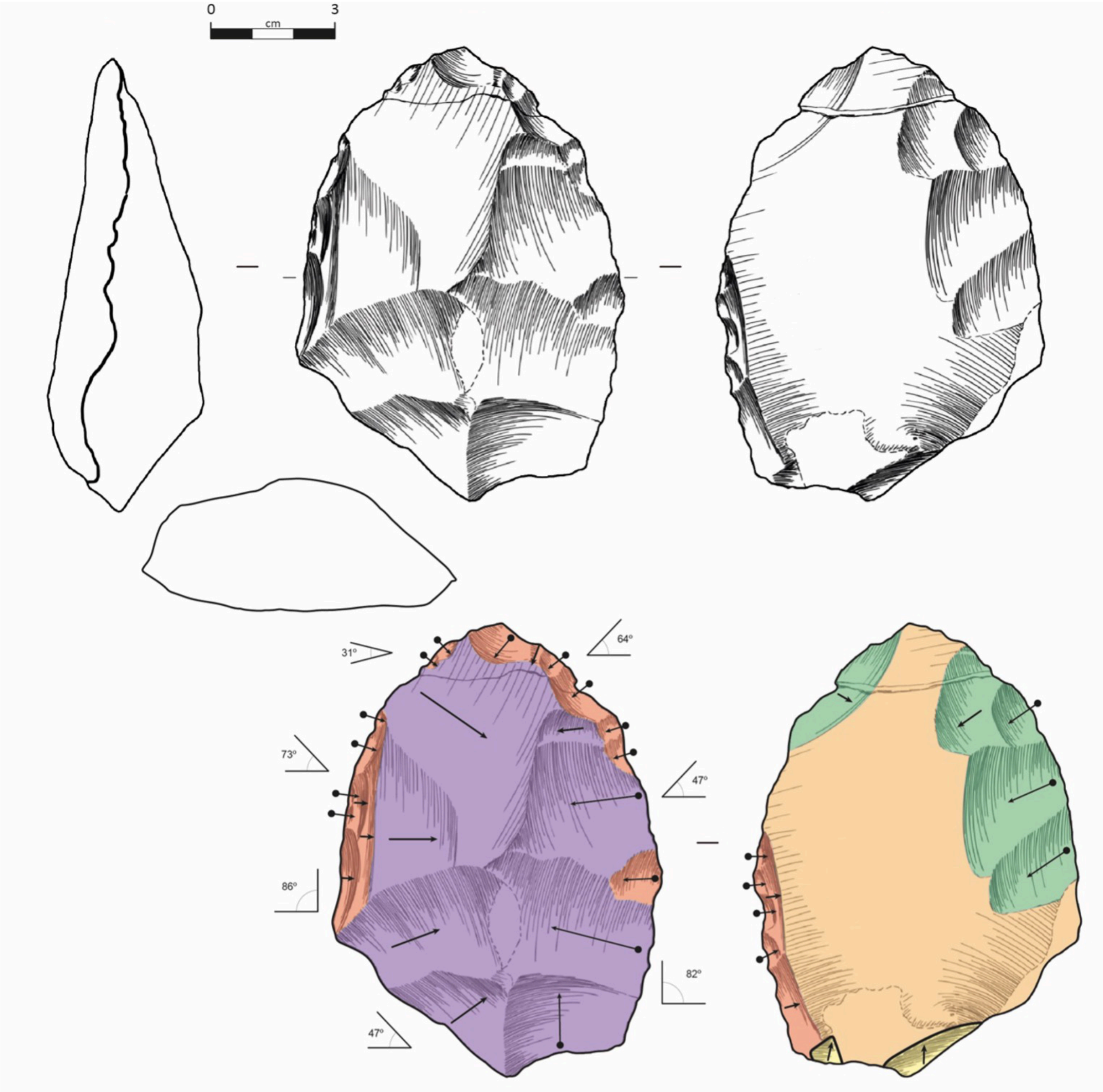

Supplement: Supplementary file 7 [file pone.0357880.s008.pdf]
