# Supplementary material for: Random walks in handaxe knapping before 1.2 million years ago in East Africa at Melka Kunture (Upper Awash, Ethiopia)
Source: PLoS One. 2026 Sep 24;21(9):e0357880. doi: 10.1371/journal.pone.0357880 (PMC13606939; doi:10.1371/journal.pone.0357880)
Supplement: Supplementary file 8 [file pone.0357880.s009.pdf]

Table 1. Raw material percentages in the various assemblages.

Raw material		Assemblage			
		ATE-II	Simbiro IIIB	Simbiro IIIC	Total
CVR	*n*	16	33	0	49
	%	32.7%	67.3%	0.0%	100
Obsidian	*n*	2	0	13	15
	%	13.3%	0.0%	86.7%	100
Total	*n*	18	33	13	64
	%	28.1%	51.6%	20.3%	100

Table 2. Blank types selected for handaxe shaping (whole assemblage).

Blank	*n*	%	Cumulative %
Cobble	5	7.8%	7.8%
Flake	40	62.5%	70.3%
Indeterminate	19	29.7%	100

Table 3. Blank types selected for handaxe shaping.

Blank		Assemblage			
		ATE-II	Simbiro IIIB	Simbiro IIIC	Total
Cobble	*n*	1	3	1	5
	%	5.3%	9.1%	7.7%	7.8%
Flake	*n*	15	15	10	40
	%	84.2%	45.5%	76.9%	62.5%
Indeterminate	*n*	2	15	2	19
	%	10.5%	45.5%	15.4%	29.7%
Total	*n*	18	33	13	64
	%	100	100	100	100

Table 4. Flake blank orientation.

Flaking direction		Assemblage			
		ATE-II	Simbiro IIIB	Simbiro IIIC	Total
Corner struck	*n*	7	5	3	15
	%	46.7%	33.3%	30.0%	37.5%
End struck	*n*	0	2	0	2
	%	0.0%	13.3%	0.0%	5.0%
Indeterminate	*n*	7	7	7	21
	%	46.7%	46.7%	70.0%	52.5%
Side struck	*n*	1	1	0	2
	%	6.7%	6.7%	0.0%	5.0%
Total	*n*	15	15	10	40
	%	100	100	100	100

Table 5. Type of butts on flake supports.

Butt		Assemblage			
		ATE-II	Simbiro IIIB	Simbiro IIIC	Total
Cortical	*n*	1	1	1	3
	%	6.7%	6.7%	10.0%	7.5%
Dihedral	*n*	0	1	0	1
	%	0.0%	6.7%	0.0%	2.5%
Indetermined	*n*	3	3	1	7
	%	20.0%	20.0%	10.0%	17.5%
Plain	*n*	1	1	0	2
	%	6.7%	6.7%	0.0%	5.0%
Parc. remove	*n*	1	0	2	3
	%	6.7%	0.0%	20.0%	7.5%
Whole remove	*n*	9	9	6	24
	%	60.0%	60.0%	60.0%	60.0%
Total	*n*	15	15	10	40
	%	100	100	100	100

Table 6. Presence/absence of thinning in the assemblages.

Thinning		Assemblage			
		ATE-II	Simbiro IIIB	Simbiro IIIC	Total
No	*n*	2	32	2	36
	%	5.6%	88.9%	5.6%	100
Yes	*n*	16	1	11	28
	%	57.1%	3.6%	39.3%	100
Total	*n*	18	33	13	64
	%	28.1%	51.6%	20.3%	100

Table 7. Presence/absence of thinning according to the main raw material.

Raw material		Thinning		
		No	Yes	Total
CVR	*n*	25	24	49
	%	51.0%	49.0%	100.0%
Obsidian	*n*	3	12	15
	%	20.0%	80.0%	100.0%
Total	*n*	28	36	64
	%	43.8%	56.3%	100.0%

Table 8. Presence/absence of soft hammer shaping in the assemblages.

Soft-hammer: shaping		Assemblage			
		ATE-II	Simbiro IIIB	Simbiro IIIC	Total
No	*n*	3	33	13	49
	%	6.1%	67.3%	26.5%	100
Yes	*n*	15	0	0	15
	%	100	0.0%	0.0%	100
Total	*n*	18	33	13	64
	%	28.1%	51.6%	20.3%	100

Table 9. Presence/absence of soft hammer thinning in the assemblages.

Soft-hammer: thinning		Assemblage			
		ATE-II	Simbiro IIIB	Simbiro IIIC	Total
No	*n*	4	33	13	50
	%	8.0%	66.0%	26.0%	100
Yes	*n*	14	0	0	14
	%	100	0.0%	0.0%	100
Total	*n*	18	33	13	64
	%	28.1%	51.6%	20.3%	100

Table 10. Number of scars recorded on the handaxe surface by assemblage: whole scars, shaping phase scars, thinning phase scars.

	Assemblage	*n*	Mean	SD	Minimum	Maximum
Whole scars	ATE-II	18	43.00	22.95	6	84
	Simbiro IIIB	33	16.18	8.50	7	37
	Simbiro IIIC	13	39.38	16.17	16	58
Shaping scars	ATE-II	18	16.77	7.71	6	30
	Simbiro IIIB	33	15.45	7.64	7	29
	Simbiro IIIC	13	18.07	5.82	10	32
Thinning scars	ATE-II	18	26.22	17.87	5	61
	Simbiro IIIB	33	0.72	4.18	6	24
	Simbiro IIIC	13	21.30	15.10	4	42

Table 11. Number of scars recorded on the handaxe surface by main raw material: whole scars, shaping phase scars, thinning phase scars.

	Raw material	*n*	Mean	SD	Minimum	Maximum
Whole scars	CVR	49	24.02	18.42	6	84
	Obsidian	15	42.87	17.65	16	70
Shaping scars	CVR	49	15.67	7.57	6	30
	Obsidian	15	18.60	6.01	10	32
Thinning scars	CVR	49	8.35	15.26	5	61
	Obsidian	15	24.27	16.02	4	45

Table 12. SDI calculated for the analysed handaxes by assemblage.

	Assemblage	*n*	Mean	SD	Minimum	Maximum
SDI	ATE-II	18	0.426	0.2123	0.084	0.751
	Simbiro IIIB	29	0.157	0.0824	0.072	0.383
	Simbiro IIIC	13	0.668	0.3426	0.200	1.387

Table 13. SDI calculated for the analysed handaxes by raw material.

	Raw material	*n*	Mean	SD	Minimum	Maximum
SDI	CVR	42	0.258	0.168	0.072	0.732
	Obsidian	15	0.673	0.318	0.200	1.387

Table 14. Cutting-edge length, SI and RI indices by assemblage.

	Assemblage	*n*	Mean	SD	Minimum	Maximum
Shaping edge	ATE-II	18	314.222	121.964	81	451
	Simbiro IIIB	33	282.455	161.390	198	455
	Simbiro IIIC	13	169.615	132.831	159	367
Thinning edge	ATE-II	18	207.056	130.348	0	387
	Simbiro IIIB	33	39.848	80.031	0	335
	Simbiro IIIC	13	119.923	104.252	46	271
SI	ATE-II	17	0.839	0.210	0.251	1.000
	Simbiro IIIB	26	0.864	0.144	0.509	1.000
	Simbiro IIIC	9	0.790	0.184	0.524	1.000
RI	ATE-II	17	0.549	0.291	0.000	0.960
	Simbiro IIIB	26	0.122	0.214	0.000	0.823
	Simbiro IIIC	9	0.567	0.233	0.131	0.802

Table 15. Cutting-edge length, SI and RI indices by raw material.

	Raw material	*n*	Mean	SD	Minimum	Maximum
Shaping edge	CVR	49	291.061	150.382	81	455
	Obsidian	15	194.667	140.676	159	403
Thinning edge	CVR	49	89.571	121.127	0	387
	Obsidian	15	147.467	122.972	46	387
SI	CVR	41	0.849	0.174	0.251	1.000
	Obsidian	11	0.820	0.179	0.524	1.000
RI	CVR	41	0.262	0.302	0.000	0.904
	Obsidian	11	0.622	0.245	0.131	0.960

Table 16. Main shapes observed within the handaxe assemblage.

Form	*n*	%	Cumulative %
Amygdaloid	37	57.8%	57.8%
Chisel-edge	7	10.9%	68.8%
Diverse	3	4.7%	73.4%
Lanceolate	2	3.1%	76.6%
Naviform	4	6.3%	82.8%
Ovates	11	17.2%	100

Table 17. Main observed shapes observed by handaxe assemblage.

Form		Assemblage			
		ATE-II	Simbiro IIIB	Simbiro IIIC	Total
Amygdaloid	*n*	12	17	8	37
	%	66.7%	51.5%	61.5%	57.8%
Chisel-edge	*n*	3	4	0	7
	%	16.7%	12.1%	0.0%	10.9%
Diverse	*n*	0	3	0	3
	%	0.0%	9.1%	0.0%	4.7%
Lanceolate	*n*	0	2	0	2
	%	0.0%	6.1%	0.0%	3.1%
Naviform	*n*	1	3	0	4
	%	5.6%	9.1%	0.0%	6.3%
Ovates	*n*	2	4	5	11
	%	11.1%	12.1%	38.5%	17.2%
Total	*n*	18	33	13	64
	%	100	100	100	100

Table 18. Handaxe symmetry degree by analysed assemblage.

	Assemblage	*n*	Mean	SD	Minimum	Maximum
Symmetry	ATE-II	18	2.88	0.653	2.01	4.60
	Simbiro IIIB	29	3.96	1.346	2.28	8.04
	Simbiro IIIC	12	3.41	1.337	1.58	6.94

Table 19. Handaxe symmetry degree by raw material.

	Raw material	*n*	Mean	SD	Minimum	Maximum
Symmetry	CVR	45	3.59	1.25	2.01	8.04
	Obsidian	14	3.29	1.27	1.58	6.94

Table 20. First 10 eigenvalues and variance explained by Principal Component Analysis of Procrustes coordinates.

Eigenvalues		% Variance	% Cumulative
1	0.00262562	44.026	44.026
2	0.00097969	16.427	60.453
3	0.00057152	9.583	70.036
4	0.00044290	7.426	77.463
5	0.00033034	5.539	83.002
6	0.00021016	3.524	86.526
7	0.00017029	2.855	89.381
8	0.00016268	2.728	92.109
9	0.00008555	1.434	93.544
10	0.00007329	1.229	94.773

Table 21. Mahalanobis distances among analysed assemblages.

	ATE1	Layer B
Layer B	5.3995	
Layer C	8.1146	6.0418

Table 22. Procrustes distances among analysed assemblages.

	ATE1	Layer B
Layer B	0.0306	
Layer C	0.0554	0.0752

Table 23. Mahalanobis distances among main raw materials.

	CVR
Obsidian	5.8637

Table 24. Procrustes distances among main raw materials.

	CVR
Obsidian	0.0698

Table 25. Discriminant Function Analysis (DFA) results: pairwise comparisons by analysed assemblage.

Comparison	Procrustes distance	Mahalanobis distance	T^2
ATE II-Layer B	0.03061070	21.1794	4981.9770; p = 0.4730
ATE II-Layer C	0.05535517	7.6042	436.4737; p = 0.8243
Layer B-Layer C	0.07518835	7.6711	528.2160; p = 0.9105

Table 26. Discriminant Function Analysis (DFA) results: pairwise comparisons by raw material.

Comparison	Procrustes distance	Mahalanobis distance	T^2
CVR-Obsidian	0.06977066	5.8637	386.8112; p = 0.2269

Table 27. Size (mm) and weight (g) values of all analysed handaxes.

	n	Mean	SD	Minimum	Maximum
Length	64	144.0	25.0	71	199
Width	64	89.3	11.9	47	116
Thickness	64	43.3	11.8	21	78
Weight	64	568.6	270.3	56	1506

Table 28. Size (mm) and weight (g) values by assemblage.

	Assemblage	*n*	Mean	SD	Minimum	Maximum
Length	ATE-II	18	148.1	20.58	117	187
	Simbiro IIIB	33	155.2	17.74	113	199
	Simbiro IIIC	13	110.2	14.97	71	136
Width	ATE-II	18	90.3	9.37	74	107
	Simbiro IIIB	33	93.2	10.24	72	116
	Simbiro IIIC	13	77.8	12.39	47	90
Thickness	ATE-II	18	34.5	4.00	27	43
	Simbiro IIIB	33	51.1	10.90	31	78
	Simbiro IIIC	13	35.7	6.54	21	50
Weight	ATE-II	18	478.8	145.94	286	714
	Simbiro IIIB	33	733.2	247.78	356	1506
	Simbiro IIIC	13	274.9	98.41	56	455

Table 29. Size (mm) and weight (g) values by raw material.

	Raw material	*n*	Mean	SD	Minimum	Maximum
Length	CVR	49	153.5	18.66	113	199
	Obsidian	15	113.2	16.76	71	146
Width	CVR	49	92.2	10.09	72	116
	Obsidian	15	79.6	12.51	47	96
Thickness	CVR	49	45.6	12.18	27	78
	Obsidian	15	35.8	6.13	21	50
Weight	CVR	49	652.3	249.17	286	1506
	Obsidian	15	295.0	106.02	56	456

Table 30. Size (mm) and weight (g) values by type of blank.

	Blank	*n*	Mean	SD	Minimum	Maximum
Length	Cobble	5	149.6	26.88	106	173
	Flake	40	141.4	27.61	71	199
	Indeterminate	19	148.0	18.16	108	180
Width	Cobble	5	86.6	12.32	68	98
	Flake	40	89.2	12.64	47	112
	Indeterminate	19	90.2	10.61	74	116
Thickness	Cobble	5	54.0	19.17	34	78
	Flake	40	39.6	9.95	21	65
	Indeterminate	19	48.3	10.06	33	69
Weight	Cobble	5	662.4	315.20	227	1012
	Flake	40	524.6	276.93	56	1506
	Indeterminate	19	636.4	236.47	233	1029
