# Supplementary material for: Random walks in handaxe knapping before 1.2 million years ago in East Africa at Melka Kunture (Upper Awash, Ethiopia)
Source: PLoS One. 2026 Sep 24;21(9):e0357880. doi: 10.1371/journal.pone.0357880 (PMC13606939; doi:10.1371/journal.pone.0357880)

Fig. 1. Extent of the shaped area by main raw material in the analysed assemblages.

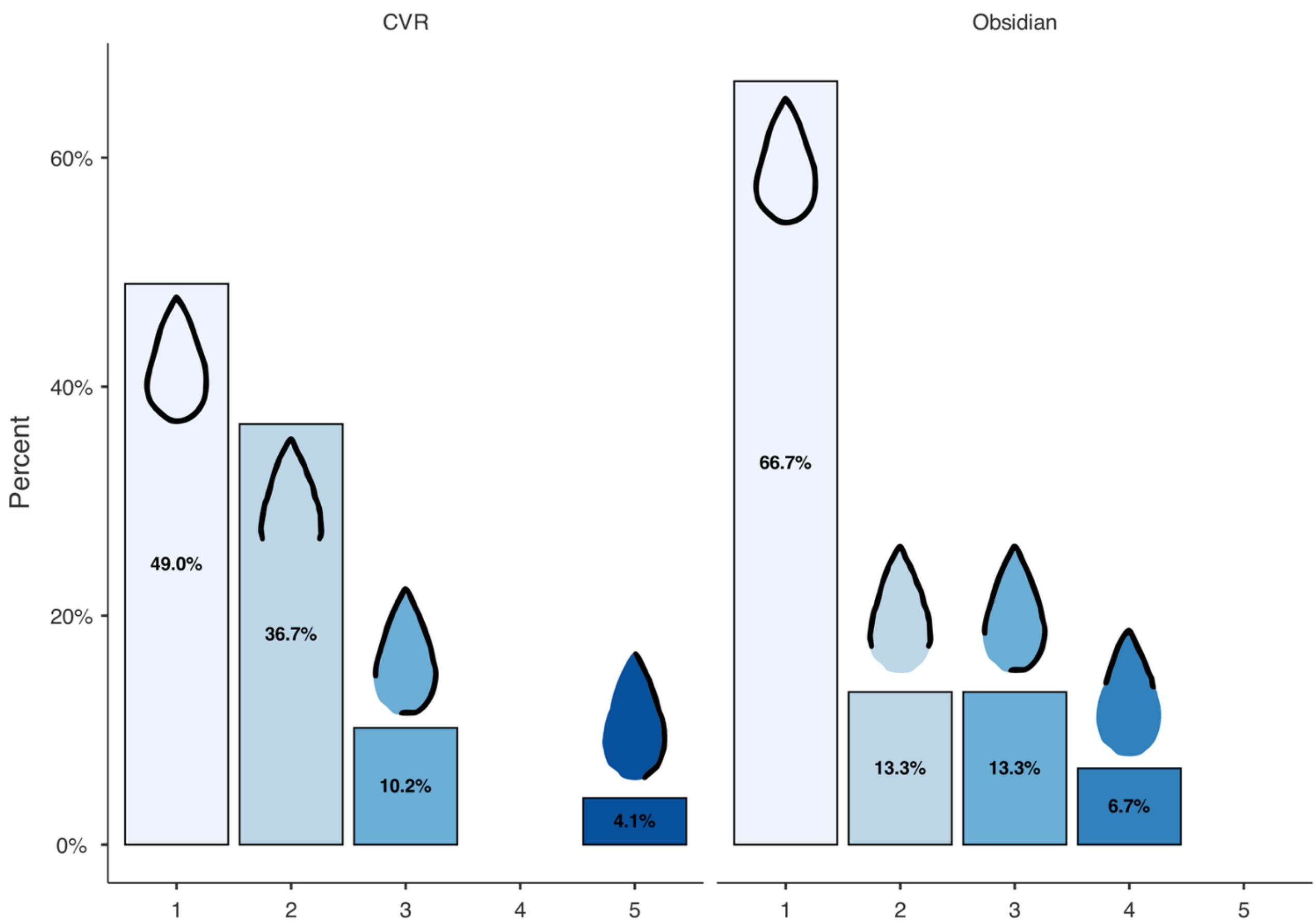

Fig. 2. Box plot showing the distribution of number of scars by raw material in the analysed assemblages.

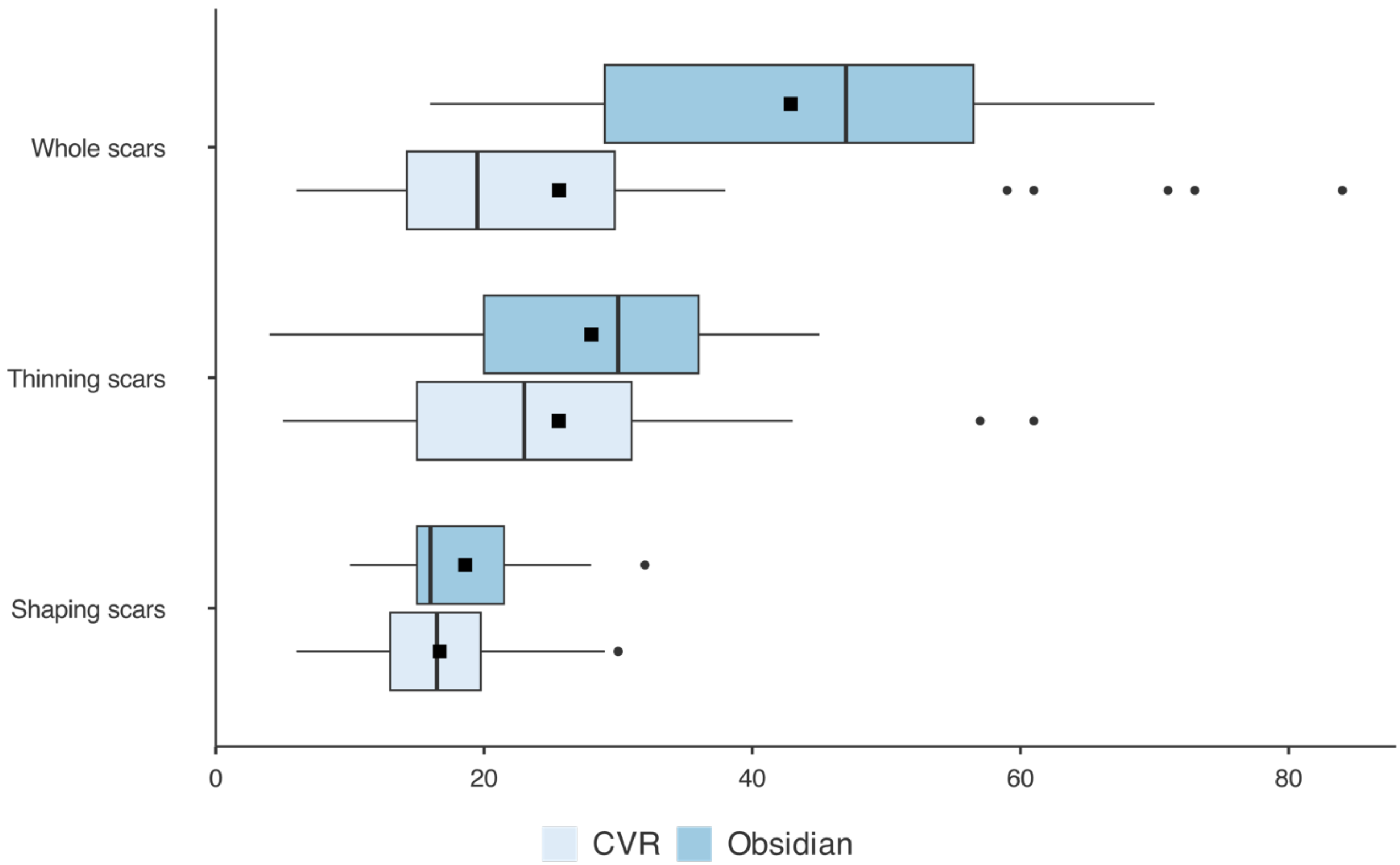

Fig. 3. Box plot showing the SDI values of the analysed handaxes by raw material in the analysed assemblages.

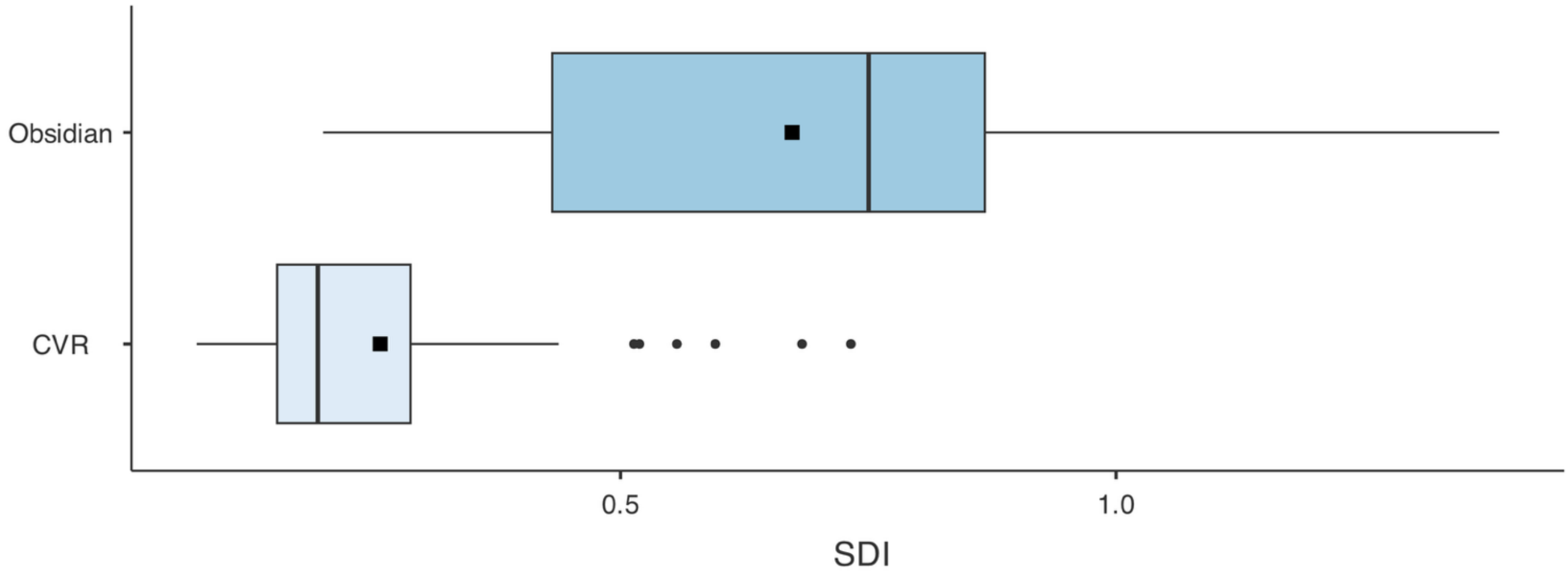

Fig. 4. Box plot showing the RI and SI indices by raw material in the analysed assemblages.

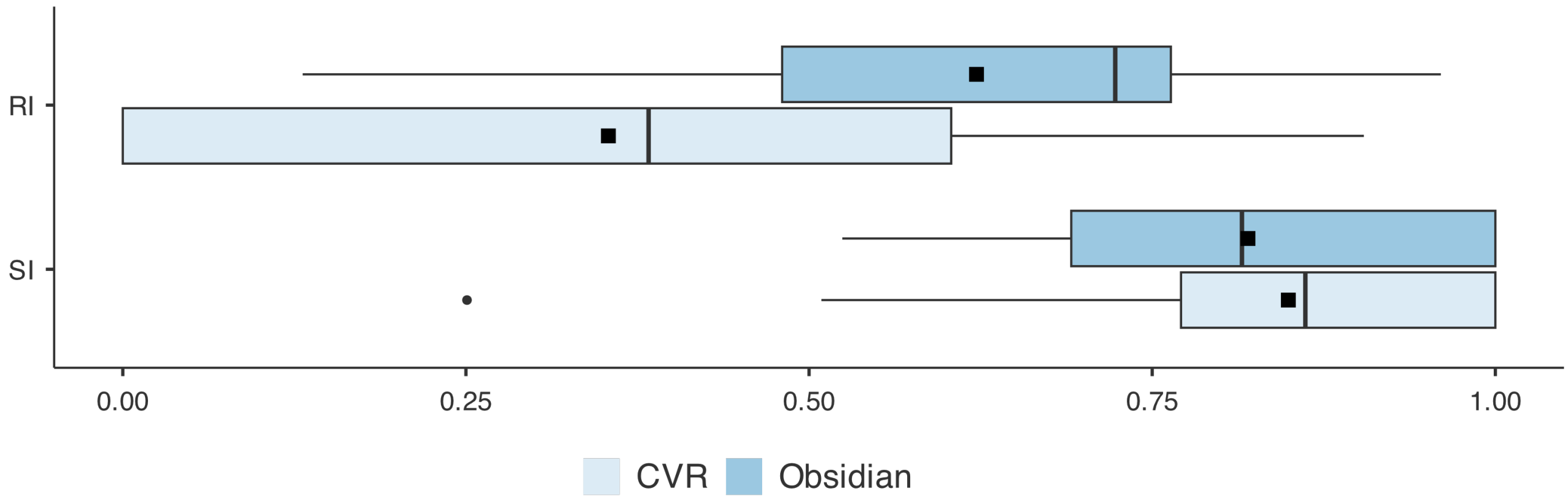

Fig. 5. Box plot with the degree of symmetry by raw material in the analysed assemblages. Values between 2-3 indicate a high degree of symmetry, whereas values >4 denote moderate to low symmetry [13].

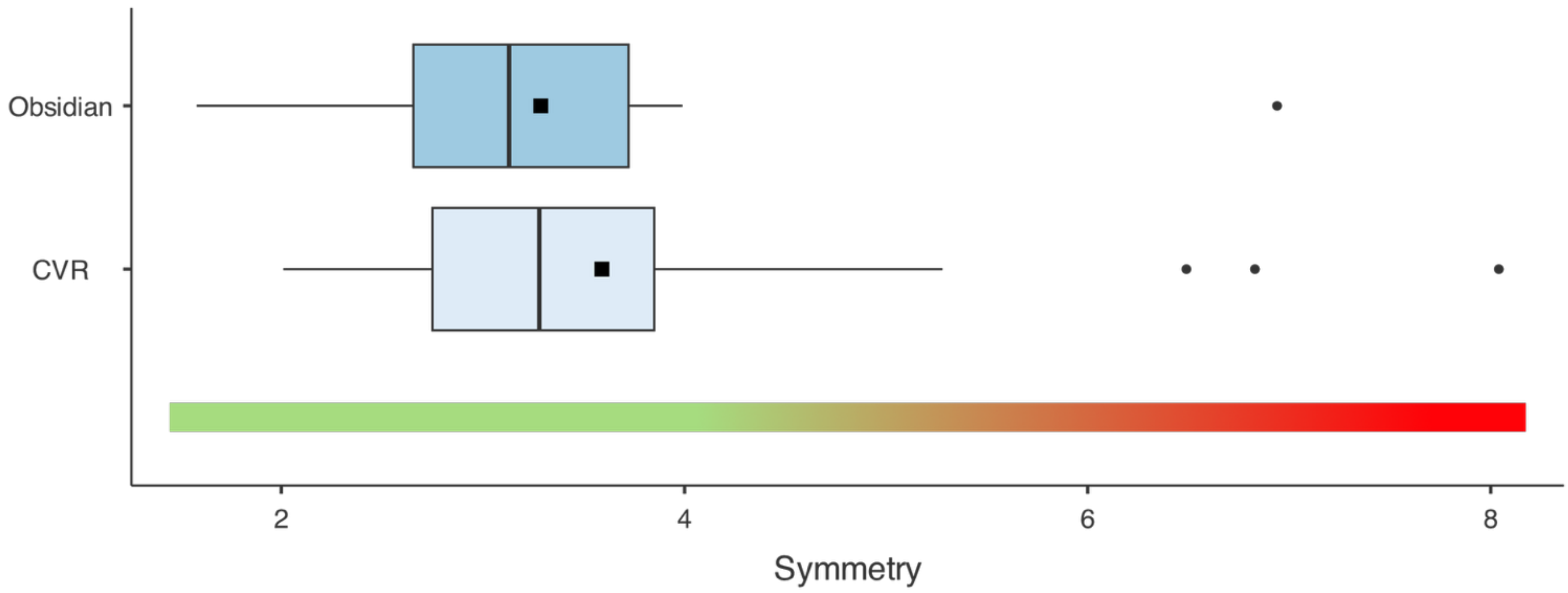

Fig. 6. Box plots showing the distribution of size (mm) by main raw material in the analysed assemblages.

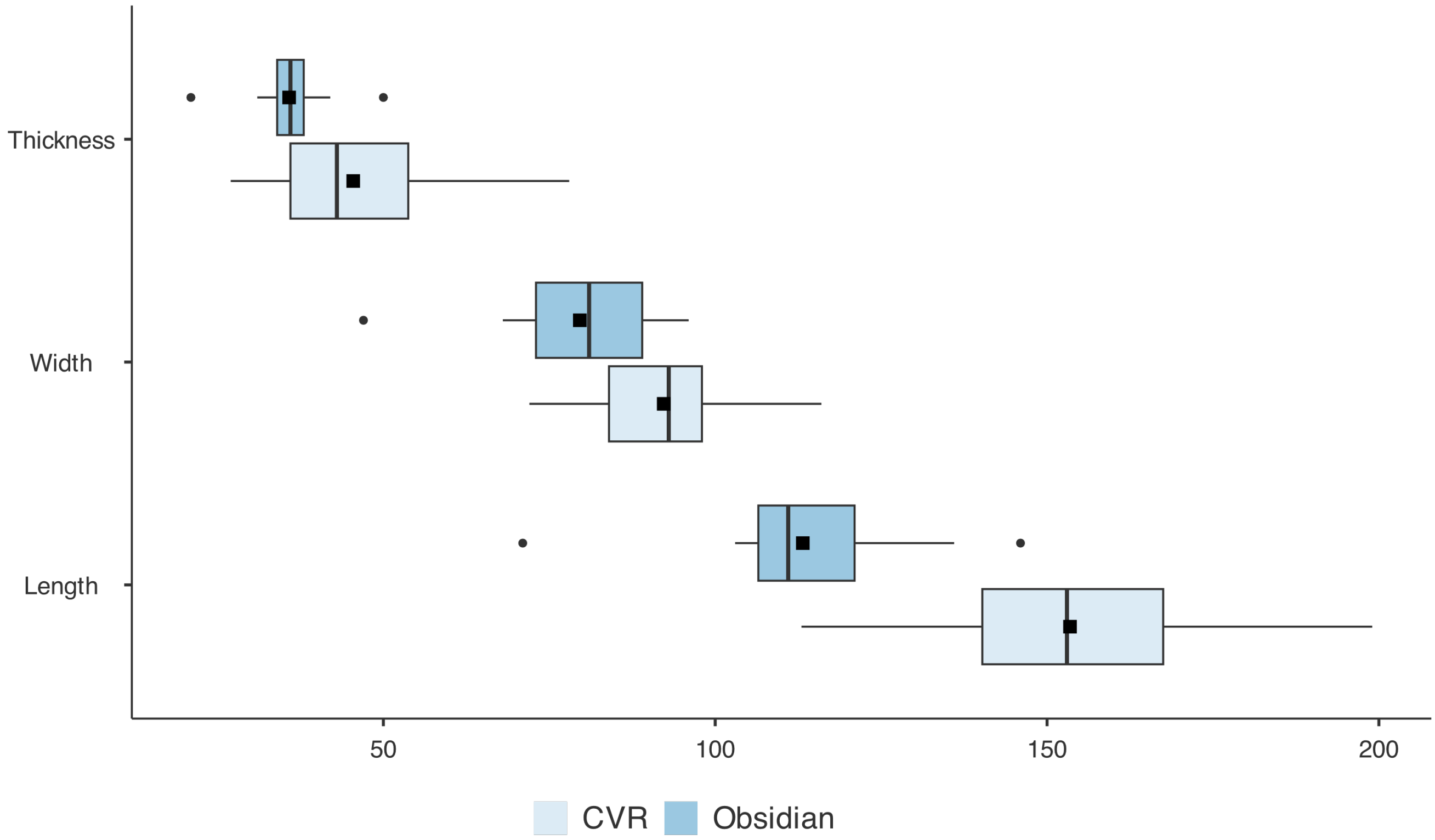

Fig. 7. Box plots showing the distribution of size (mm) by main type of blank in the analysed assemblages.

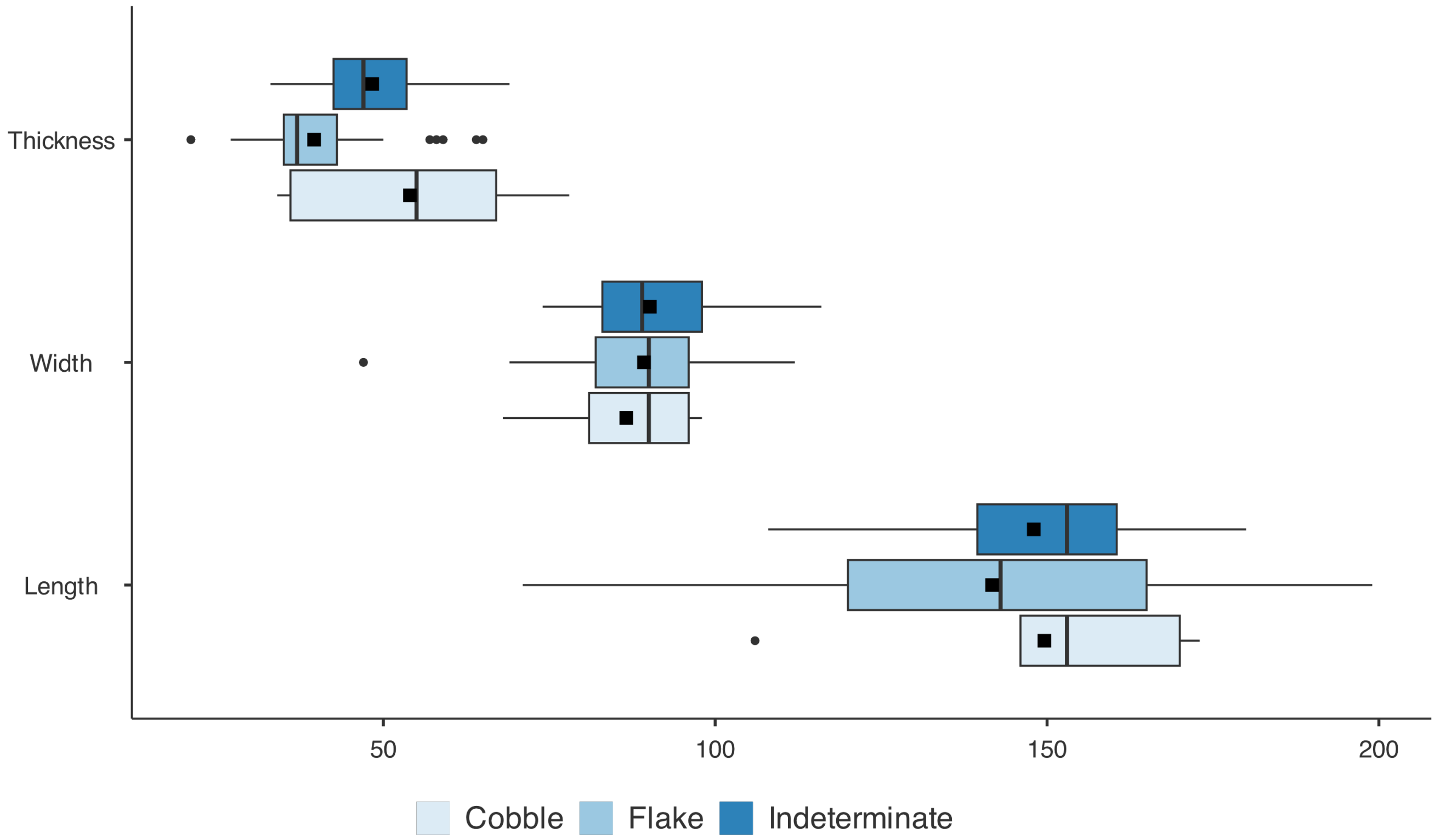

Fig. 8. Box plots showing the distribution of weight (g) by main raw material in the analysed assemblages.

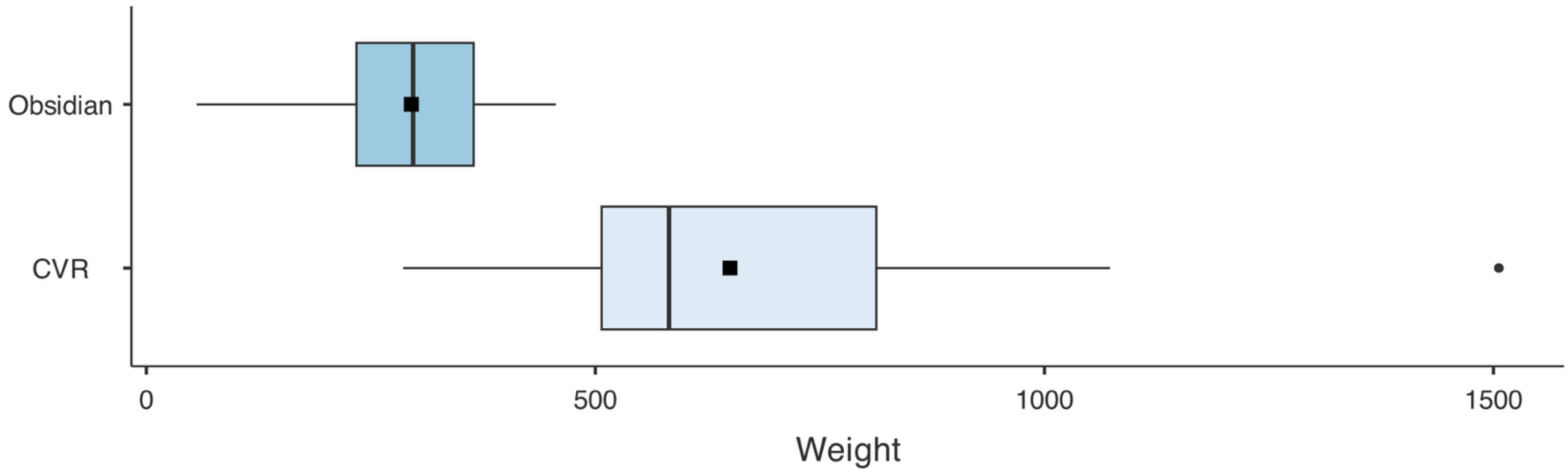

Fig. 9. Box plots showing the distribution of weight (g) by type of blank in the analysed assemblages.

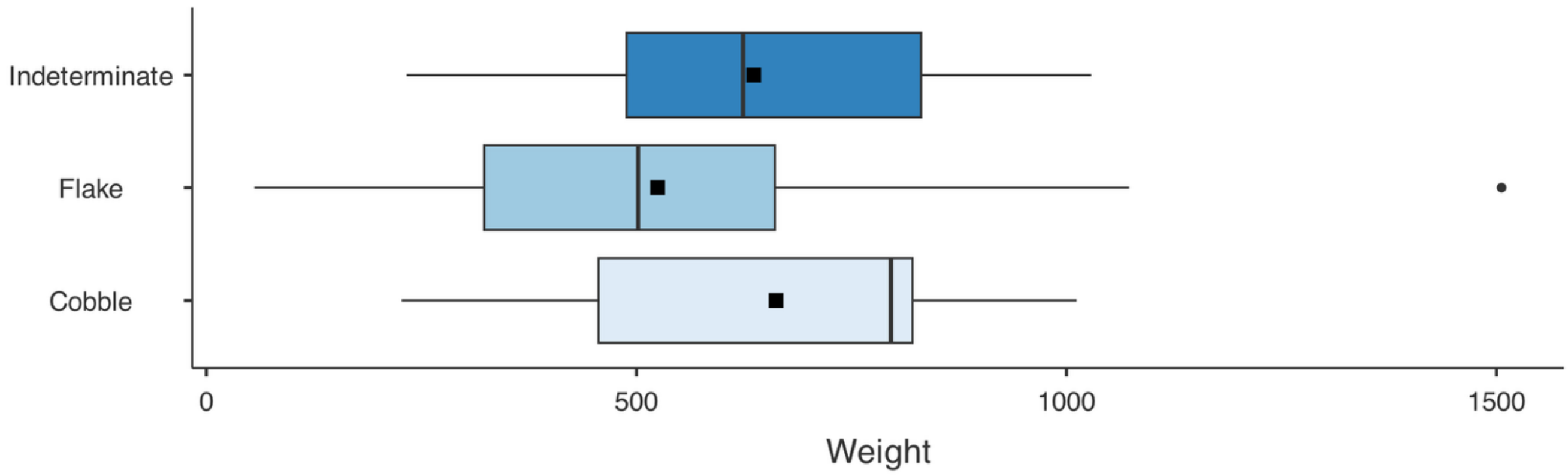

Fig. 10. Scatter plot of Principal Component Analysis (PCA) scores by assemblage.

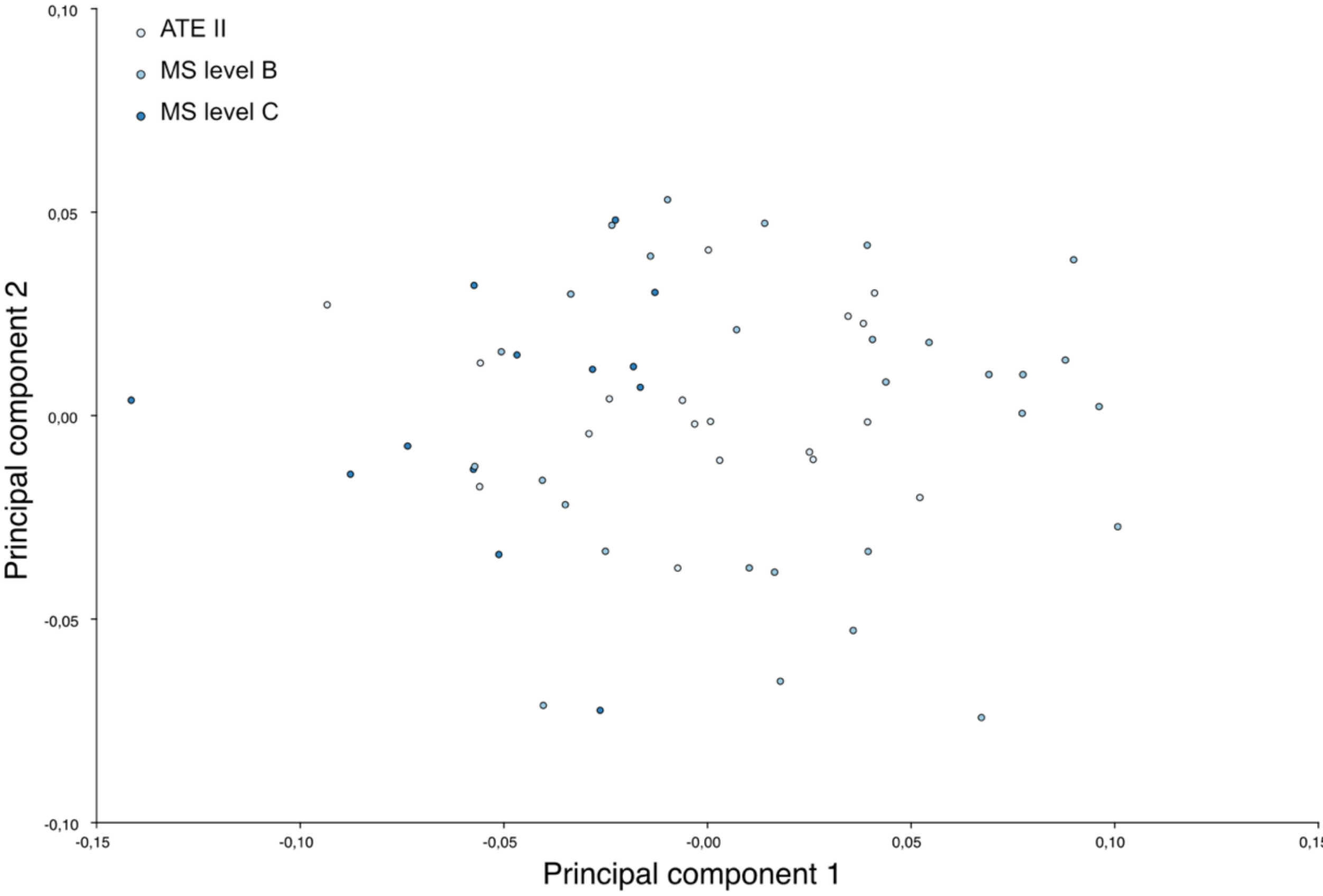

Fig. 11. Scatter plot of Principal Component Analysis (PCA) scores by raw material.

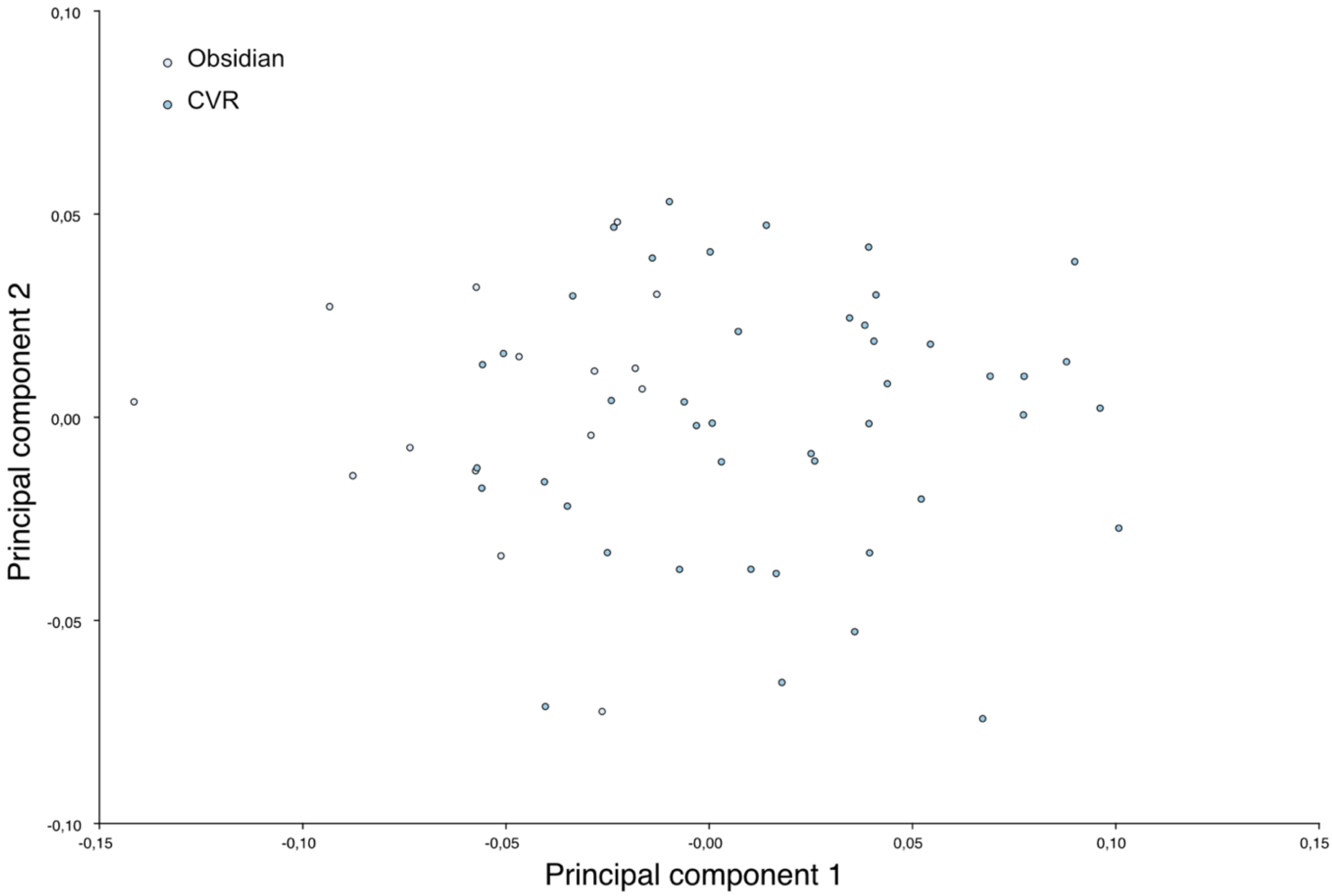

Fig. 12. Scatter plots of the CVA results of the handaxe in the analysed assemblages by raw material (95% confidence ellipses are plotted).

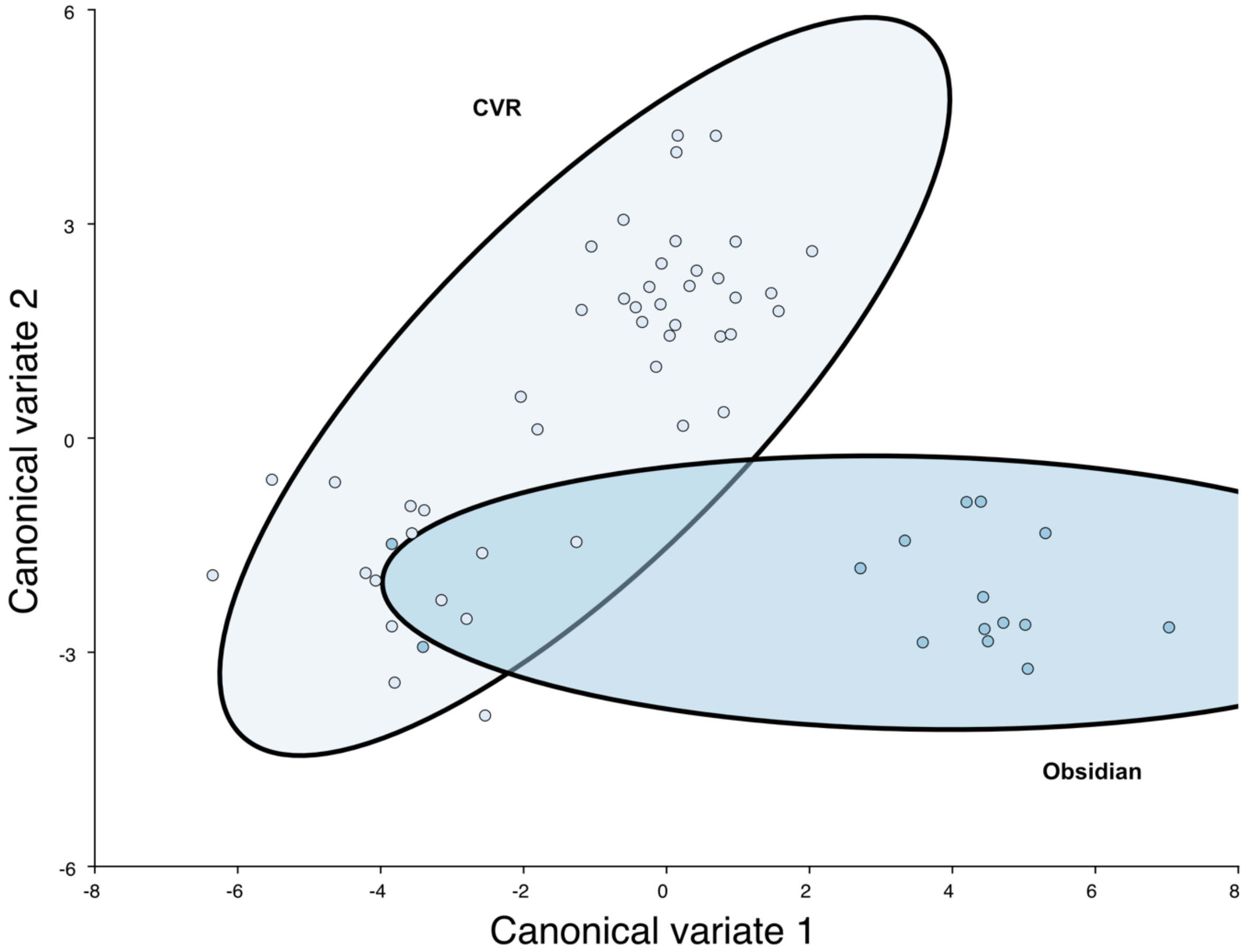

Supplement: Supplementary file 9 [file pone.0357880.s010.pdf]
