# Supplementary material for: Random walks in handaxe knapping before 1.2 million years ago in East Africa at Melka Kunture (Upper Awash, Ethiopia)
Source: PLoS One. 2026 Sep 24;21(9):e0357880. doi: 10.1371/journal.pone.0357880 (PMC13606939; doi:10.1371/journal.pone.0357880)

Fig. 1. Spearman's rank correlation matrix for size, symmetry and refinement variables of all analysed handaxes. Significance levels: * $p < 0.05$, ** $p < 0.01$, *** $p < 0.001$.

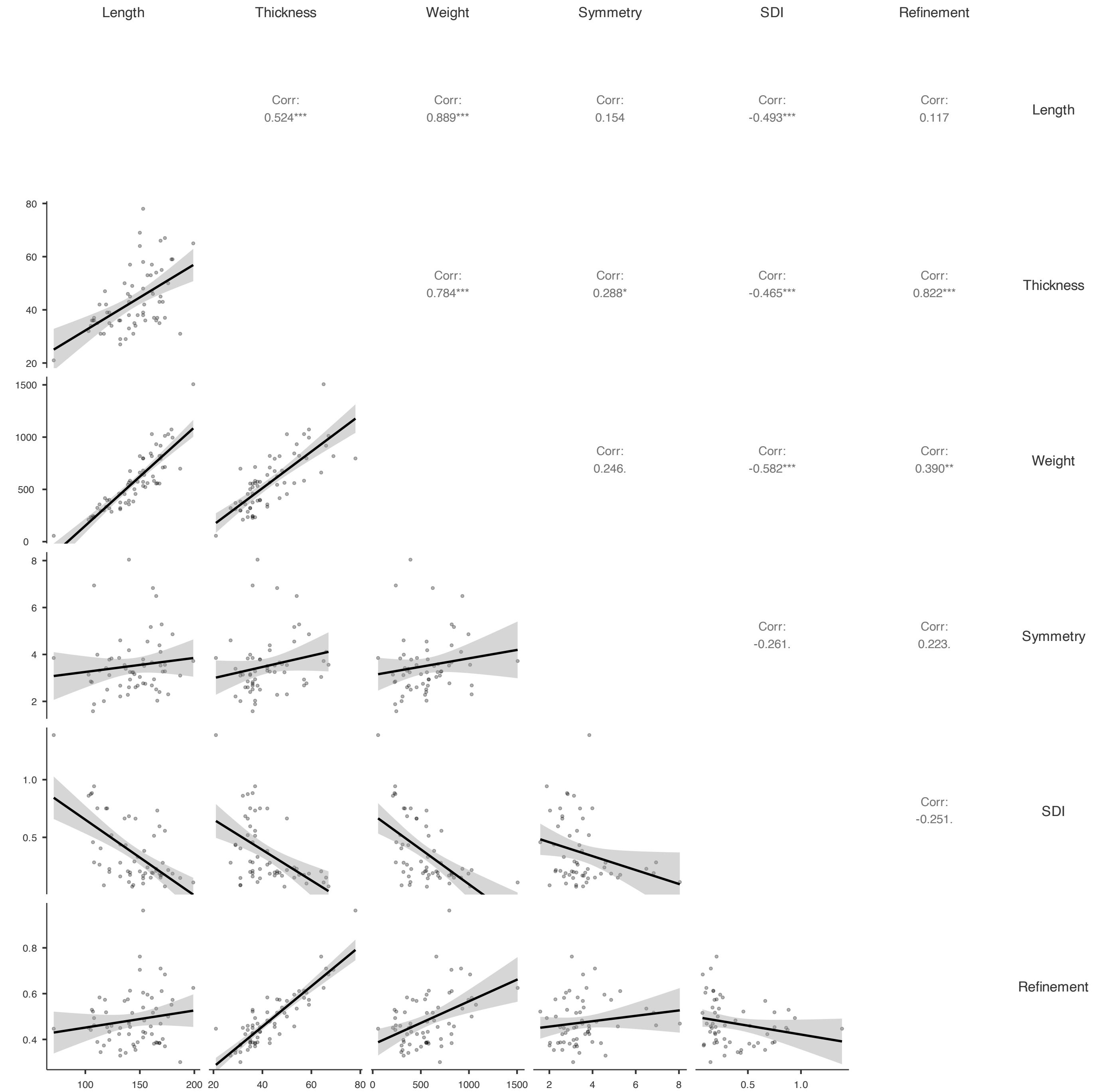

Fig. 2. Spearman's rank correlation matrix for size, symmetry and refinement variables of the ATE-II assemblage. Significance levels: * $p < 0.05$, ** $p < 0.01$, *** $p < 0.001$.

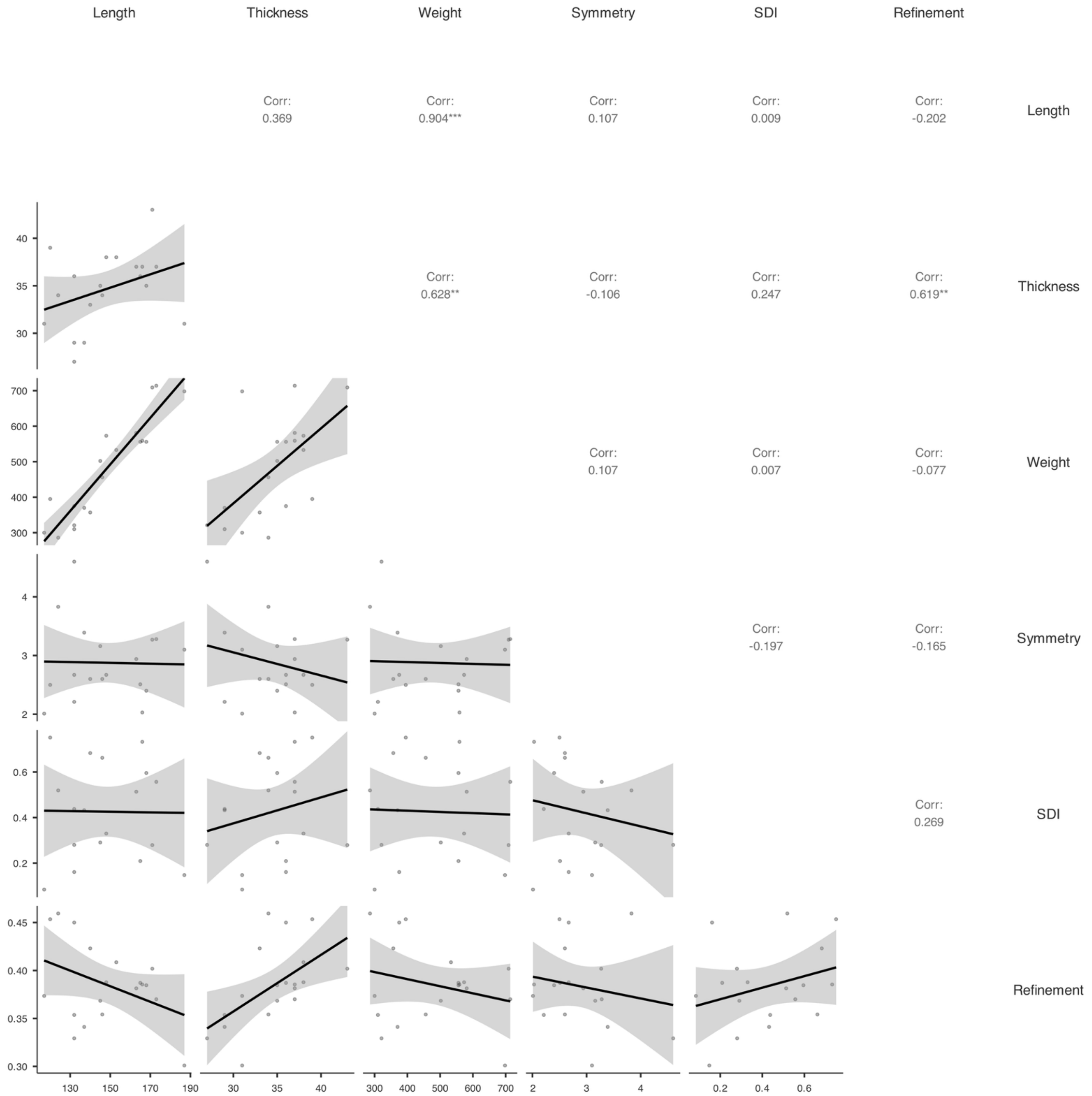

Fig. 3. Spearman's rank correlation matrix for size, symmetry and refinement variables of MS Level B assemblage. Significance levels: * $p < 0.05$, ** $p < 0.01$, *** $p < 0.001$.

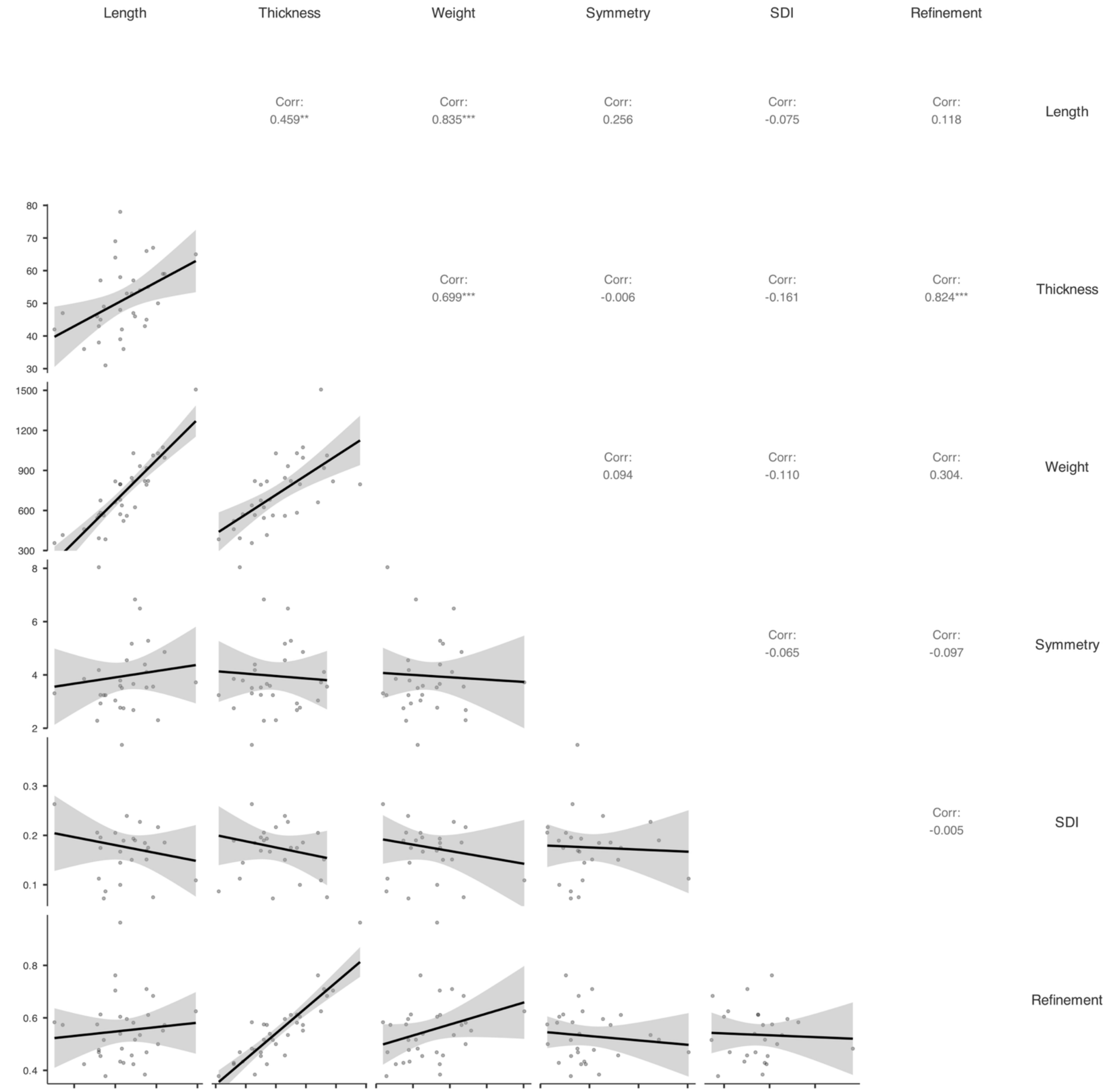

Fig. 4. Spearman's rank correlation matrix for size, symmetry and refinement variables of MS Level C assemblage. Significance levels: * p < 0.05, ** p < 0.01, *** p < 0.001.

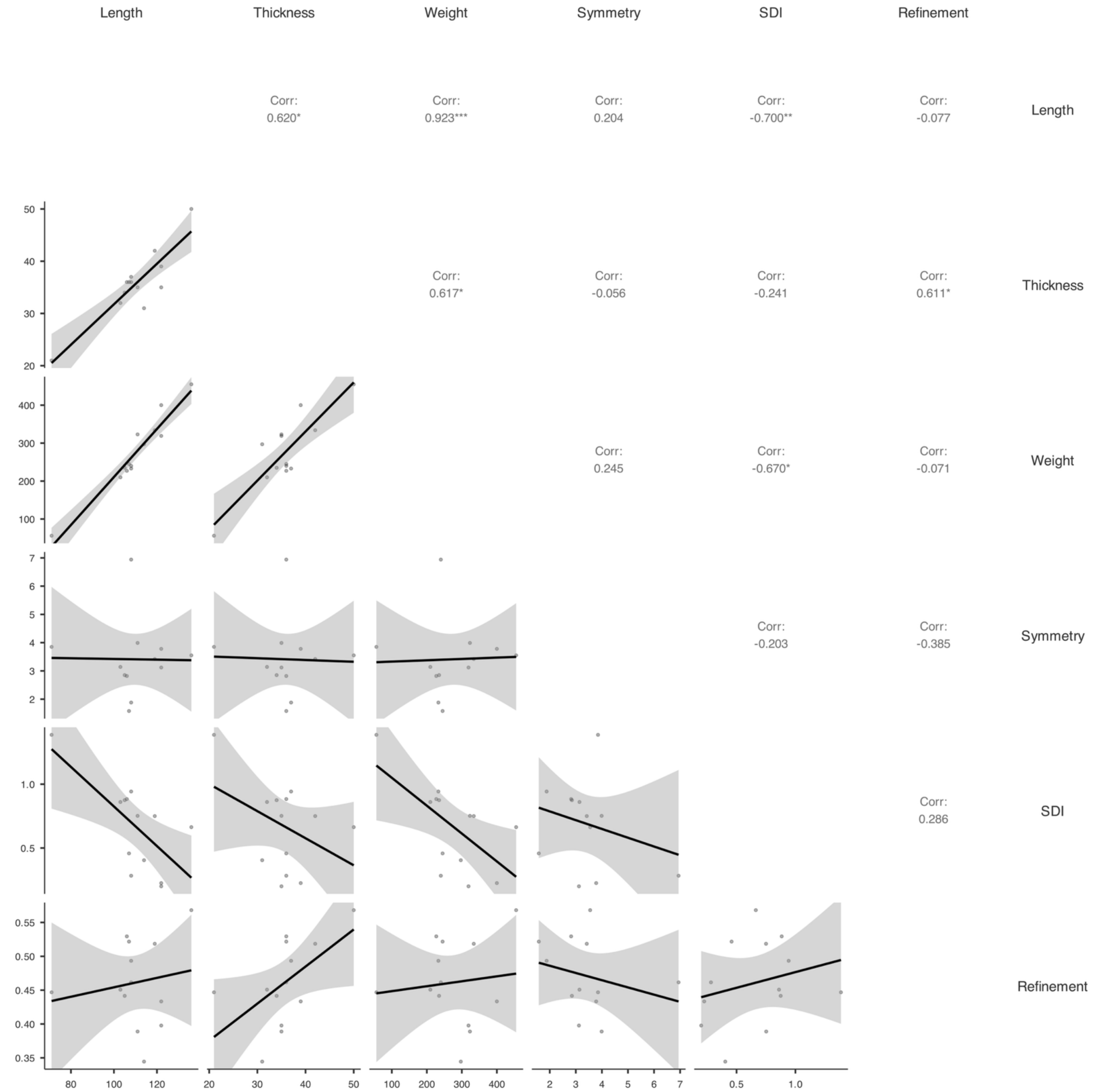

Fig. 5. Spearman's rank correlation matrix for size, symmetry and refinement variables of CVR handaxes. Significance levels: * p < 0.05, ** p < 0.01, *** p < 0.001.

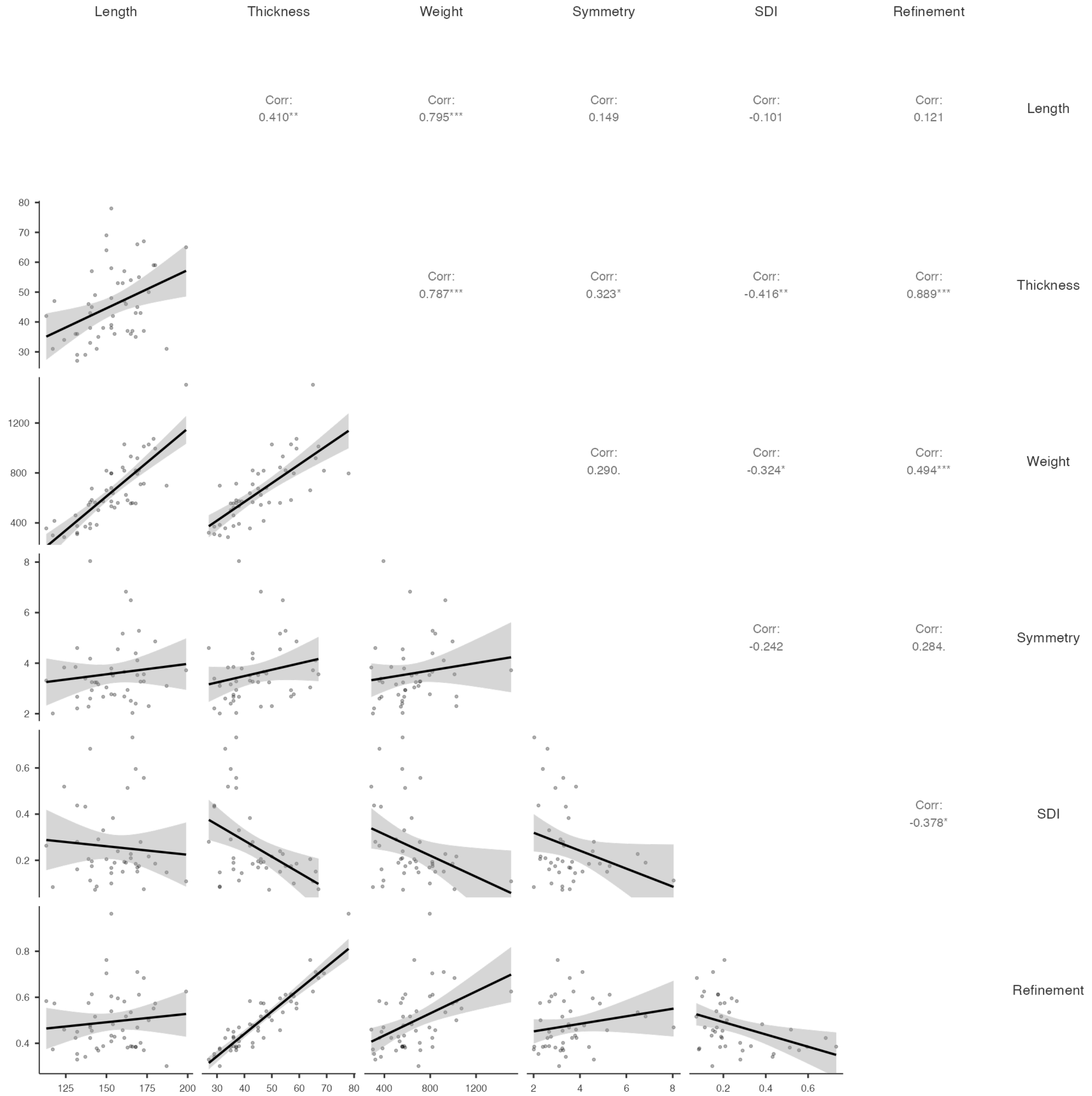

Fig. 6. Spearman's rank correlation matrix for size, symmetry and refinement variables of obsidian handaxes. Significance levels: * $p < 0.05$, ** $p < 0.01$, *** $p < 0.001$.

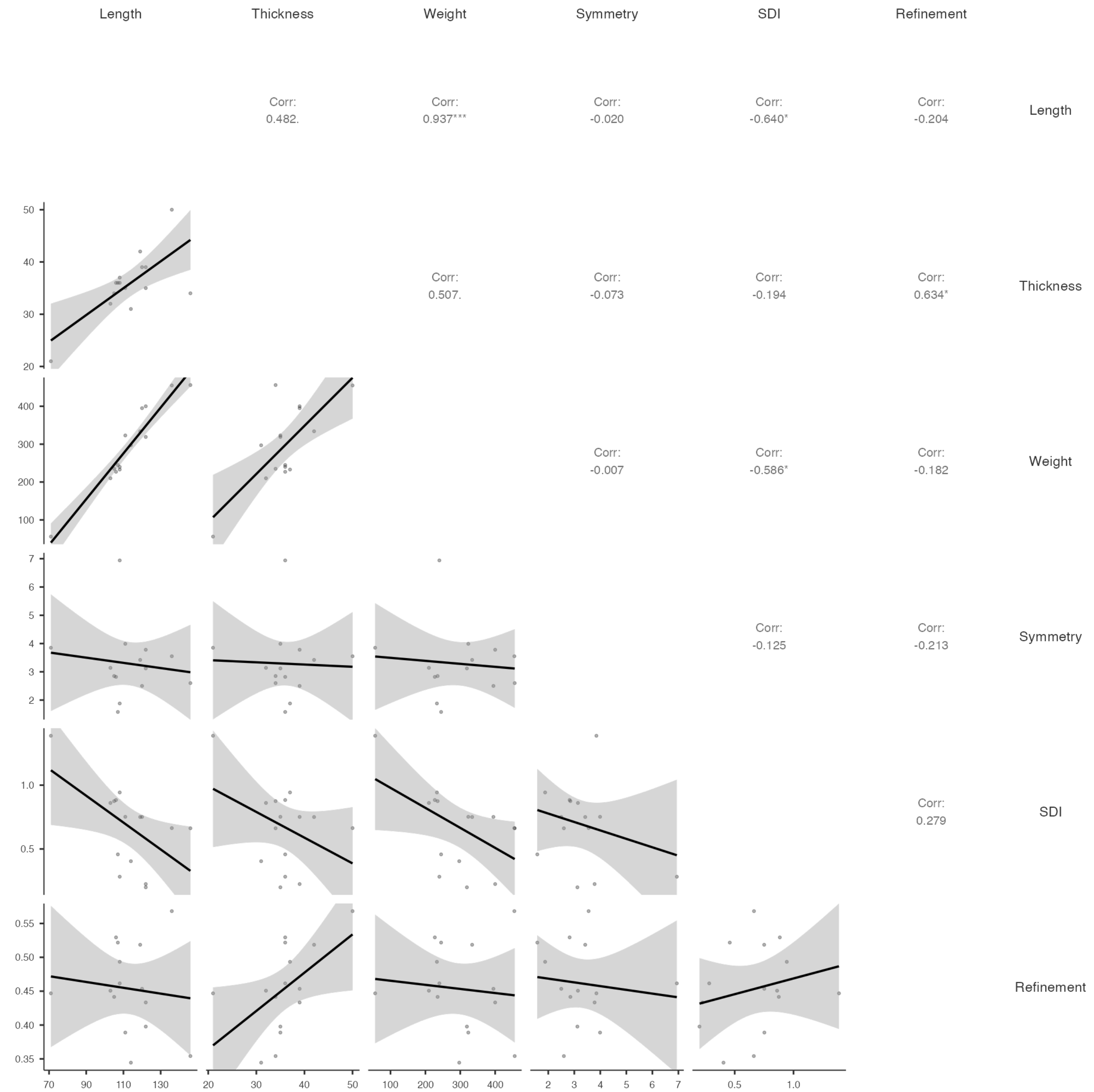

Supplement: Supplementary file 10 [file pone.0357880.s011.pdf]
